# Supplementary material for: Glyco-engineered MDCK cells display preferred receptors of H3N2 influenza absent in eggs used for vaccines
Source: Nat Commun. 2023 Oct 4;14:6178. doi: 10.1038/s41467-023-41908-0 (PMC10551000; doi:10.1038/s41467-023-41908-0)
Supplement: Supplementary file 1 — Supplementary Information [file 41467_2023_41908_MOESM1_ESM.pdf]

Supplementary information

# Glyco-engineered MDCK cells display preferred receptors of H3N2 influenza absent in eggs used for vaccines

Chika Kikuchi,[1,2,†] Aristotelis Antonopoulos,[3, †] Shengyang Wang,[1,2] Tadashi Maemura,[4] Rositsa Karamanska,[3] Chiara Lee,[3] Andrew J. Thompson,[1,2] Anne Dell,[3] Yoshihiro Kawaoka,[4,5,6,7] Stuart M. Haslam,[3,*] and James C. Paulson[1,2,*]

[1]Department of Molecular Medicine, The Scripps Research Institute, La Jolla, California, USA.

[2]Department of Immunology and Microbiology, The Scripps Research Institute, La Jolla, California, USA.

[3]Department of Life Sciences, Imperial College London, London, SW7 2AZ, UK.

[4]Influenza Research Institute, [4]Department of Pathobiological Sciences, School of Veterinary Medicine, University of Wisconsin-Madison, Madison, WI, USA.

[5]Division of Virology, Institute of Medical Science, University of Tokyo, Tokyo, Japan.

[6]The Research Center for Global Viral Diseases, National Center for Global Health and Medicine Research Institute, Tokyo, Japan.

[7]The University of Tokyo, Pandemic Preparedness, Infection and Advanced Research Center, Tokyo 162-8655, Japan.

[†] These authors contributed equally

*Correspondence: s.haslam@imperial.ac.uk (S.M.H.), jpaulson@scripps.edu (J.C.P.)

Supplementary Figures 1 – 13

Supplementary Data 1

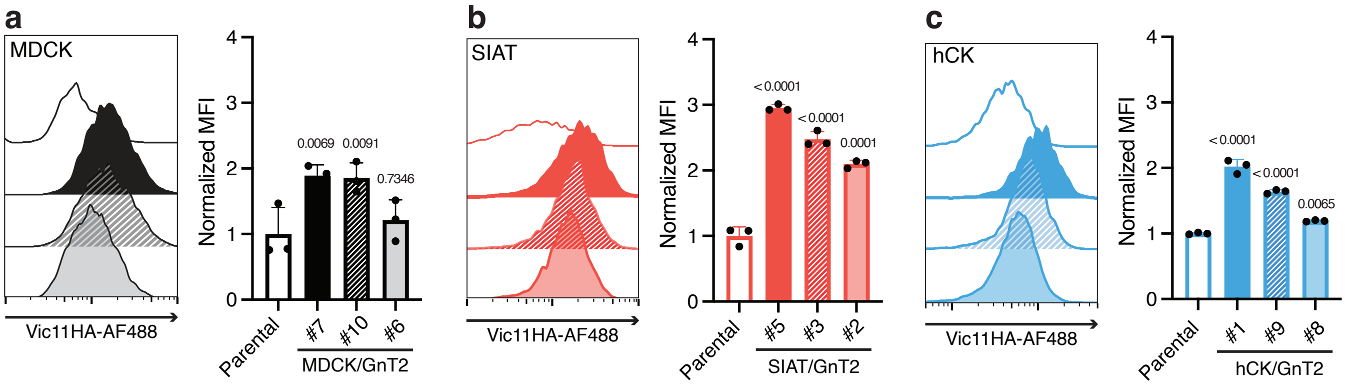

a
MDCK
Vic11HA-AF488
Normalized MFI
0
1
2
3
4
0.0069
0.0091
0.7346
Parental
#7
#10
#6
MDCK/GnT2
b
SIAT
Vic11HA-AF488
Normalized MFI
0
1
2
3
4
< 0.0001
< 0.0001
0.0001
Parental
#5
#3
#2
SIAT/GnT2
c
hCK
Vic11HA-AF488
Normalized MFI
0
1
2
3
4
< 0.0001
< 0.0001
0.0065
Parental
#1
#9
#8
hCK/GnT2

**Supplementary Figure 1. Selection of NExt cell lines.** (**a**) MDCK, (**b**) SIAT, and (**c**) hCK cells were transfected with β3GnT2 gene-coding plasmid and phenotypically screened by Vic/11 rHA binding by flow cytometry. Representitive histogram from the flow cytometric analyses of the parental untransfected cells and top 3 hits from single-clone transfectants are shown on the left. The normalized MFI from three technical replicates are shown in the bar graphs. MFI are normalized to respective parental untransfected cells. Bars indicate the mean of three replicate experiments. Error bars indicate the standard deviation. *P* values were calculated using the one-way ANOVA test with Tukey's multiple comparisons. # $P<0.0001$. Source data are provided as Source Data file.

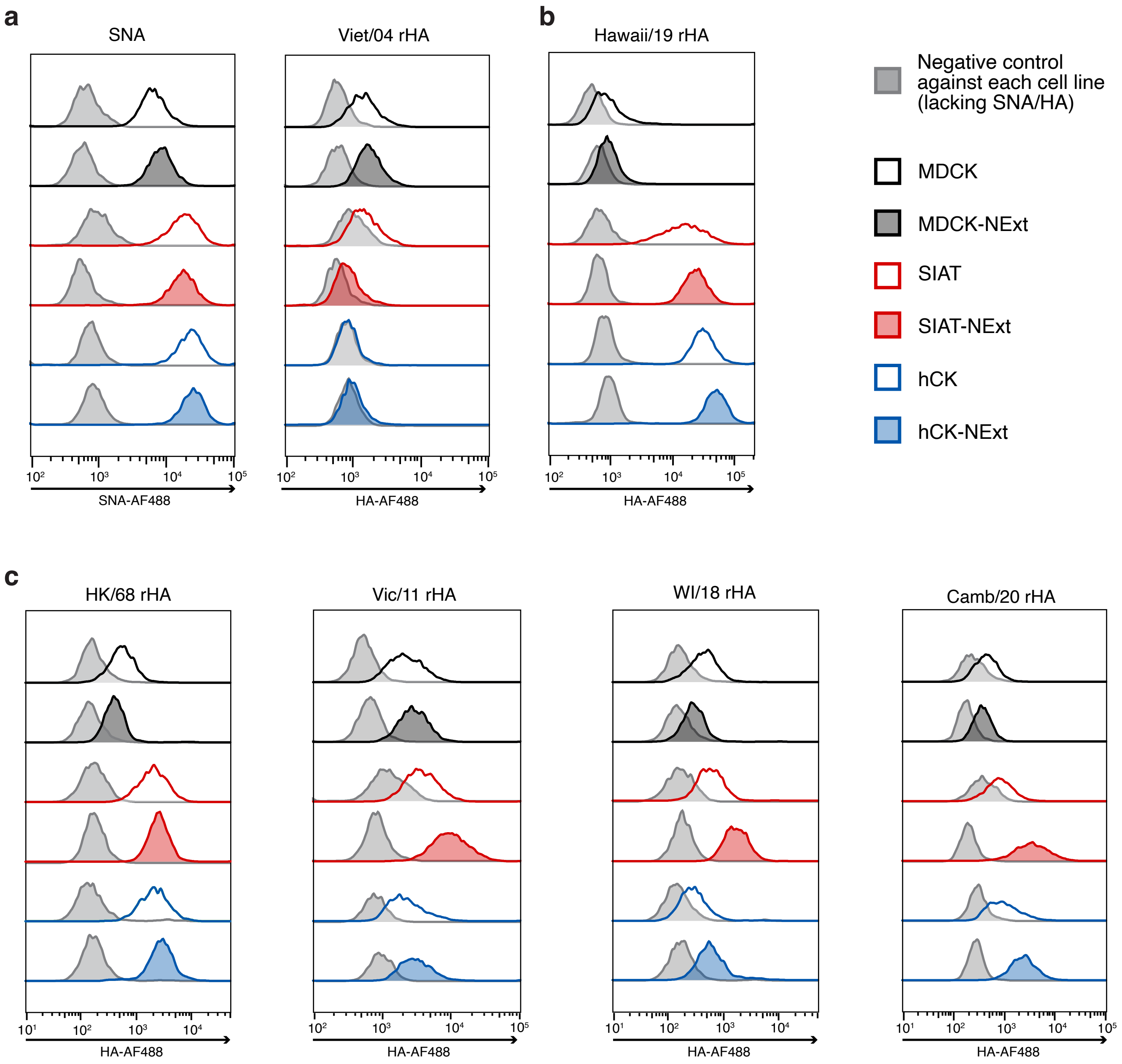

a
SNA
SNA-AF488
Viet/04 rHA
HA-AF488
b
Hawaii/19 rHA
HA-AF488
Negative control against each cell line (lacking SNA/HA)
MDCK
MDCK-NExt
SIAT
SIAT-NExt
hCK
hCK-NExt
c
HK/68 rHA
Vic/11 rHA
WI/18 rHA
Camb/20 rHA
HA-AF488

**Supplementary Figure 2. Representative histograms for SNA and rHA flow cytometry assays in Figure. 2.** Representative histograms from panels from Fig. 2 (**a**) SNA and Viet/04 rHA (**b**) Hawaii/19 rHA, and (**c**) HK/68, Vic/11, WI/18, and Camb/20 rHAs binding to MDCK, MDCK-NExt, SIAT, SIAT-NExt, hCK, and hCK-NExt cells. The gray histograms represent negative control lacking SNA/rHA. Data were analyzed by FlowJo.

# Supplementary Figure 3a

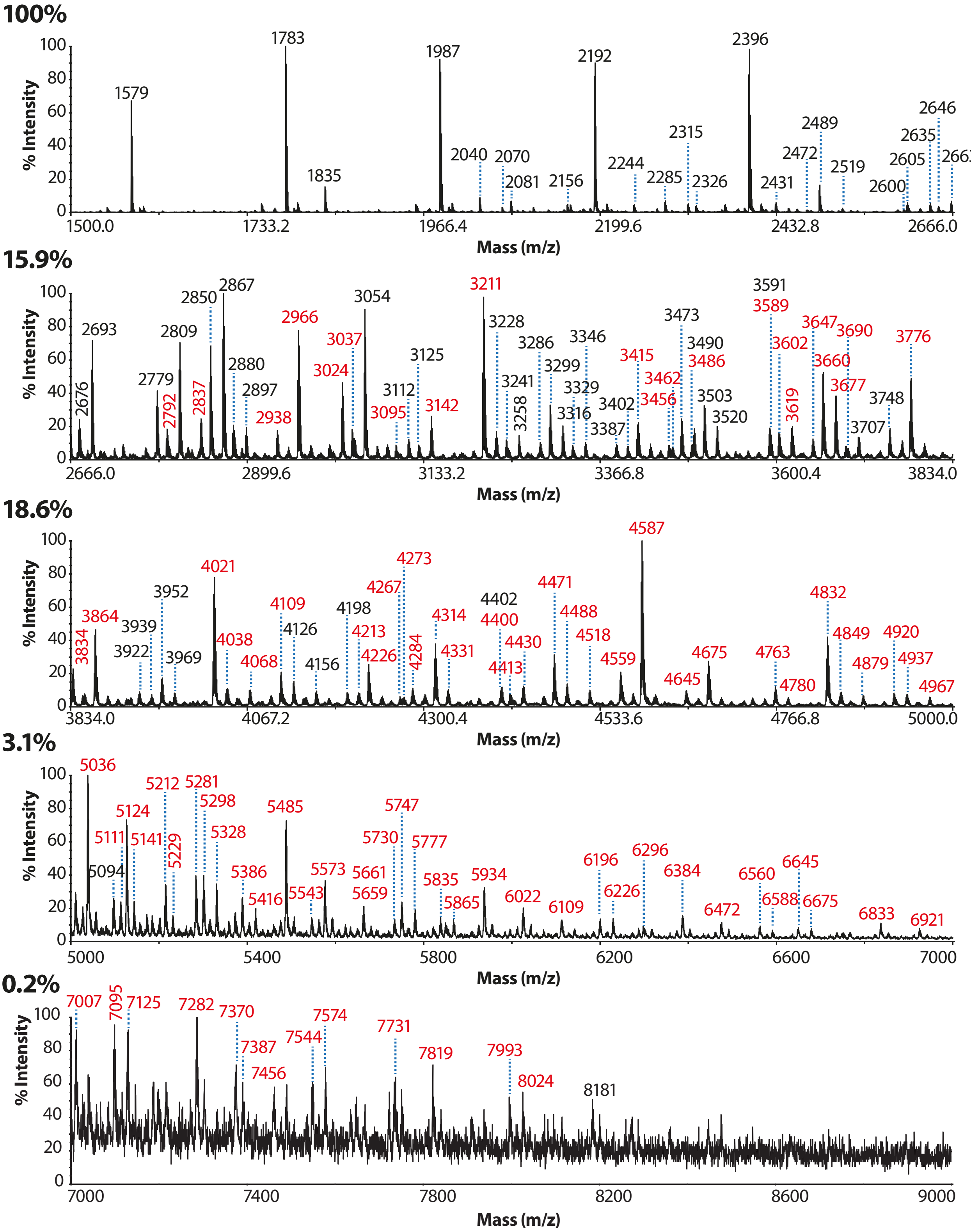

# Supplementary Figure 3b

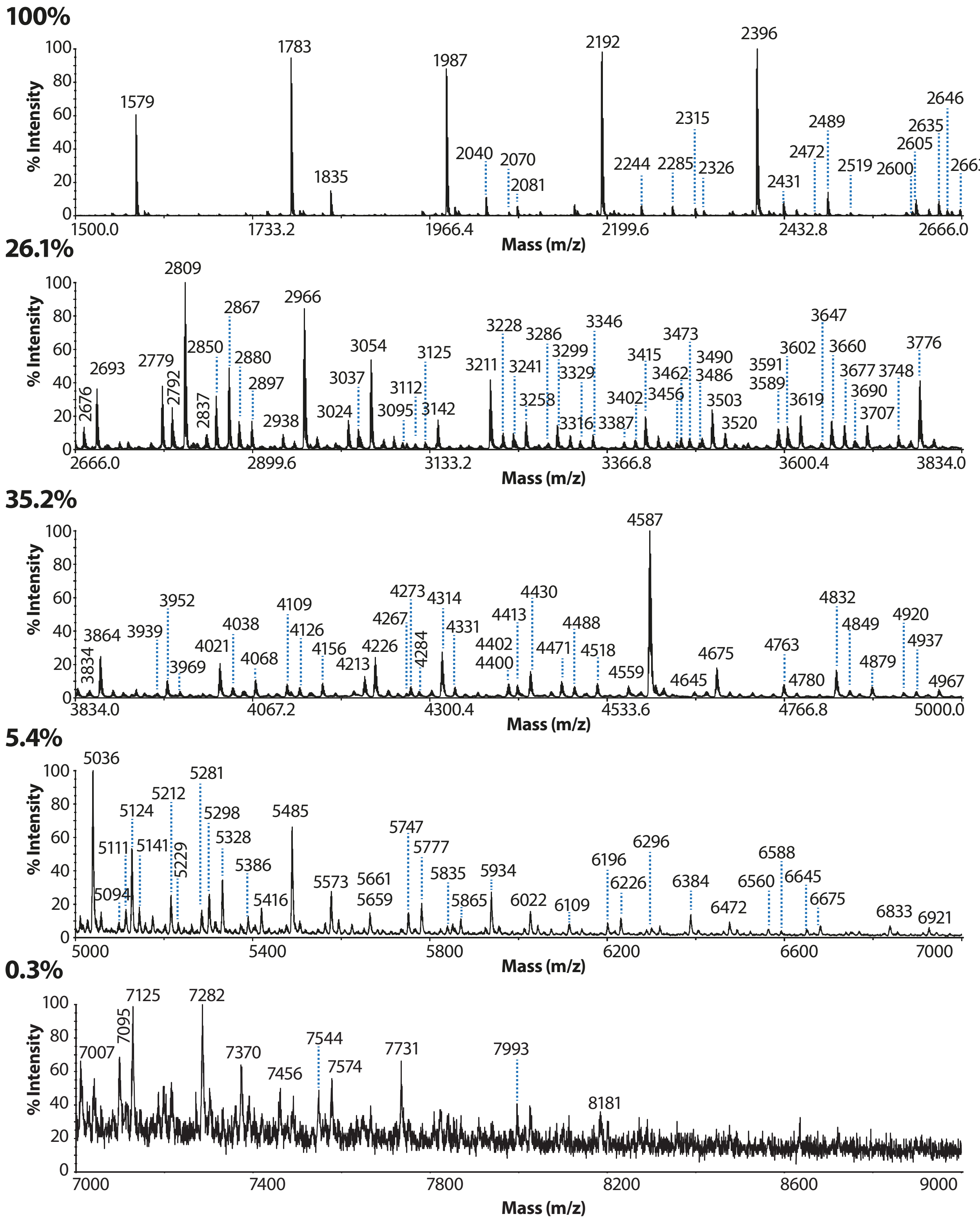

# Supplementary Figure 3c

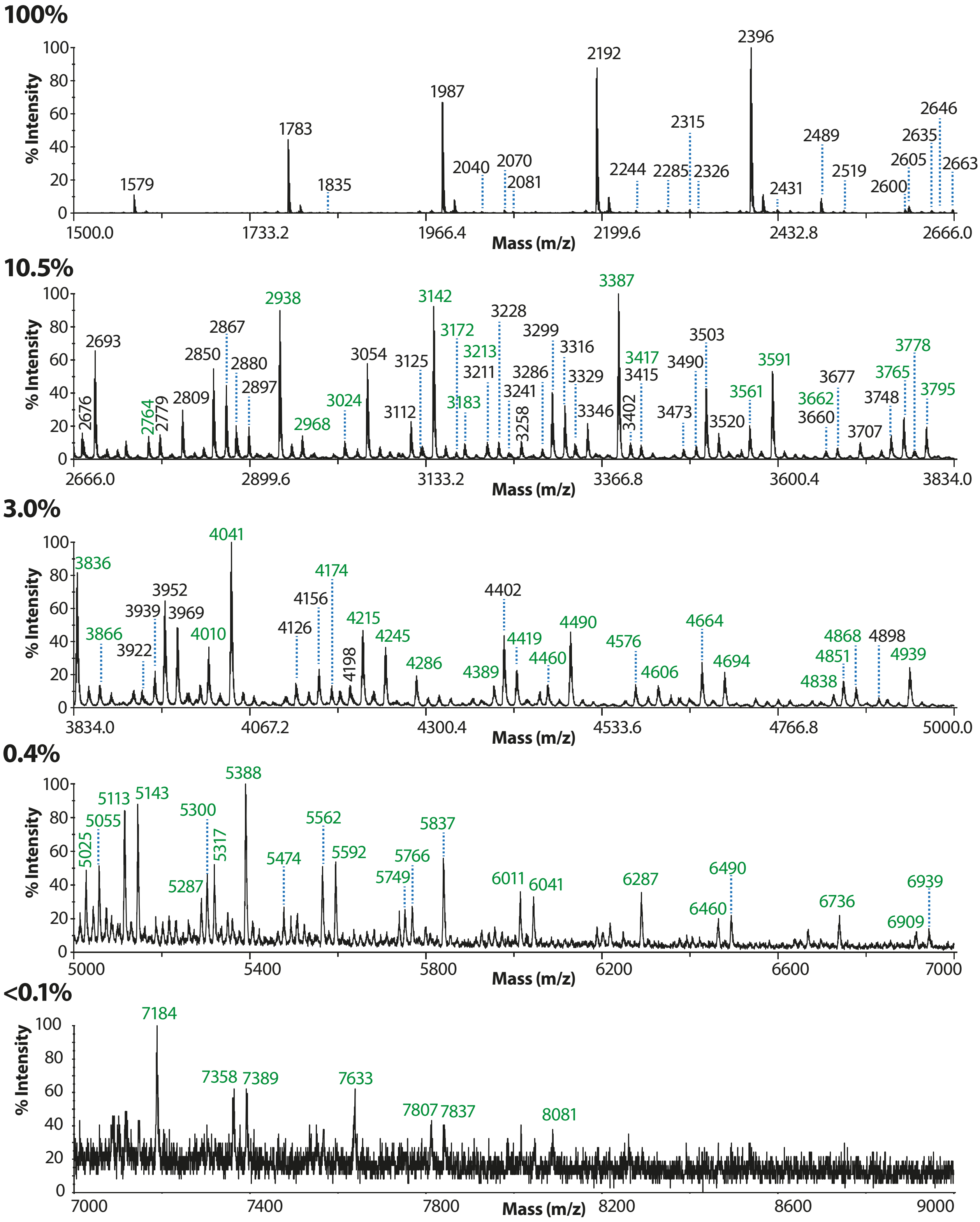

# Supplementary Figure 3d

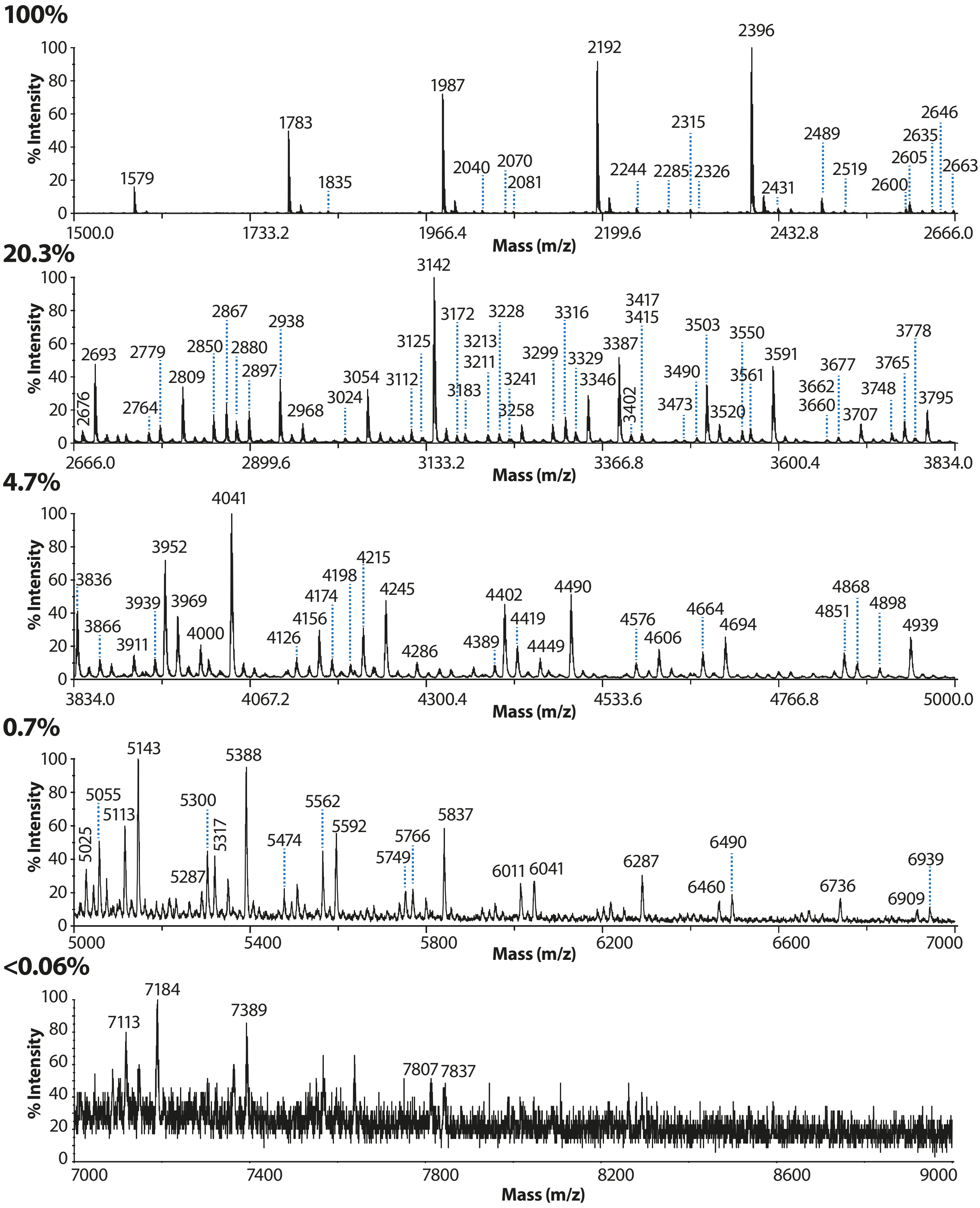

**Supplementary Figure 3. MALDI-TOF MS analysis of permethylated N-glycans from MDCK and MDCK-NExt cells, before or after the Sial-S digestion.** (**a**) MDCK; (**b**) MDCK-NExt; (**c**) MDCK + Sial-S treatment; (**d**) MDCK-NExt + Sial-S treatment. All molecular ions are $[M+Na]^+$. Putative structures are based on composition, tandem mass spectrometry, and biosynthetic knowledge. Percentages on top left of each panel correspond to the relative percentage of the maximum peak of the corresponding panel compared to the relative intensity of the maximum peak of the top panel. In (**a**) red values correspond to m/z values of the molecular ions that their relative abundance decreased after the Sial-S digestion, indication of α2,3-linked NeuAc residues (or affected by Sial-S digestion, for non sialylated N-glycans). In (**c**) green peaks correspond to peaks with increased relative abundance after the Sial-S digestion. Full structure annotations can be found in **Supplementary Fig. 4**. MS spectra exported in ASCII format are provided as Source Data file.

# Supplementary Figure 4

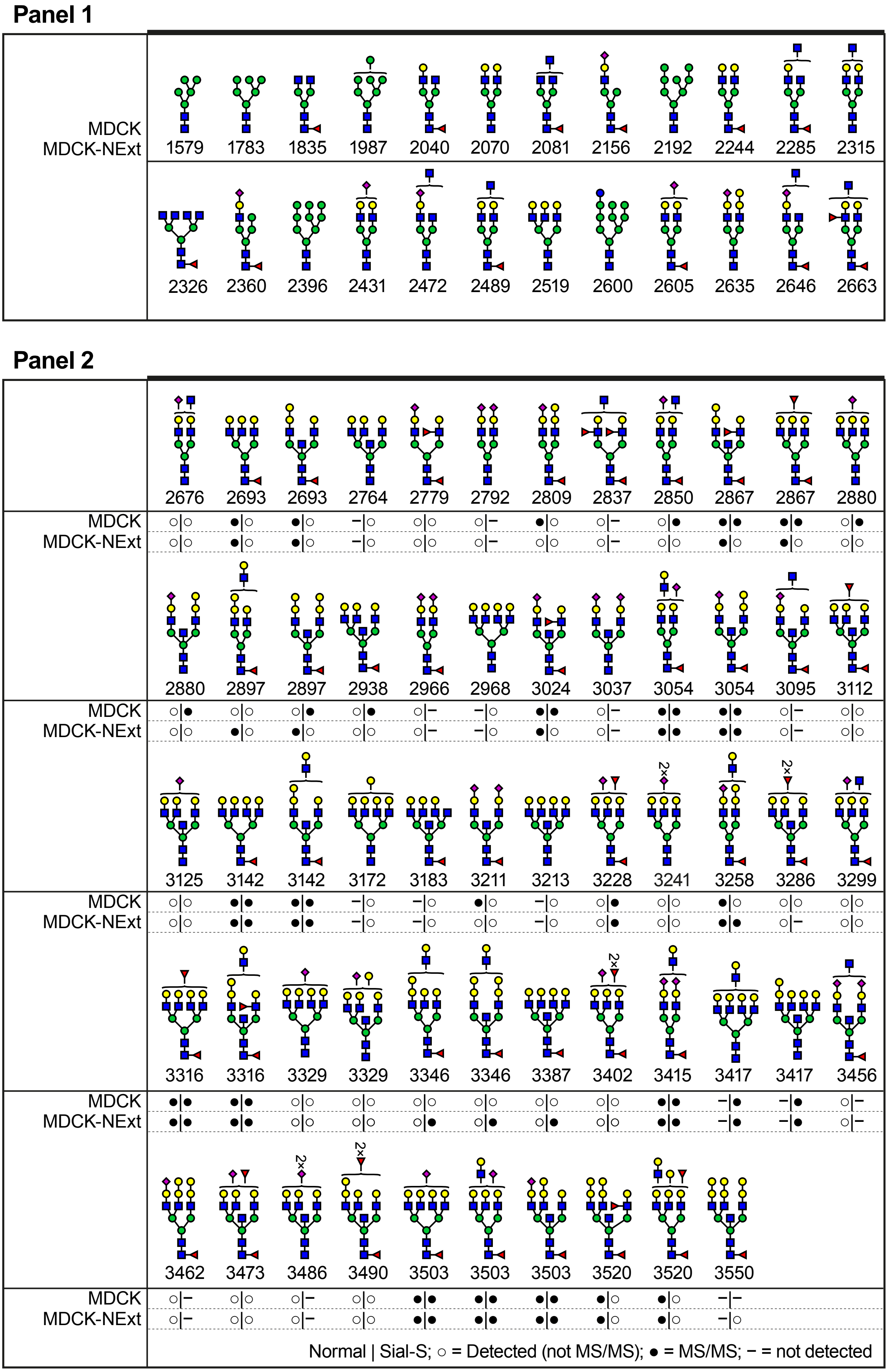

## Panel 2 *continued*

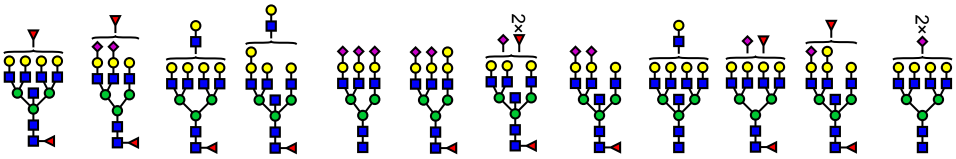


<table>
<tr><td></td><td>3561</td><td>3589</td><td>3591</td><td>3591</td><td>3602</td><td>3619</td><td>3647</td><td>3660</td><td>3662</td><td>3677</td><td>3677</td><td>3690</td></tr>
<tr><td>MDCK</td><td>−|○</td><td>●|−</td><td>○|●</td><td>○|●</td><td>○|−</td><td>○|−</td><td>○|−</td><td>○|○</td><td>−|○</td><td>●|●</td><td>●|●</td><td>○|−</td></tr>
<tr><td>MDCK-NExt</td><td>−|○</td><td>●|−</td><td>●|●</td><td>●|●</td><td>○|−</td><td>○|−</td><td>○|−</td><td>○|○</td><td>−|○</td><td>○|●</td><td>○|●</td><td>○|−</td></tr>
</table>

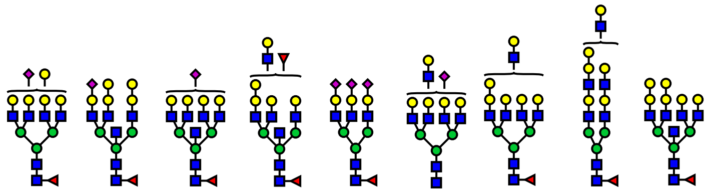

<table>
<tr><td></td><td>3707</td><td>3707</td><td>3748</td><td>3765</td><td>3776</td><td>3778</td><td>3795</td><td>3795</td><td>3795</td></tr>
<tr><td>MDCK</td><td>●|○</td><td>●|○</td><td>○|○</td><td>−|●</td><td>○|−</td><td>−|○</td><td>−|●</td><td>−|●</td><td>−|●</td></tr>
<tr><td>MDCK-NExt</td><td>○|●</td><td>○|●</td><td>○|○</td><td>−|●</td><td>○|−</td><td>−|○</td><td>−|●</td><td>−|●</td><td>−|●</td></tr>
</table>

Normal | Sial-S; ○ = Detected (not MS/MS); ● = MS/MS; − = not detected

## Panel 3

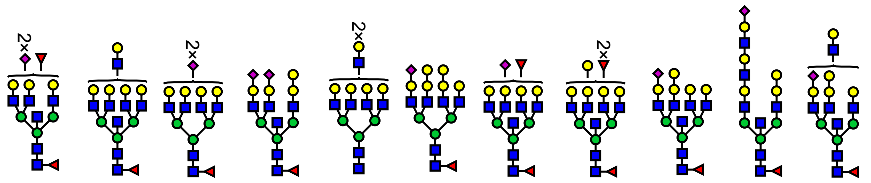


<table>
<tr><td></td><td>3834</td><td>3836</td><td>3864</td><td>3864</td><td>3866</td><td>3911</td><td>3922</td><td>3939</td><td>3952</td><td>3952</td><td>3952</td></tr>
<tr><td>MDCK</td><td>○|−</td><td>−|○</td><td>●|−</td><td>●|−</td><td>−|○</td><td>−|−</td><td>○|○</td><td>○|○</td><td>●|○</td><td>●|○</td><td>●|○</td></tr>
<tr><td>MDCK-NExt</td><td>○|−</td><td>−|○</td><td>○|−</td><td>○|−</td><td>−|○</td><td>−|○</td><td>−|−</td><td>○|○</td><td>●|●</td><td>●|●</td><td>●|●</td></tr>
</table>

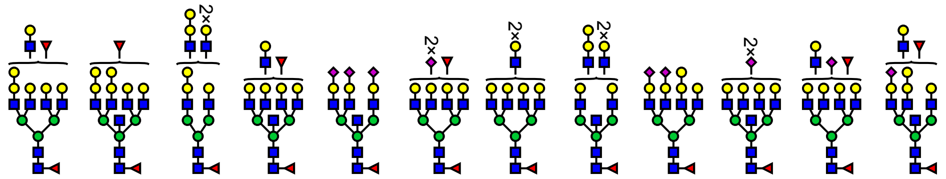


<table>
<tr><td></td><td>3969</td><td>3969</td><td>4000</td><td>4011</td><td>4021</td><td>4038</td><td>4041</td><td>4041</td><td>4068</td><td>4109</td><td>4126</td><td>4126</td></tr>
<tr><td>MDCK</td><td>○|○</td><td>○|○</td><td>−|−</td><td>−|○</td><td>●|−</td><td>○|−</td><td>−|●</td><td>−|●</td><td>○|−</td><td>○|−</td><td>○|○</td><td>○|○</td></tr>
<tr><td>MDCK-NExt</td><td>○|○</td><td>○|○</td><td>−|○</td><td>−|−</td><td>●|−</td><td>○|−</td><td>−|●</td><td>−|●</td><td>○|−</td><td>○|−</td><td>○|○</td><td>○|○</td></tr>
</table>

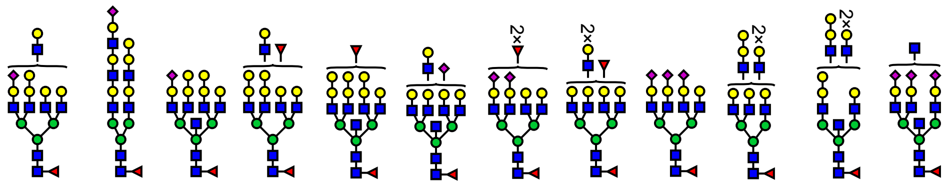


<table>
<tr><td></td><td>4156</td><td>4156</td><td>4156</td><td>4174</td><td>4174</td><td>4198</td><td>4213</td><td>4215</td><td>4226</td><td>4245</td><td>4245</td><td>4267</td></tr>
<tr><td>MDCK</td><td>●|○</td><td>●|○</td><td>●|○</td><td>−|○</td><td>−|○</td><td>○|○</td><td>○|−</td><td>−|○</td><td>○|−</td><td>−|○</td><td>−|○</td><td>○|−</td></tr>
<tr><td>MDCK-NExt</td><td>●|○</td><td>●|○</td><td>●|○</td><td>−|○</td><td>−|○</td><td>−|○</td><td>○|−</td><td>−|●</td><td>○|−</td><td>−|●</td><td>−|●</td><td>○|−</td></tr>
</table>

Normal | Sial-S; ○ = Detected (not MS/MS); ● = MS/MS; − = not detected

**Panel 3** *continued*

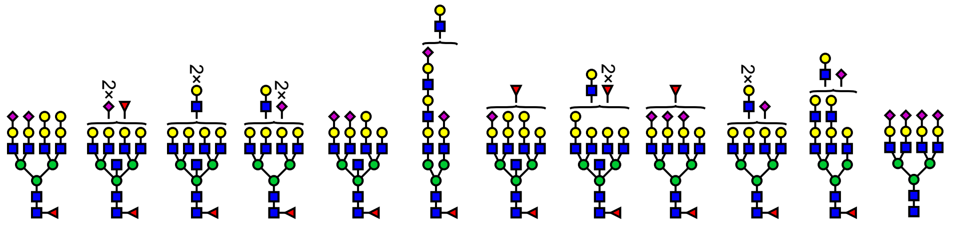

| | 4273 | 4284 | 4286 | 4314 | 4314 | 4314 | 4331 | 4389 | 4400 | 4402 | 4402 | 4413 |
|---|---|---|---|---|---|---|---|---|---|---|---|---|
| MDCK | ○\|− | ○\|− | −\|○ | ●\|− | ●\|− | ●\|− | ○\|− | −\|○ | ○\|− | ○\|○ | ○\|○ | ○\|− |
| MDCK-NExt | ○\|− | ○\|− | −\|○ | ●\|− | ●\|− | ●\|− | ○\|− | −\|○ | ○\|− | ○\|● | ○\|● | ○\|− |

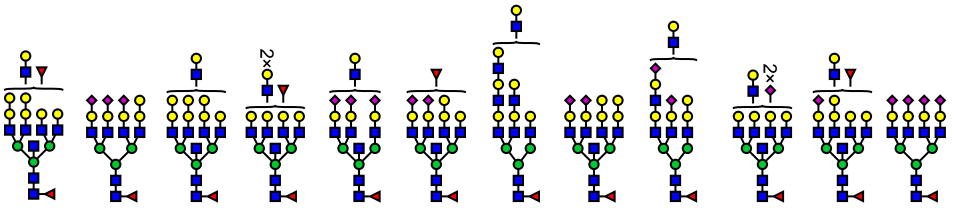

| | 4419 | 4430 | 4449 | 4460 | 4471 | 4488 | 4490 | 4518 | 4518 | 4559 | 4576 | 4587 |
|---|---|---|---|---|---|---|---|---|---|---|---|---|
| MDCK | −\|○ | ○\|− | −\|− | −\|○ | ○\|− | ○\|− | −\|● | ○\|− | ○\|− | ○\|− | −\|○ | ○\|− |
| MDCK-NExt | −\|○ | ○\|− | −\|○ | −\|− | ○\|− | ○\|− | −\|● | ○\|− | ○\|− | ○\|− | −\|○ | ●\|− |

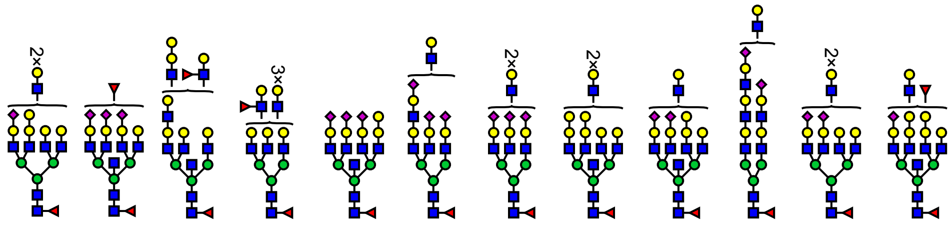

| | 4606 | 4645 | 4664 | 4664 | 4675 | 4675 | 4675 | 4694 | 4763 | 4763 | 4763 | 4780 |
|---|---|---|---|---|---|---|---|---|---|---|---|---|
| MDCK | −\|○ | ○\|− | −\|● | −\|● | ●\|− | ●\|− | ●\|− | −\|● | ●\|− | ●\|− | ●\|− | ○\|− |
| MDCK-NExt | −\|○ | ○\|− | −\|● | −\|● | ●\|− | ●\|− | ●\|− | −\|● | ○\|− | ○\|− | ○\|− | ○\|− |

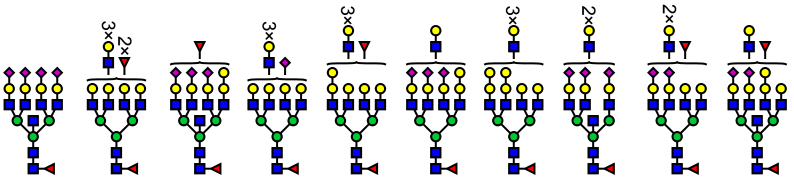

| | 4832 | 4838 | 4849 | 4851 | 4868 | 4879 | 4898 | 4920 | 4937 | 4937 |
|---|---|---|---|---|---|---|---|---|---|---|
| MDCK | ●\|− | −\|○ | ○\|− | −\|○ | −\|○ | ○\|− | −\|○ | ○\|− | ○\|− | ○\|− |
| MDCK-NExt | ●\|− | −\|− | ○\|− | −\|○ | −\|○ | ○\|− | −\|○ | ○\|− | ○\|− | ○\|− |

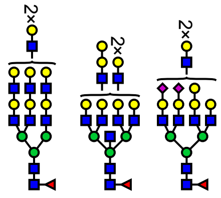

| | 4939 | 4939 | 4967 |
|---|---|---|---|
| MDCK | −\|● | −\|● | ○\|− |
| MDCK-NExt | −\|● | −\|● | ○\|− |

Normal | Sial-S; ○ = Detected (not MS/MS); ● = MS/MS; − = not detected

## Panel 4

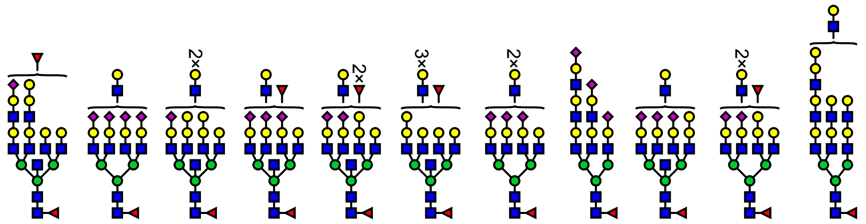

| | 5025 | 5036 | 5055 | 5094 | 5111 | 5113 | 5124 | 5124 | 5124 | 5141 | 5143 |
|---|---|---|---|---|---|---|---|---|---|---|---|
| MDCK | −\|○ | ●\|− | −\|○ | ○\|− | ○\|− | −\|○ | ●\|− | ●\|− | ●\|− | ○\|− | −\|○ |
| MDCK-NExt | −\|○ | ●\|− | −\|○ | ○\|− | ○\|− | −\|○ | ○\|− | ○\|− | ○\|− | ○\|− | −\|● |

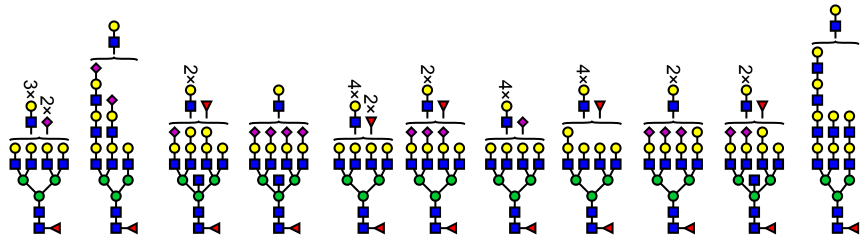

| | 5212 | 5212 | 5229 | 5281 | 5287 | 5298 | 5300 | 5317 | 5328 | 5386 | 5388 |
|---|---|---|---|---|---|---|---|---|---|---|---|
| MDCK | ●\|− | ●\|− | ○\|− | ○\|− | −\|○ | ○\|− | −\|○ | −\|○ | ○\|− | ○\|− | −\|● |
| MDCK-NExt | ○\|− | ○\|− | ○\|− | ○\|− | −\|○ | ○\|− | −\|○ | −\|○ | ○\|− | ○\|− | −\|● |

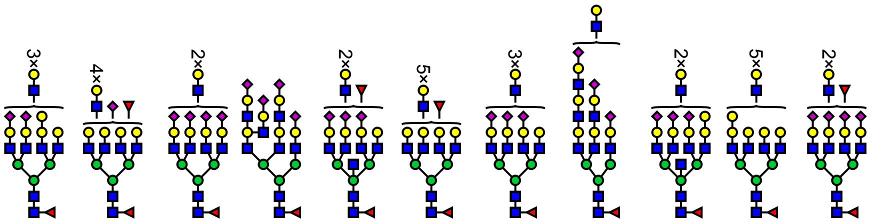

| | 5416 | 5474 | 5485 | 5485 | 5543 | 5562 | 5573 | 5573 | 5573 | 5592 | 5659 |
|---|---|---|---|---|---|---|---|---|---|---|---|
| MDCK | ○\|− | −\|○ | ●\|− | ●\|− | ○\|− | −\|○ | ○\|− | ○\|− | ○\|− | −\|○ | ○\|− |
| MDCK-NExt | ○\|− | −\|○ | ●\|− | ●\|− | −\|− | −\|● | ●\|− | ●\|− | ●\|− | −\|● | ●\|− |

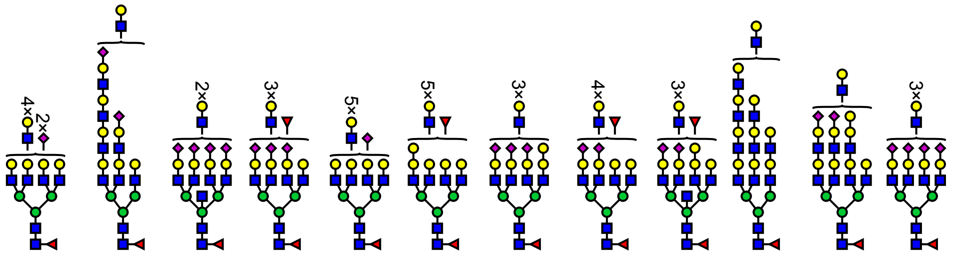

| | 5661 | 5661 | 5730 | 5747 | 5749 | 5766 | 5777 | 5835 | 5835 | 5837 | 5865 | 5934 |
|---|---|---|---|---|---|---|---|---|---|---|---|---|
| MDCK | ○\|− | ○\|− | ○\|− | ○\|− | −\|○ | −\|○ | ○\|− | ○\|− | ○\|− | −\|● | ○\|− | ●\|− |
| MDCK-NExt | ●\|− | ●\|− | −\|− | ○\|− | −\|○ | −\|● | ○\|− | ○\|− | ○\|− | −\|● | ○\|− | ●\|− |

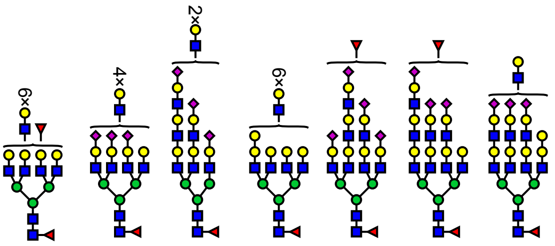

| | 6011 | 6022 | 6022 | 6041 | 6109 | 6196 | 6226 |
|---|---|---|---|---|---|---|---|
| MDCK | −\|● | ○\|− | ○\|− | −\|○ | ○\|− | ○\|− | ○\|− |
| MDCK-NExt | −\|● | ●\|− | ●\|− | −\|○ | ●\|− | ○\|− | ○\|− |

Normal | Sial-S; ○ = Detected (not MS/MS); ● = MS/MS; − = not detected

## Panel 4 *continued*

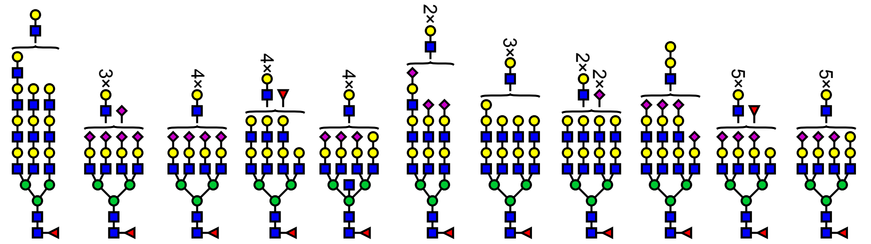

| | 6287 | 6296 | 6384 | 6460 | 6472 | 6472 | 6490 | 6560 | 6588 | 6645 | 6675 |
|---|---|---|---|---|---|---|---|---|---|---|---|
| MDCK | −\|● | ○\|− | ○\|− | −\|○ | ○\|− | ○\|− | −\|○ | ○\|− | ○\|− | ○\|− | ○\|− |
| MDCK-NExt | −\|● | ○\|− | ●\|− | −\|○ | ○\|− | ○\|− | −\|● | ○\|− | ○\|− | ○\|− | ○\|− |

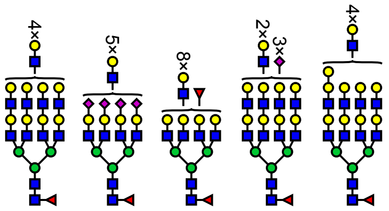

| | 6736 | 6833 | 6909 | 6921 | 6939 |
|---|---|---|---|---|---|
| MDCK | −\|○ | ○\|− | −\|○ | ○\|− | −\|○ |
| MDCK-NExt | −\|● | ●\|− | −\|○ | ○\|− | −\|○ |

Normal | Sial-S; ○ = Detected (not MS/MS); ● = MS/MS; − = not detected

## Panel 5

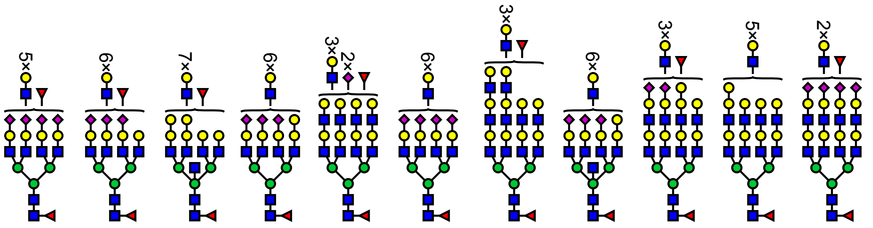

| | 7007 | 7095 | 7113 | 7125 | 7184 | 7282 | 7358 | 7370 | 7387 | 7389 | 7456 |
|---|---|---|---|---|---|---|---|---|---|---|---|
| MDCK | ○\|− | ○\|− | −\|− | ○\|− | −\|○ | ○\|− | −\|○ | ○\|− | ○\|− | −\|○ | ○\|− |
| MDCK-NExt | ○\|− | ○\|− | −\|○ | ○\|− | −\|○ | ○\|− | −\|− | ○\|− | −\|− | −\|○ | ○\|− |

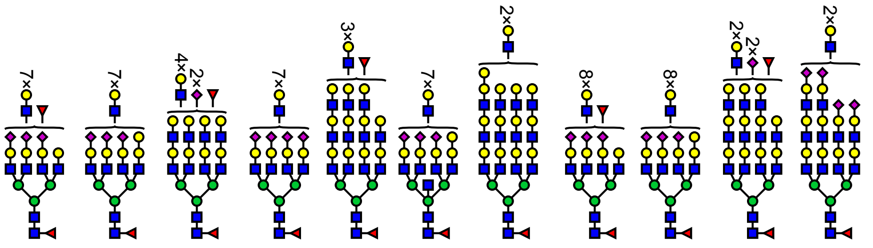

| | 7544 | 7574 | 7633 | 7731 | 7807 | 7819 | 7837 | 7993 | 8024 | 8081 | 8181 |
|---|---|---|---|---|---|---|---|---|---|---|---|
| MDCK | ○\|− | ○\|− | −\|○ | ○\|− | −\|○ | ○\|− | −\|○ | ○\|− | ○\|− | −\|○ | ○\|− |
| MDCK-NExt | ○\|− | ○\|− | −\|− | ○\|− | −\|○ | −\|− | −\|○ | ○\|− | −\|− | −\|− | ○\|− |

Normal | Sial-S; ○ = Detected (not MS/MS); ● = MS/MS; − = not detected

**Supplementary Figure 4. Structures of N-glycans found on MDCK and MDCK-NExt cells, before or after the Sial-S digestion.** Putative structures are based on composition, biosynthetic knowledge and tandem mass spectrometry where available. Cartoon structures were drawn according to the Symbol Nomenclature for Glycans (SNFG) (Neelamegham et al., 2019 Glycobiology 29, 620-624) guidelines. Structures are organized into panels according to the presentation of the mass spectra found in the corresponding Supplementary Figures. Value under each putative structure corresponds to the molecular ion $[M+Na]^+$ m/z value detected in the corresponding **Supplementary Fig. 3** (**a**, MDCK; **b**, MDCK-NExt; **c**, MDCK + Sial-S treatment; **d**, MDCK-NExt + Sial-S treatment). Dash ("⁻") corresponds to non-detected structure, open circle ("○") corresponds to detected structure, but not been subjected to MALDI-TOF/TOF MS/MS analysis, solid circle ("●") corresponds to a detected structure subjected to MALDI-TOF/TOF MS/MS analysis. The symbols before the vertical line, indicate the detection and/or MS/MS status for the MDCK or MDCK-NExt N-glycans, while the symbols after the vertical line, indicate the detected and/or MS/MS status for the MDCK+Sial-S or MDCK-NExt+Sial-S N-glycans. MALDI TOF/TOF MS/MS analysis on selected molecular ions can be found on the **Supplementary Fig. 6** (MDCK panels). Structures above the bracket have not unequivocally been defined. Panel 1 range, m/z 1500 to 2666; Panel 2 range, m/z 2666 to 3834; Panel 3 range, m/z 3834 to 5000Panel 4 range, m/z 5000 to 7000; and Panel 5 range, m/z 7000 to 9000.

# Supplementary Figure 5

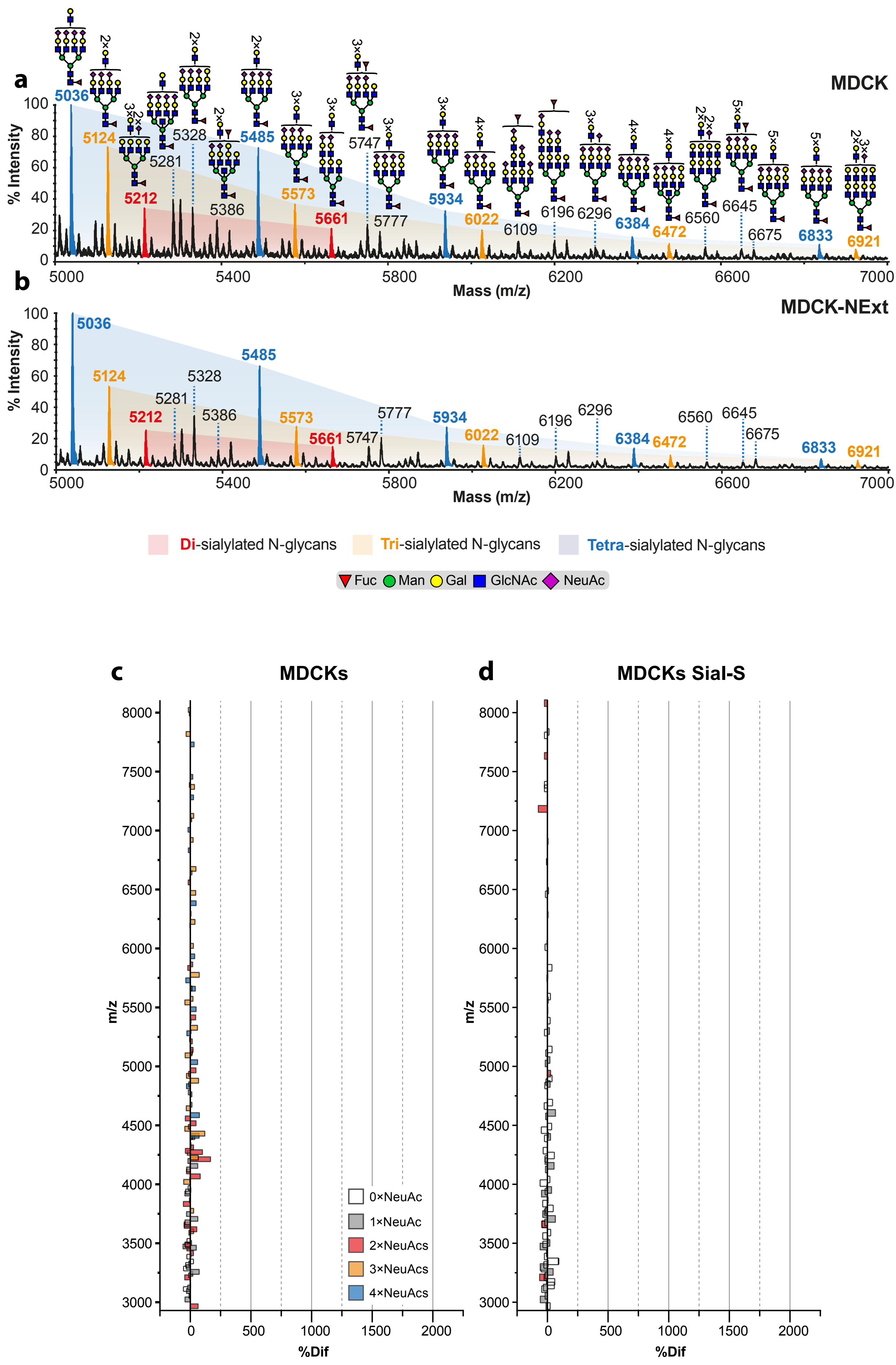

**Supplementary Figure 5. Partial MALDI-TOF mass spectra of MDCK N-glycans**. MALDI-TOF MS analysis of permethylated N-glycans from (**a**) MDCK; (**b**) MDCK-NExt. Red-, yellow- and blue- shaded areas highlight the distribution shift of poly-LacNAcs on bi-, tri- and tetra-sialylated N- glycans respectively. Same colored peaks differ only in the number of LacNAcs present. All molecular ions are $[M+Na]^+$. Putative structures are based on composition, tandem mass spectrometry, and biosynthetic knowledge. Annotated structures were drawn according to the Symbol Nomenclature for Glycans (SNFG) guidelines.[55] Structures above the bracket have not unequivocally been defined. Shaded areas were manually inserted to assist clarity. Full MALDI- TOF mass spectra of the N-glycans can be found in **Supplementary Fig. 3** and full annotations in **Supplementary Fig. 4**. Full methods for MS analysis can be found in Materials and Methods. (**c,d**) Bar graph representation of percent (%) difference of glycan mass abundance calculated as a percent of total ion intensity peak area between (**c**) MDCK vs MDCK-NExt cells and (**d**) MDCK vs MDCK-NExt cells after Sial-S treatment. Percent difference of the molecular ions corresponding to complex N-glycans (m/z 2966 and above) calculated after normalizing the relative intensity of each molecular ion to the sum of their relative intensities. The quantitated data can be found in **Supplementary Data 1**.

# Supplementary Figure 6

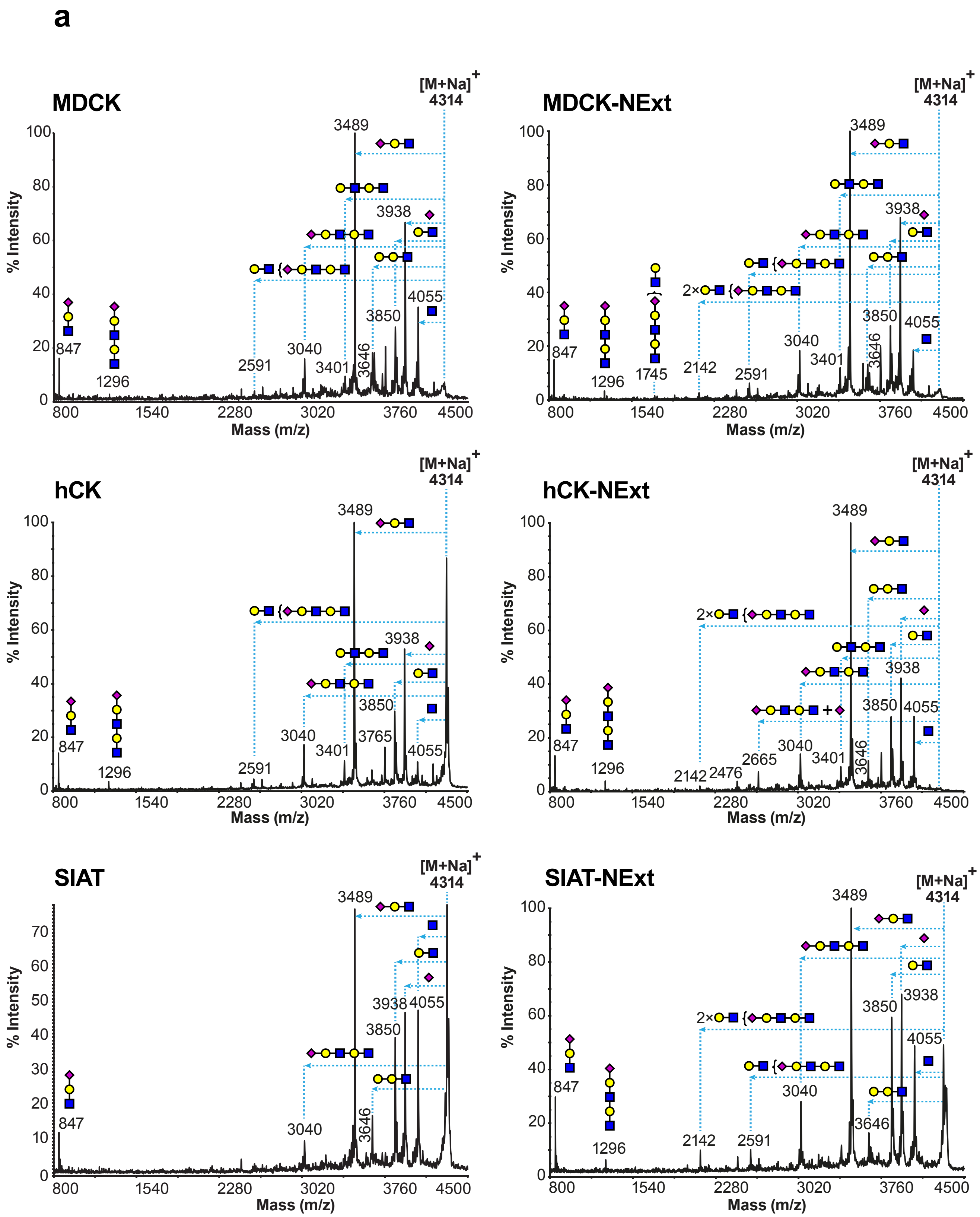

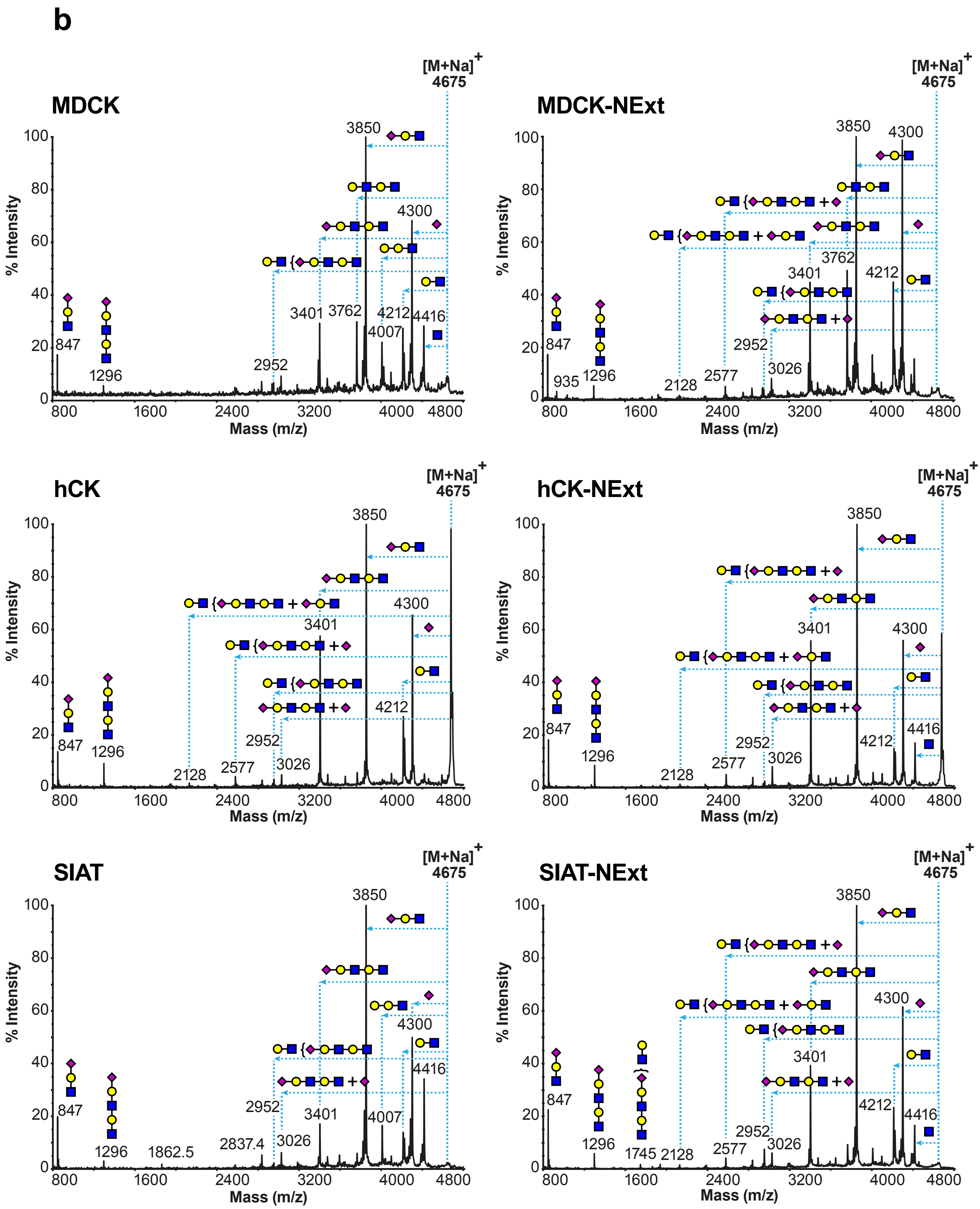
b
MDCK
MDCK-NExt
hCK
hCK-NExt
SIAT
SIAT-NExt
[M+Na]+
4675
% Intensity
Mass (m/z)
3850
4300
3401
3762
4212
4416
4007
2952
3026
2577
2128
1296
847
935
1745
1862.5
2837.4

c

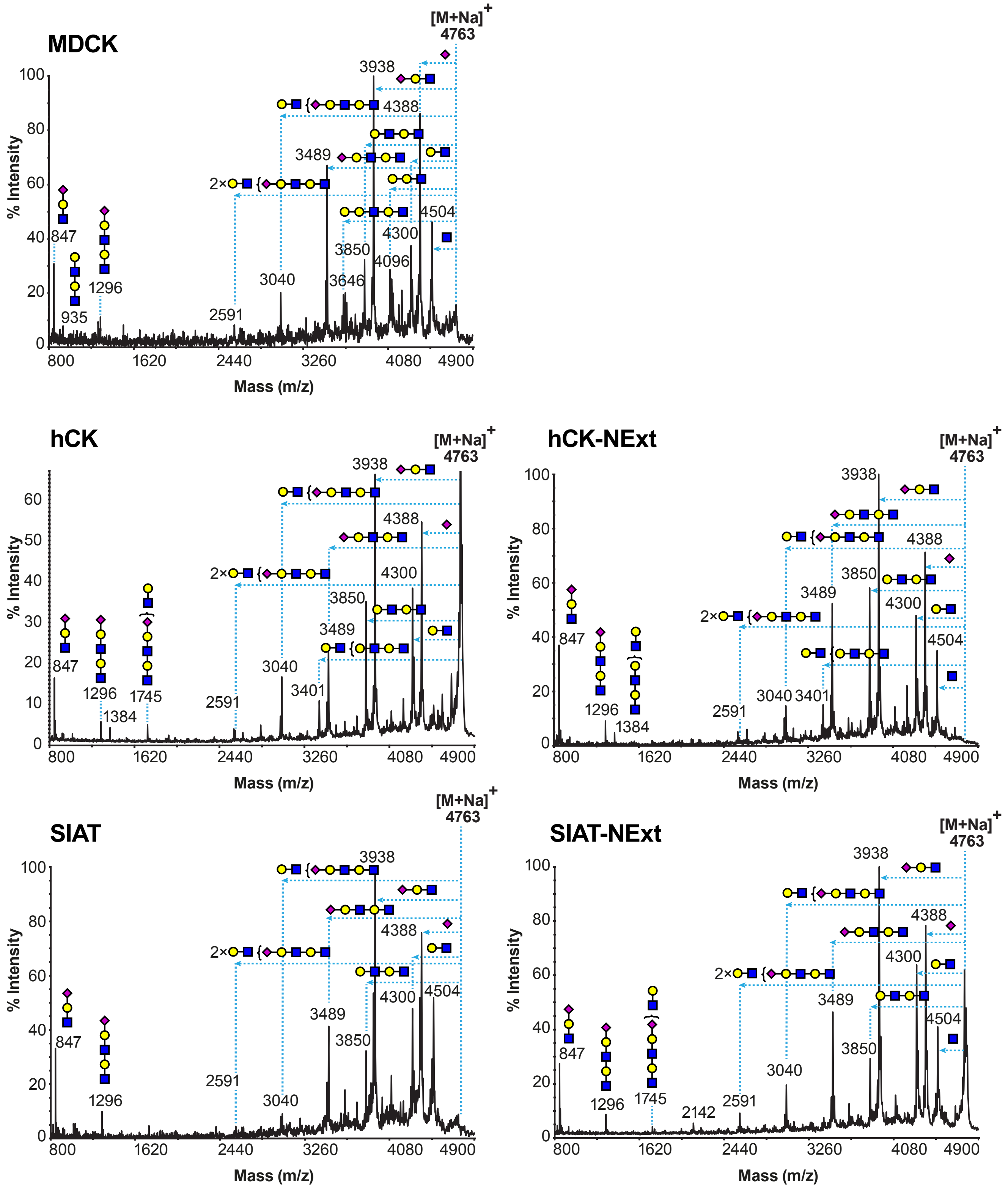
MDCK
hCK
hCK-NExt
SIAT
SIAT-NExt
[M+Na]+
4763
% Intensity
Mass (m/z)
800
1620
2440
3260
4080
4900
847
935
1296
1384
1745
2142
2591
3040
3401
3489
3646
3850
3938
4096
4300
4388
4504
2×

**d**

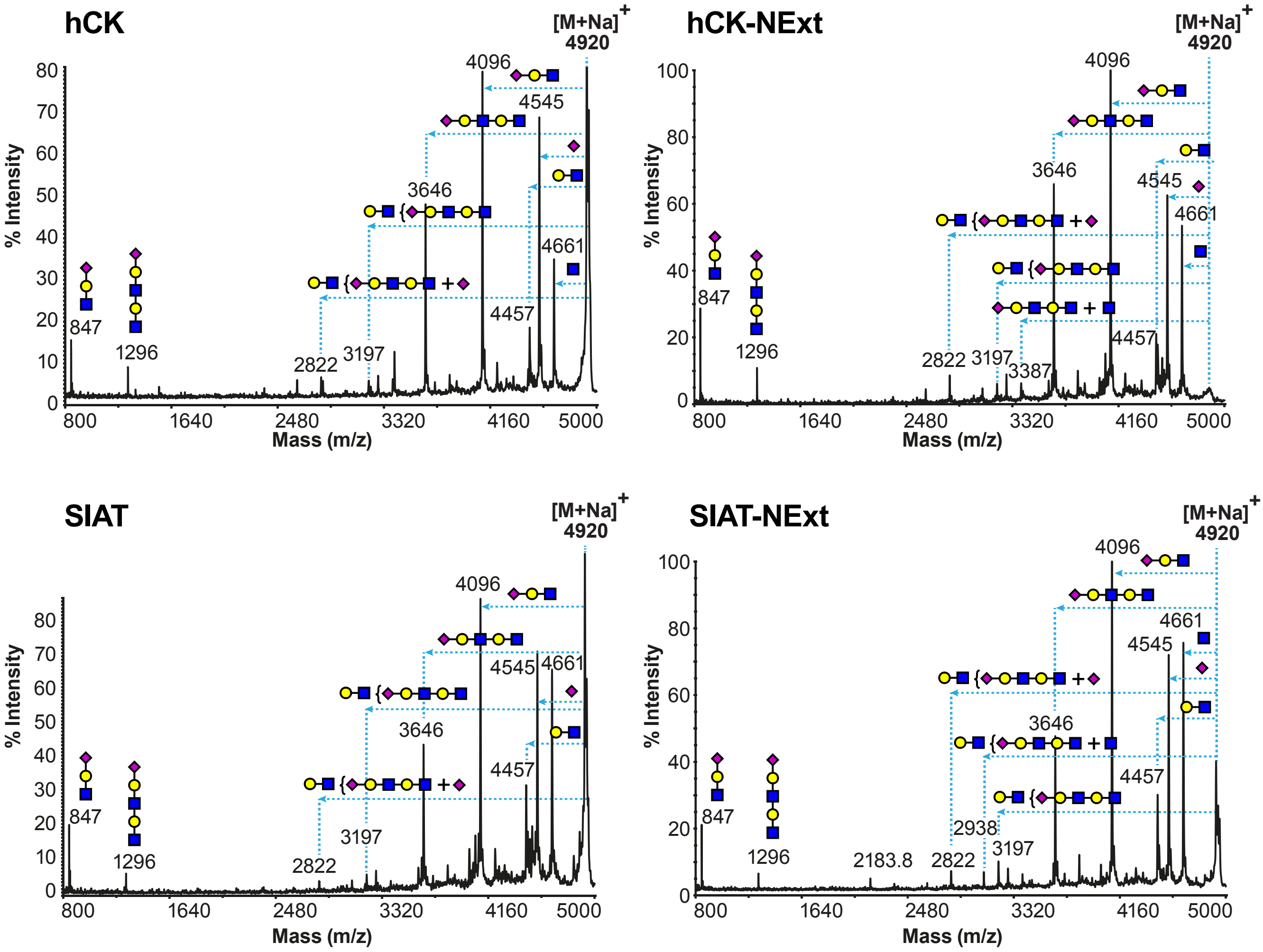

hCK
[M+Na]+
4920
% Intensity
Mass (m/z)
847
1296
2822
3197
3646
4096
4457
4545
4661
hCK-NExt
[M+Na]+
4920
% Intensity
Mass (m/z)
847
1296
2822
3197
3387
3646
4096
4457
4545
4661
SIAT
[M+Na]+
4920
% Intensity
Mass (m/z)
847
1296
2822
3197
3646
4096
4457
4545
4661
SIAT-NExt
[M+Na]+
4920
% Intensity
Mass (m/z)
847
1296
2183.8
2822
2938
3197
3646
4096
4457
4545
4661

e

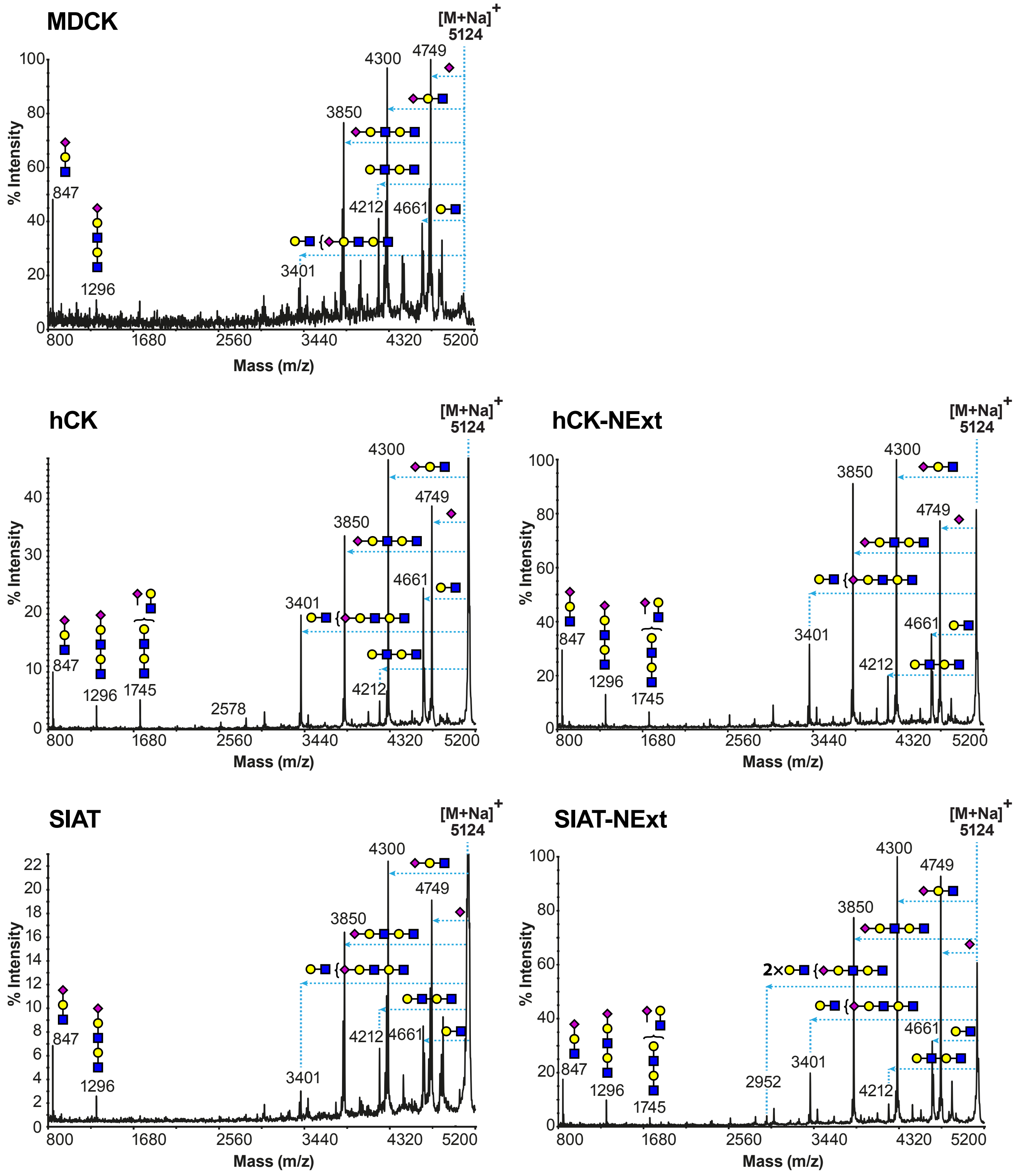
MDCK
[M+Na]+
5124
4749
4300
3850
847
1296
3401
4212
4661
% Intensity
Mass (m/z)
800
1680
2560
3440
4320
5200
hCK
hCK-NExt
2578
1745
SIAT
SIAT-NExt
2×
2952

f

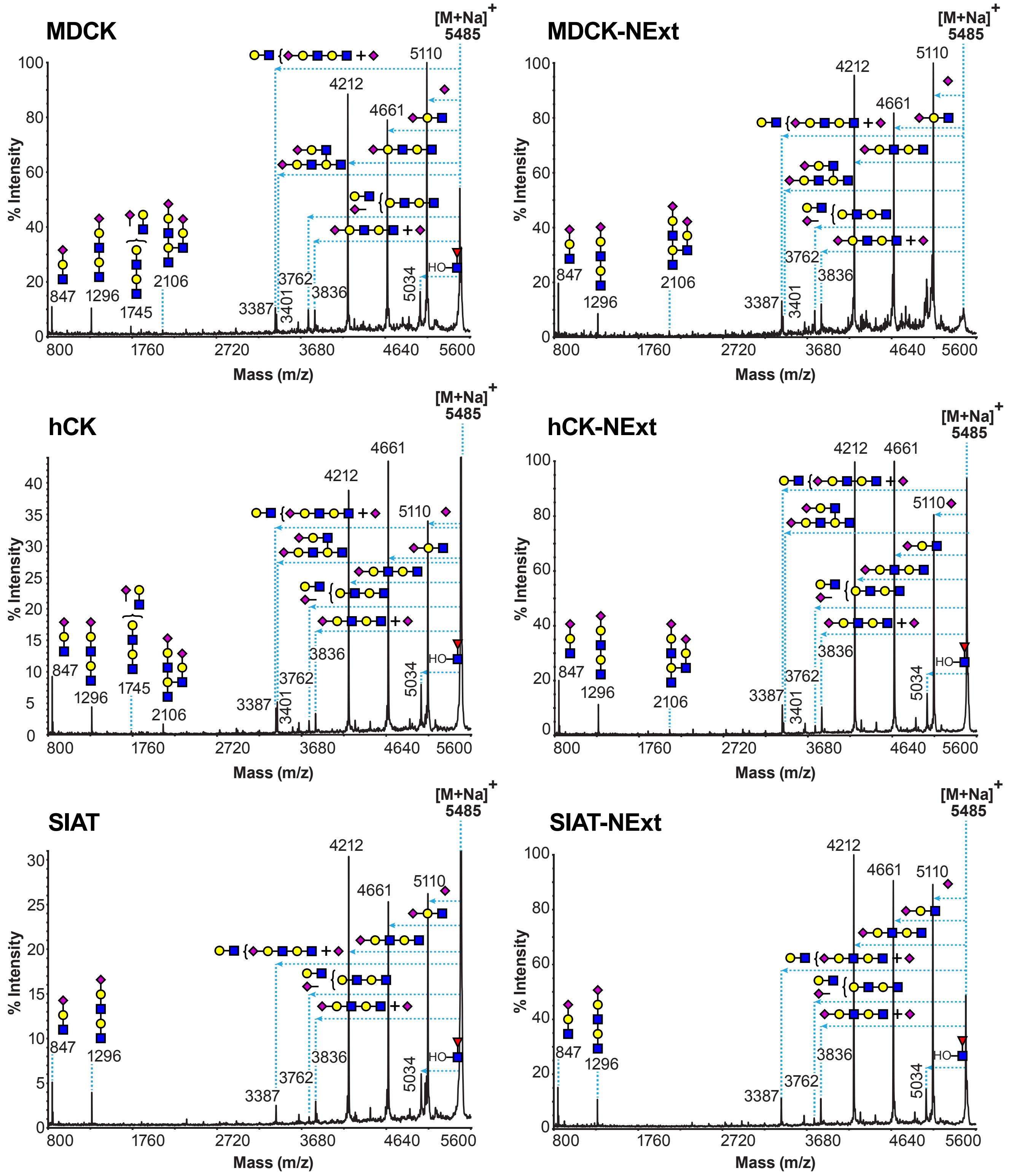
MDCK
MDCK-NExt
hCK
hCK-NExt
SIAT
SIAT-NExt
[M+Na]+
5485
% Intensity
Mass (m/z)
847
1296
1745
2106
3387
3401
3762
3836
4212
4661
5034
5110
HO
800
1760
2720
3680
4640
5600

g

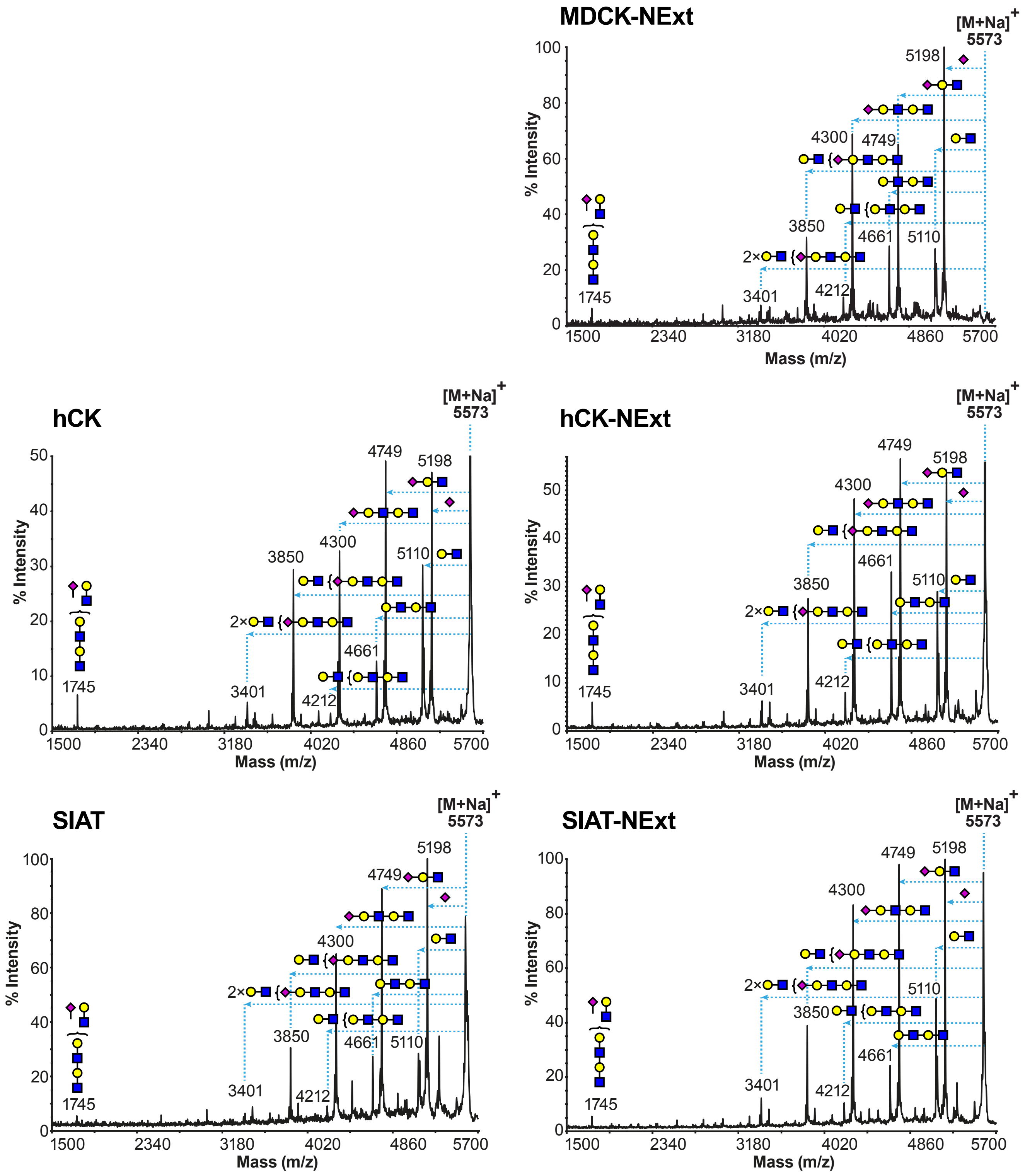

MDCK-NExt
hCK
hCK-NExt
SIAT
SIAT-NExt
[M+Na]+
5573
% Intensity
Mass (m/z)
1500
2340
3180
4020
4860
5700
1745
3401
3850
4212
4300
4661
4749
5110
5198
2×

**h**

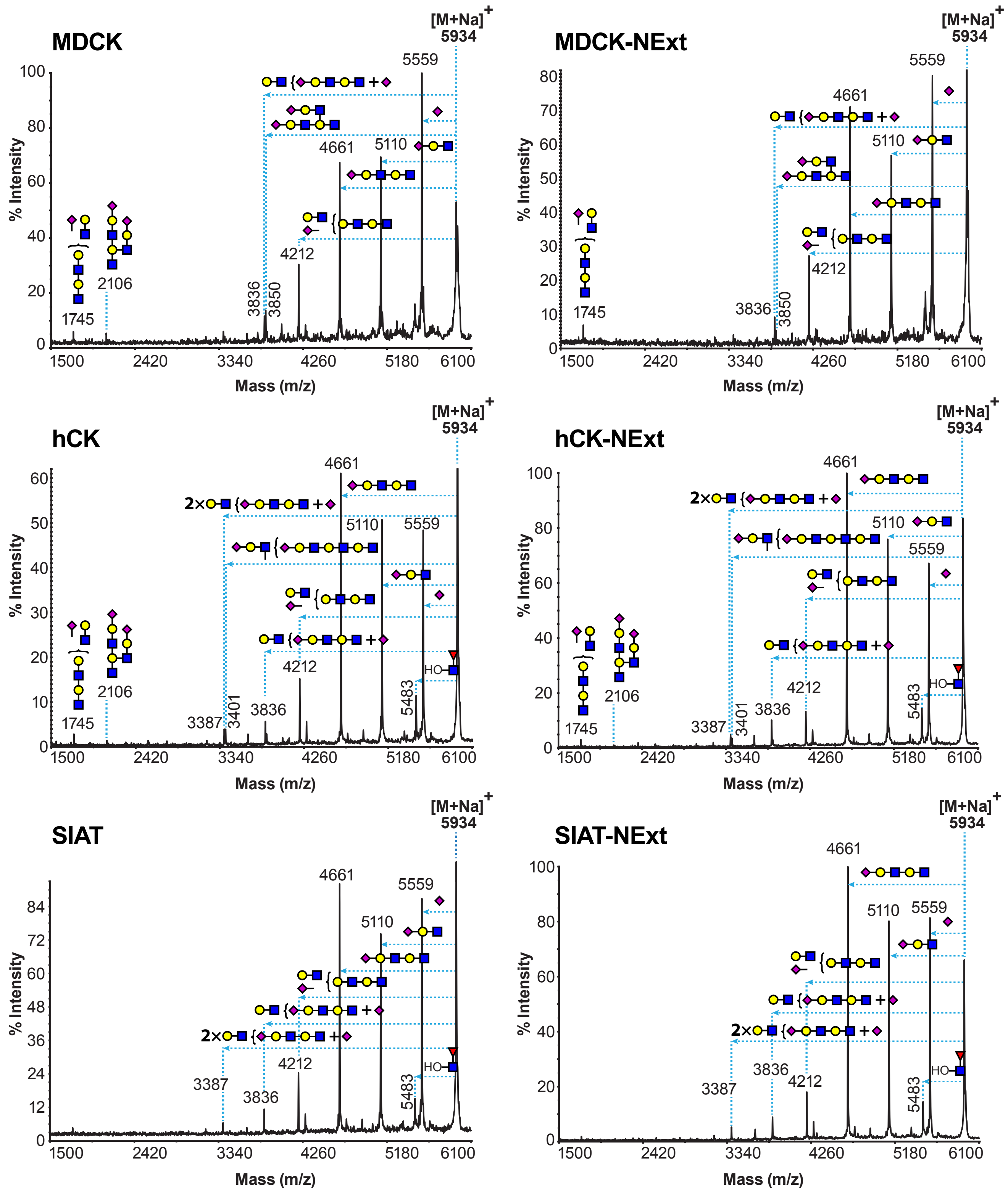
MDCK
MDCK-NExt
hCK
hCK-NExt
SIAT
SIAT-NExt
[M+Na]+
5934
5559
5110
4661
4212
3836
3850
3387
3401
2106
1745
5483
HO
2×
% Intensity
Mass (m/z)
1500
2420
3340
4260
5180
6100

**i**

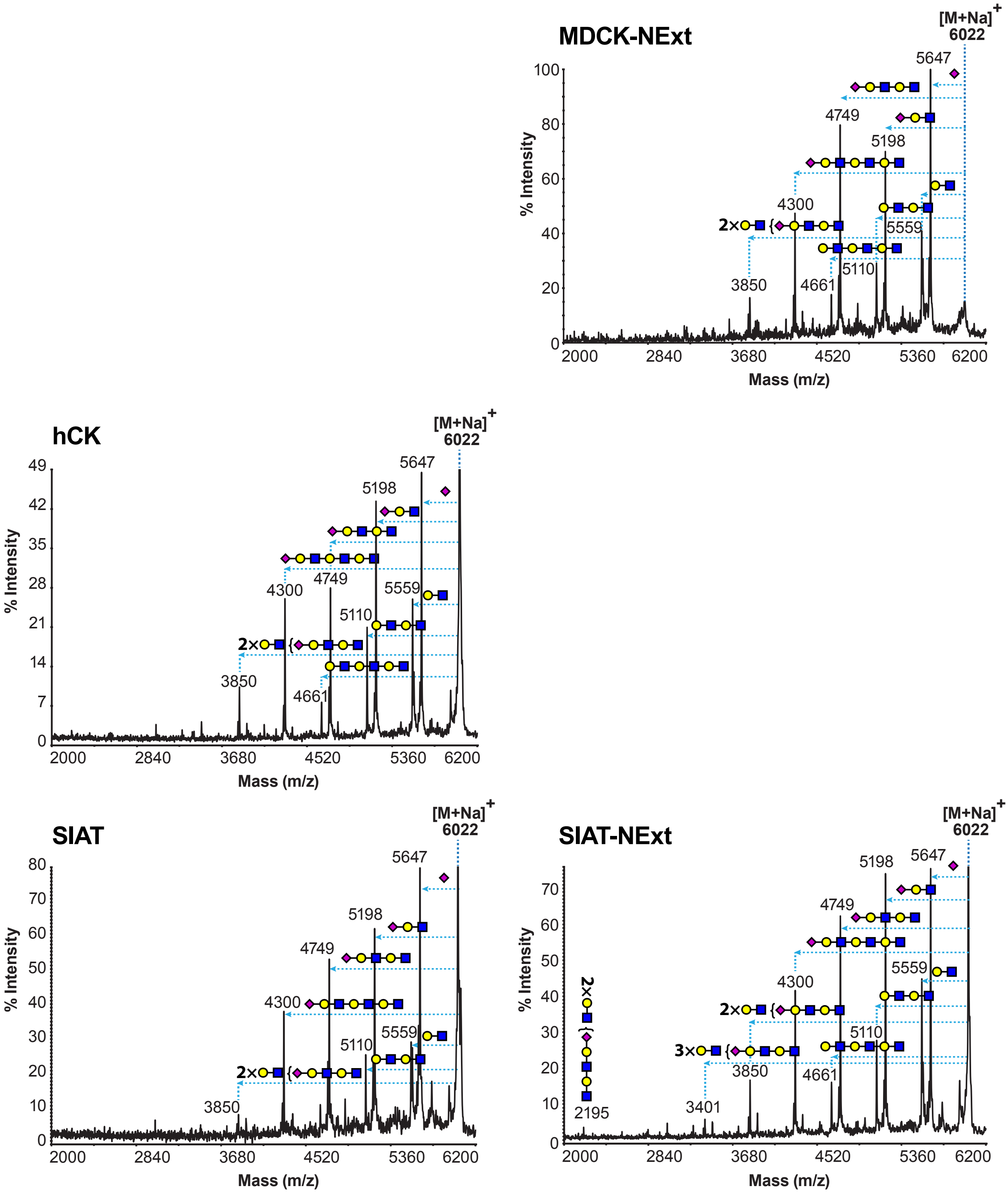
MDCK-NExt
[M+Na]+
6022
5647
4749
5198
4300
5559
3850
4661
5110
2×
% Intensity
Mass (m/z)
hCK
[M+Na]+
6022
5647
5198
4749
4300
5559
5110
2×
3850
4661
% Intensity
Mass (m/z)
SIAT
[M+Na]+
6022
5647
5198
4749
4300
5559
5110
2×
3850
% Intensity
Mass (m/z)
SIAT-NExt
[M+Na]+
6022
5198
5647
4749
4300
5559
2×
5110
3×
3850
4661
2×
2195
3401
% Intensity
Mass (m/z)

j

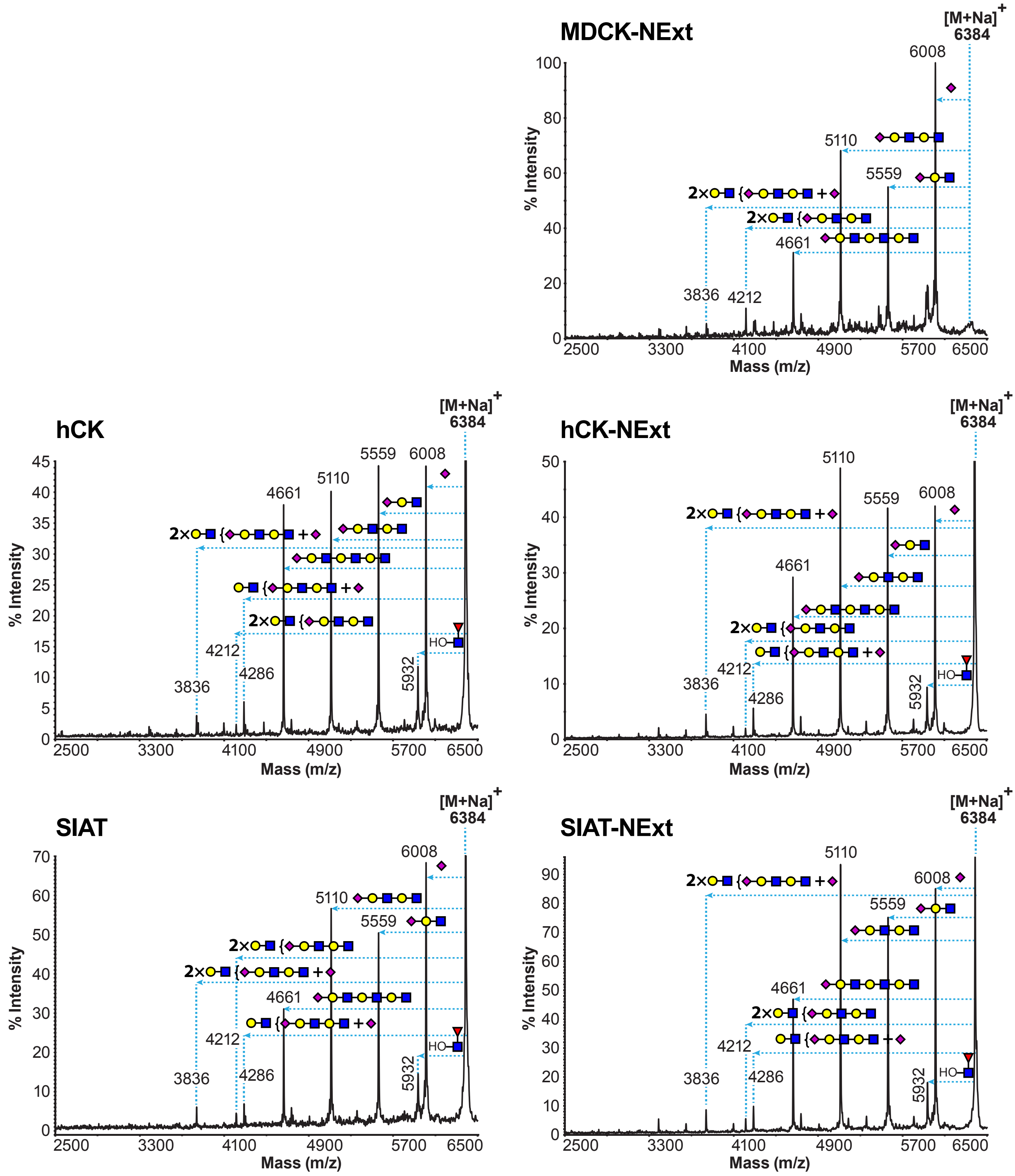
MDCK-NExt
[M+Na]+
6384
6008
5110
5559
4661
3836
4212
2×
% Intensity
Mass (m/z)
2500
3300
4100
4900
5700
6500
hCK
[M+Na]+
6384
5559
6008
5110
4661
4212
4286
3836
5932
HO
hCK-NExt
[M+Na]+
6384
5110
5559
6008
4661
4212
3836
4286
5932
HO
SIAT
[M+Na]+
6384
6008
5110
5559
4661
4212
3836
4286
5932
HO
SIAT-NExt
[M+Na]+
6384
5110
6008
5559
4661
4212
3836
4286
5932
HO

**k**

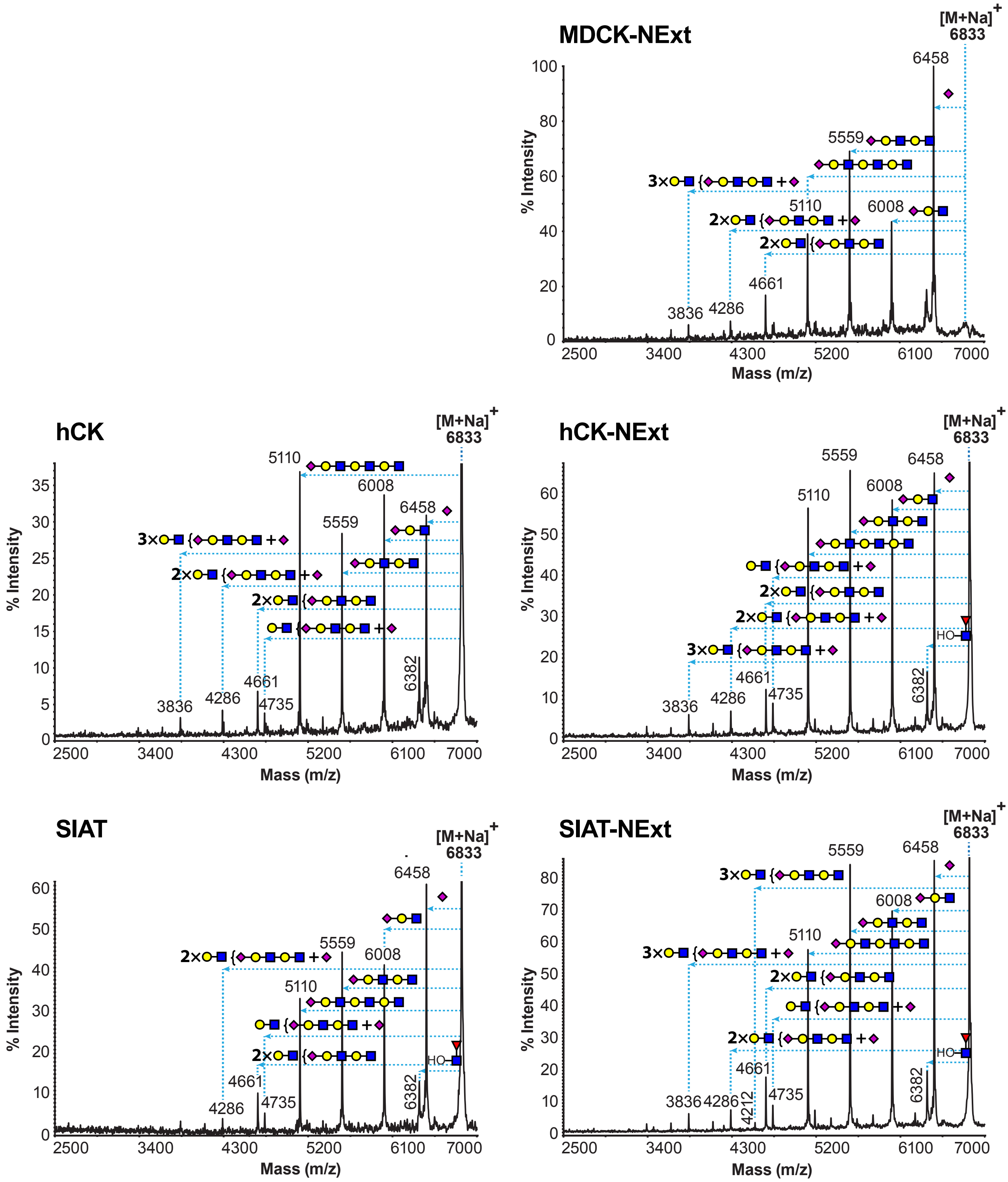
MDCK-NExt
hCK
hCK-NExt
SIAT
SIAT-NExt
[M+Na]+
6833
% Intensity
Mass (m/z)
6458
6008
5559
5110
4735
4661
4286
4212
3836
6382
3×
2×
HO

I

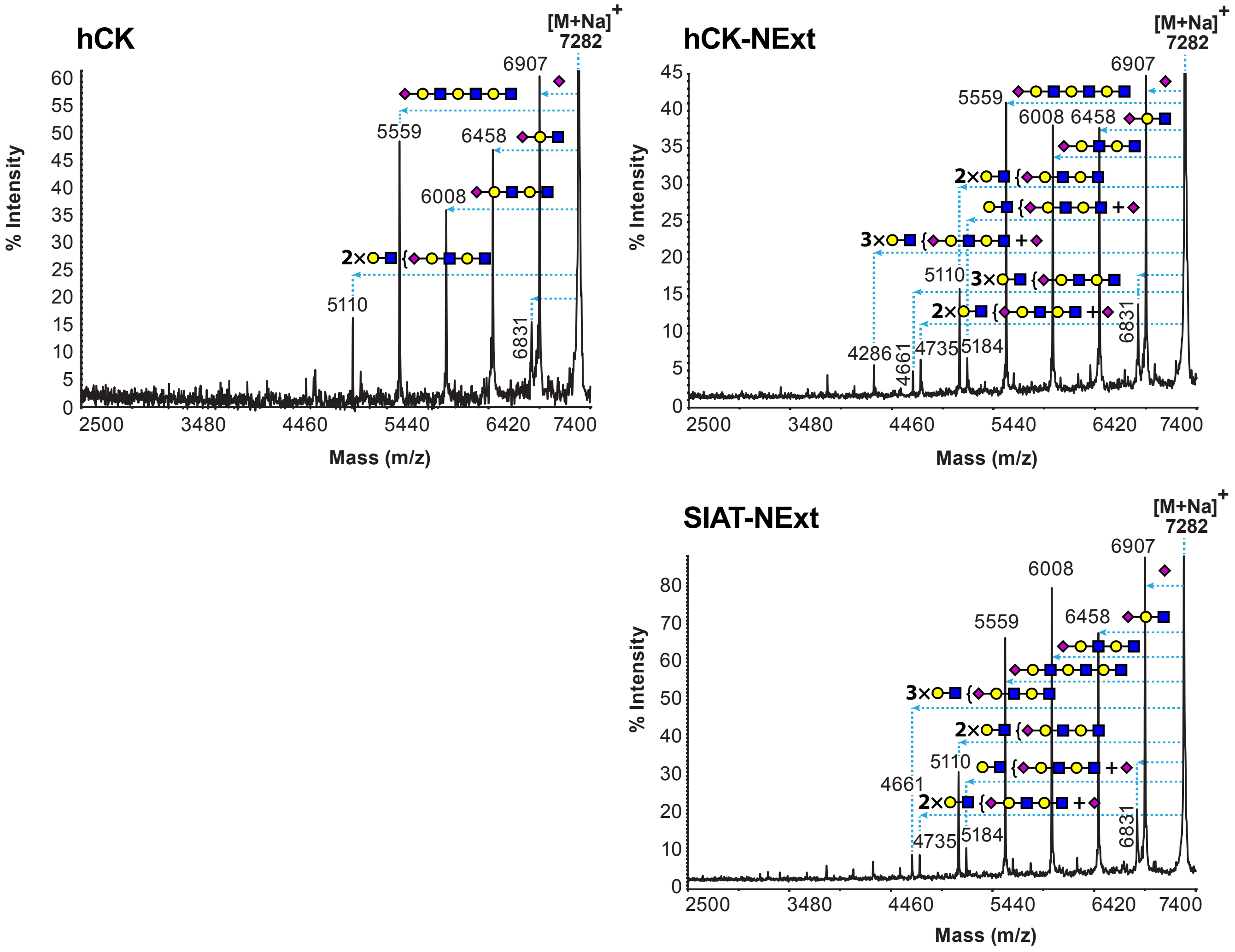
hCK
[M+Na]+
7282
6907
5559
6458
6008
2×
5110
6831
% Intensity
Mass (m/z)
2500
3480
4460
5440
6420
7400
hCK-NExt
4286
4661
4735
5184
SIAT-NExt
3×
+

**m**

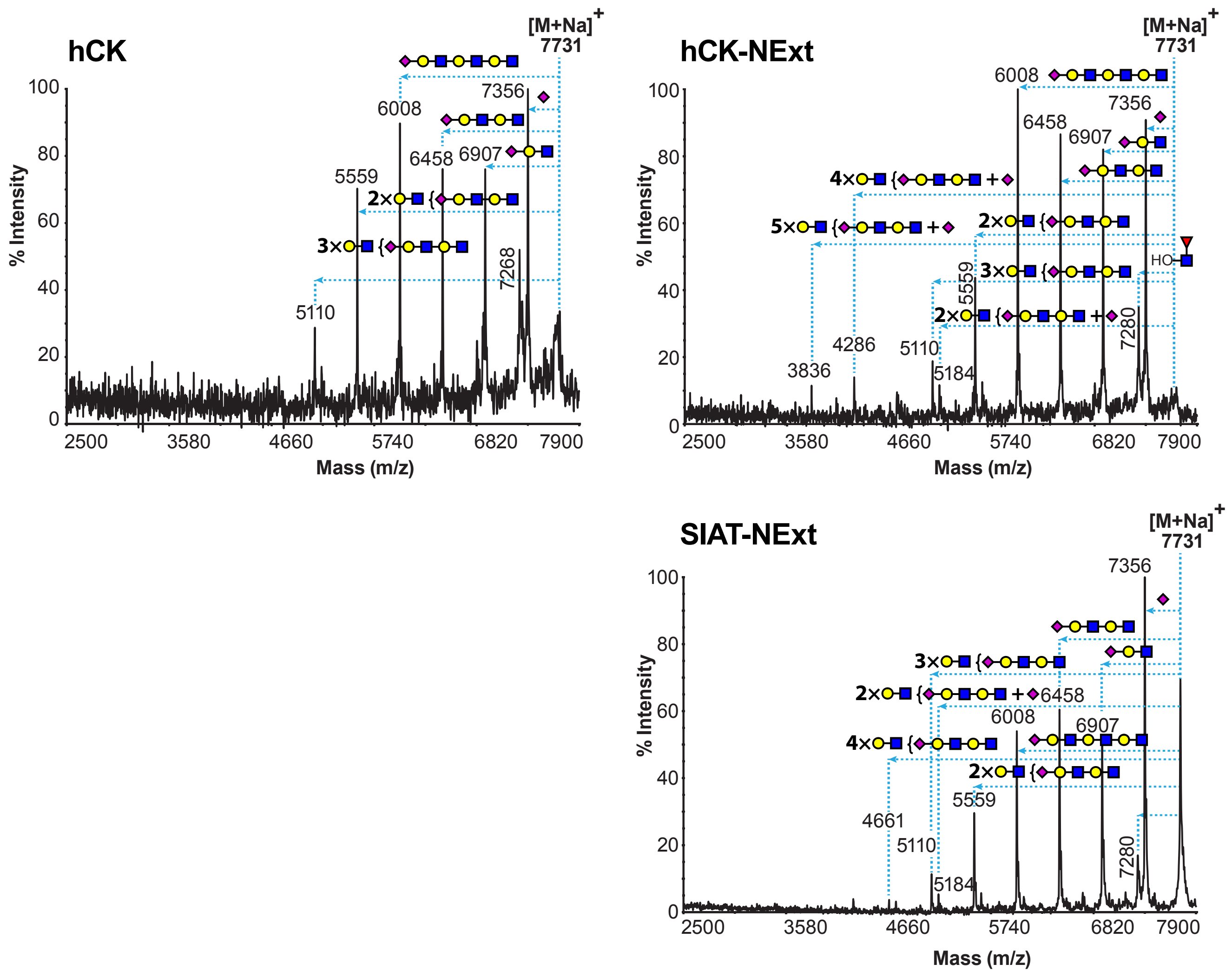

hCK
[M+Na]+
7731
% Intensity
Mass (m/z)
2500
3580
4660
5740
6820
7900
0
20
40
60
80
100
7356
6008
6458
6907
5559
5110
7268
2×
3×
hCK-NExt
[M+Na]+
7731
6008
7356
6458
6907
4×
5×
2×
3×
2×
HO
3836
4286
5110
5184
5559
7280
SIAT-NExt
[M+Na]+
7731
7356
3×
2×
6458
6008
6907
4×
2×
5559
4661
5110
5184
7280

**Supplementary Figure 6. MALDI TOF/TOF MS/MS analysis of parental- and NExt cell-derived N-glycans isolated from MDCK, hCK, and SIAT cell lines.** Molecular ions $[M+Na]^+$ present in **Supplementary Fig. 3a** and **b** (MDCK, and MDCK-NExt respectively), **Supplementary Fig. 7a** and **b** (SIAT and SIAT-NExt respectively) and **Supplementary Fig. 10a** and **b** (hCK and hCK-NExt respectively) were selected for MALDI-TOF/TOF MS/MS analysis. (**a**) m/z 4314, (**b**) m/z 4675, (**c**) m/z 4763, (**d**) m/z 4920, (**e**) m/z 5124, (**f**) m/z 5485, (**g**) m/z 5573, (**h**) m/z 5934, (**i**) m/z 6022, (**j**) m/z 6384, (**k**) m/z 6833, (**l**) m/z 7282 and (**m**) m/z 7731. Horizontal dashed lines correspond to the indicated fragment loss from the corresponding molecular ion $[M+Na]^+$. Vertical dashed lines indicate the m/z fragment ion value that corresponds to the fragment loss as shown from the corresponding horizontal dashed line.

Structures outside the bracket have not unequivocally been defined. For clarity, not all fragments were annotated.

On the MDCKs, the fragment ions corresponding to losses of two (2) or three (3) sialylated LacNAcs from the corresponding molecular ions $[M+Na]^+$ were the most abundant poly-LacNAcs detected (i.e., m/z 5485 at fragment ions m/z 4212 and 3762, m/z 5934 at fragment ions m/z 4661 and 4212). Fragment ions corresponding to losses of four (4) sialylated LacNAcs long were found for molecular ions detected above m/z 6000 and of minor relative abundance (i.e., m/z 6022 at fragment ion m/z 3850, m/z 6384 at 4286, m/z 6833 at 4661). On the MDCKs and hCKs, MALDI TOF/TOF MS/MS analysis of the molecular ion at m/z 5485 (**f**) detected the presence of a fragment ion at m/z 3401 corresponding to a loss of NeuAc2Hex3HexNAc3 from the molecular ion [M+Na]+ accompanied with the detection of the latter fragment at m/z 2106. Both fragment ions (m/z 2106 and 3401) were indicative of I-branched structures. Similarly, for the molecular ion at m/z 5934 (**h;** MDCK panels), the fragment ions at m/z 3850 and 2106 were also indicative of I- branched structures. For the molecular ion at m/z 5934 (**h;** hCK panels), the fragment ion at m/z 3401 was an indication of I-branched N-glycans on poly-LacNAc repeats. Note that the I-branched epitopes found on both MDCKs and hCKs were of very low relative abundance. Note also that on SIATs or SIAT-NExt, for both above molecular ions (m/z 5485 and 5934), no fragments corresponding to I-branched structures were detected. No substantial difference was detected between MDCK and MDCK-NExt cells. No substantial difference was detected between the SIAT and SIAT-NExt cells.

On the SIAT, the fragment ions corresponding to losses of two (2) or three (3) sialylated LacNAcs from the corresponding molecular ions $[M+Na]^+$ were the most abundant poly-LacNAcs detected (i.e., m/z 5485 at fragment ions m/z 4212 and 3762, m/z 5934 at fragment ions m/z 4661 and 4212). Fragment ions corresponding to losses of four (4) sialylated LacNAcs long were found for molecular ions detected above m/z 6000 at of minor relative abundance (i.e., m/z 6022 at fragment ion m/z 3850, m/z 6384 at 4212, m/z 6833 at 4661, m/z 7282 at 5110, m/z 7731 at 5559).

# Supplementary Figure 7a

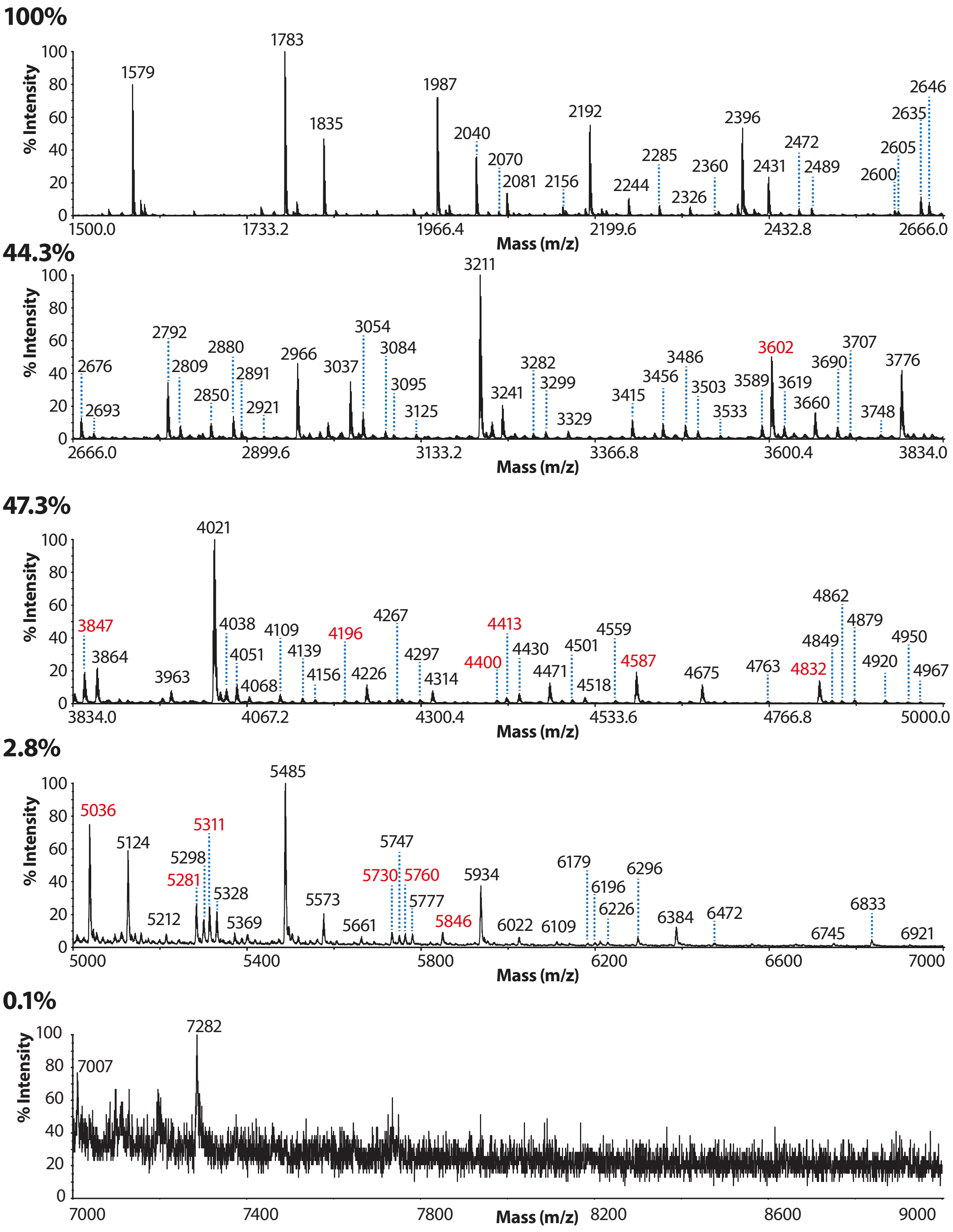

# Supplementary Figure 7b

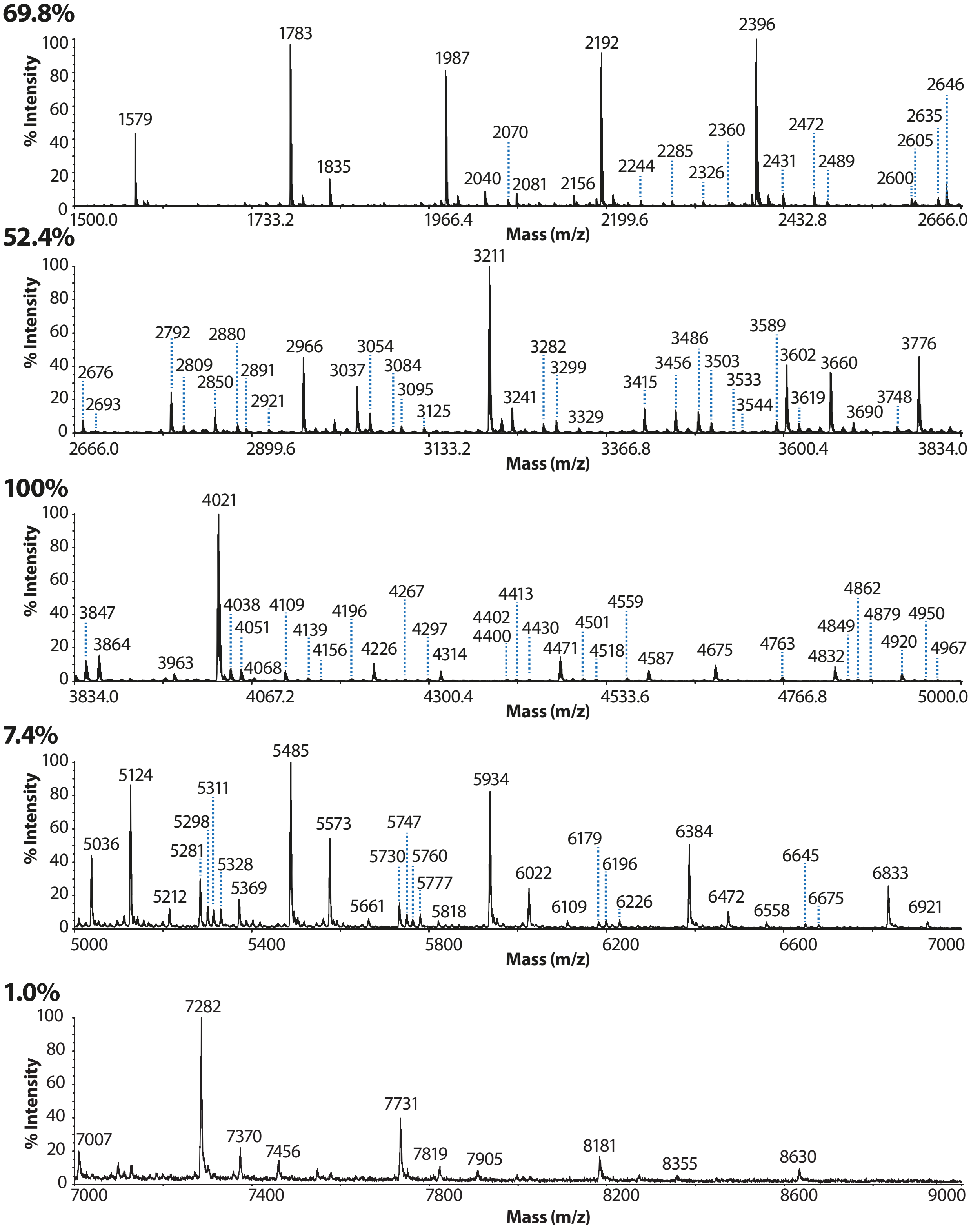

# Supplementary Figure 7c

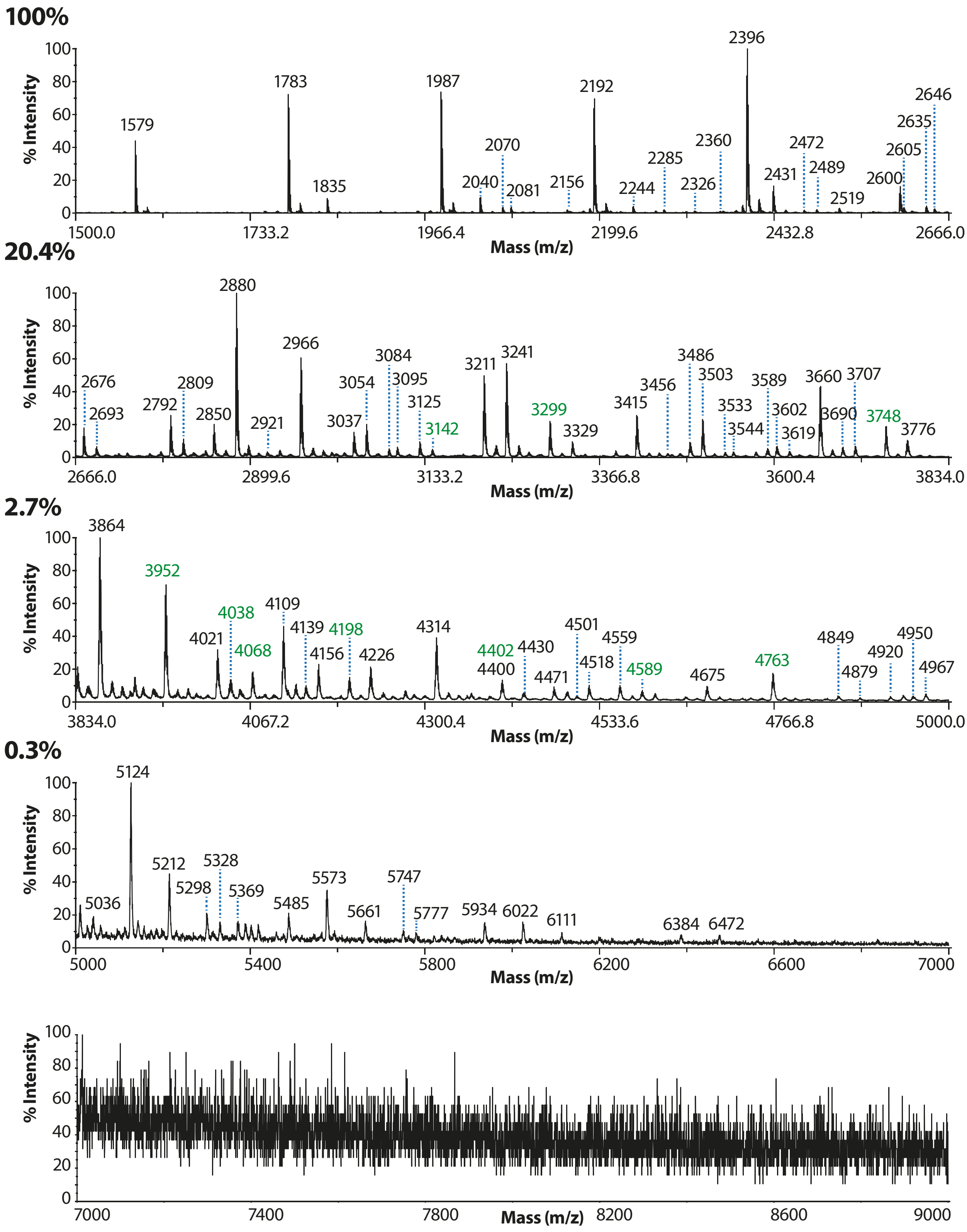

# Supplementary Figure 7d

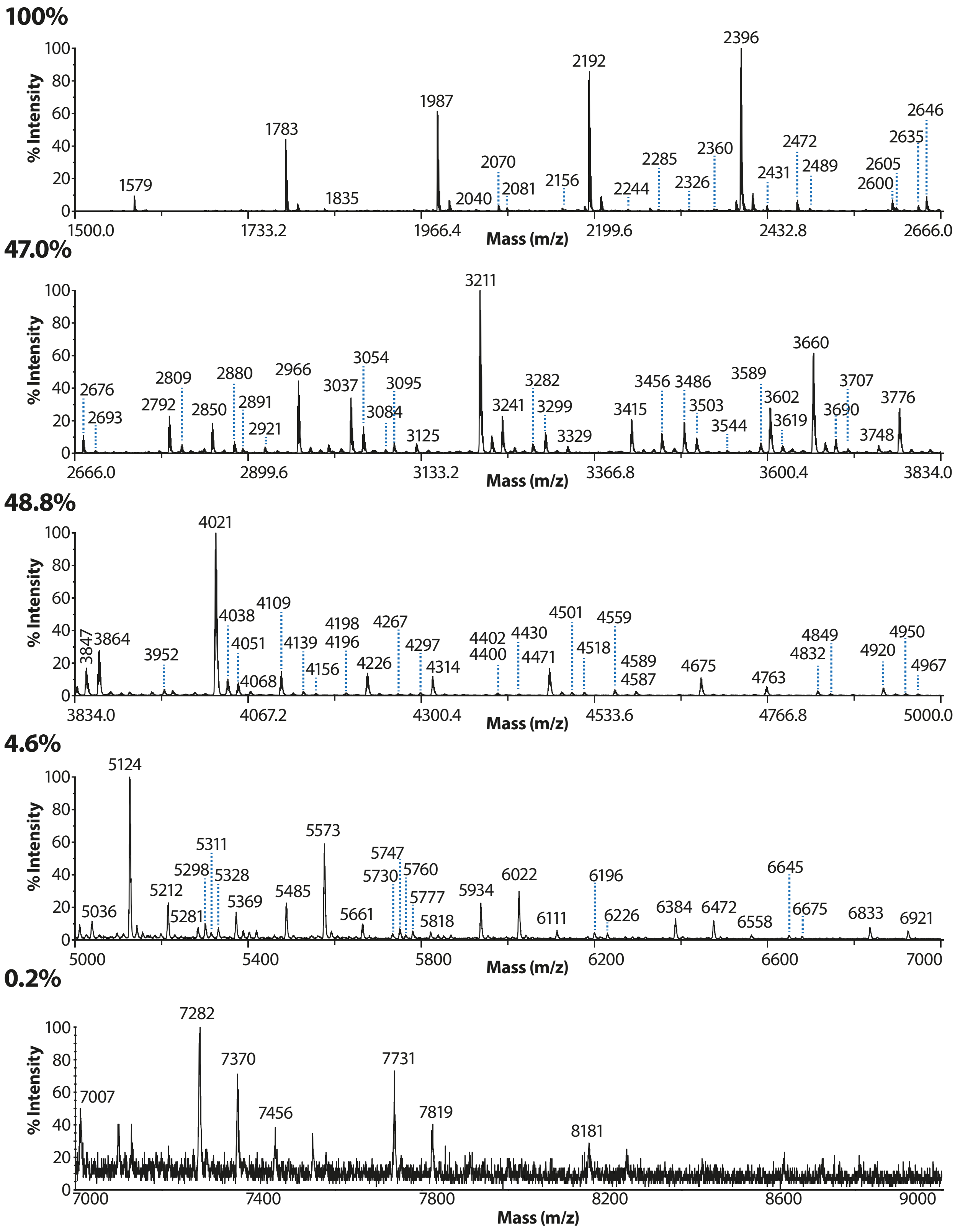

**Supplementary Figure 7. MALDI-TOF MS analysis of permethylated N-glycans from SIAT and SIAT-NExt cells, before or after the Sial-S digestion.** (**a**) SIAT; (**b**) SIAT-NExt; (**c**) SIAT + Sial-S treatment; (**d**) SIAT-NExt + Sial-S treatment. All molecular ions are $[M+Na]^+$. Percentages on top left of each panel correspond to the relative percentage of the maximum peak of the corresponding panel compared to the relative intensity of the maximum peak of the top panel. In (**a**) red values correspond to m/z values of the molecular ions that their relative abundance decreases after the Sial-S digestion, indication of α2-3-linked NeuAc residues. Note that the number of the red peaks here are less than the number of peaks found in the MDCKs (**Supplementary Fig. 3a**) indication that the SIAT1 contain N-glycans with less α2-3-linked NeuAc residues. In (**c**) green peaks correspond to peaks with increased relative abundance after the Sial-S digestion. Full structure annotations can be found in **Supplementary Fig. 8**. MS spectra exported in ASCII format are provided as Source Data file.

# Supplementary Figure 8

## Panel 1

hCK
hCK-NExt
SIAT1
SIAT1-NExt

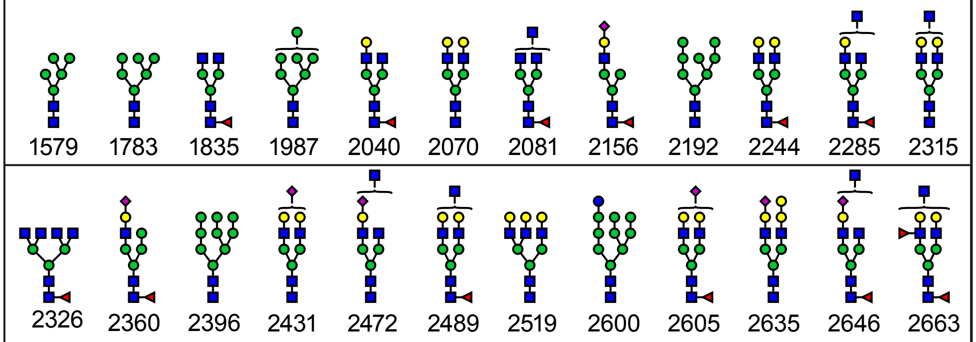


## Panel 2

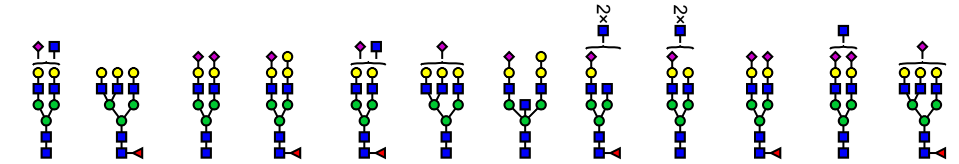

<table>
<tr><th></th><th>2676</th><th>2693</th><th>2792</th><th>2809</th><th>2850</th><th>2880</th><th>2880</th><th>2891</th><th>2921</th><th>2966</th><th>3037</th><th>3054</th></tr>
<tr><td>hCK</td><td>○|○</td><td>−|−</td><td>○|○</td><td>○|○</td><td>○|○</td><td>○|○</td><td>○|○</td><td>○|○</td><td>○|○</td><td>○|○</td><td>●,1|○</td><td>●,1|○</td></tr>
<tr><td>hCK-NExt</td><td>○|○</td><td>−|−</td><td>○|○</td><td>○|○</td><td>○|○</td><td>○|○</td><td>○|○</td><td>○|○</td><td>○|○</td><td>○|○</td><td>●,1|○</td><td>●,1|○</td></tr>
<tr><td>SIAT</td><td>○|○</td><td>○|○</td><td>○|○</td><td>○|○</td><td>○|○</td><td>●,1|●,1</td><td>●,1|●,1</td><td>○|−</td><td>○|○</td><td>○|○</td><td>●,1|○</td><td>●,1|○</td></tr>
<tr><td>SIAT-NExt</td><td>○|○</td><td>○|○</td><td>○|○</td><td>○|○</td><td>○|○</td><td>●,1|○</td><td>●,1|○</td><td>○|○</td><td>○|○</td><td>○|○</td><td>●,1|○</td><td>●,1|○</td></tr>
</table>

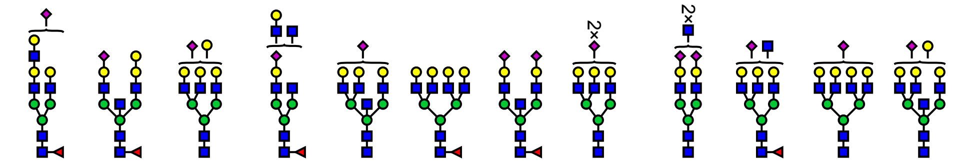

<table>
<tr><th></th><th>3054</th><th>3054</th><th>3084</th><th>3095</th><th>3125</th><th>3142</th><th>3211</th><th>3241</th><th>3282</th><th>3299</th><th>3329</th><th>3329</th></tr>
<tr><td>hCK</td><td>●,2|○</td><td>●,1|○</td><td>○|○</td><td>○|○</td><td>○|○</td><td>−|−</td><td>○|○</td><td>○|○</td><td>○|○</td><td>○|○</td><td>○|○</td><td>○|○</td></tr>
<tr><td>hCK-NExt</td><td>●,2|○</td><td>●,1|○</td><td>○|○</td><td>○|○</td><td>○|○</td><td>−|−</td><td>○|○</td><td>○|○</td><td>○|○</td><td>○|○</td><td>○|○</td><td>○|○</td></tr>
<tr><td>SIAT</td><td>●,2|○</td><td>●,1|○</td><td>○|○</td><td>○|○</td><td>○|○</td><td>−|○</td><td>○|○</td><td>○|○</td><td>○|−</td><td>○|●,1</td><td>○|○</td><td>○|○</td></tr>
<tr><td>SIAT-NExt</td><td>●,2|○</td><td>●,1|○</td><td>○|○</td><td>○|●,2</td><td>○|○</td><td>−|−</td><td>○|○</td><td>○|○</td><td>○|○</td><td>○|●,2</td><td>○|○</td><td>○|○</td></tr>
</table>

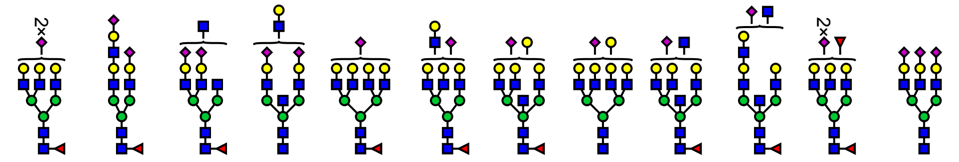

<table>
<tr><th></th><th>3415</th><th>3415</th><th>3456</th><th>3486</th><th>3503</th><th>3503</th><th>3503</th><th>3533</th><th>3544</th><th>3544</th><th>3589</th><th>3602</th></tr>
<tr><td>hCK</td><td>●|●</td><td>●,2|●</td><td>○|●</td><td>○|○</td><td>●|●</td><td>●,2|●</td><td>●|●</td><td>○|○</td><td>○|●</td><td>○|●</td><td>○|○</td><td>○|○</td></tr>
<tr><td>hCK-NExt</td><td>●|●</td><td>●,2|●,2</td><td>○|●</td><td>○|●,2</td><td>●|●</td><td>●,2|●,1</td><td>●|●</td><td>○|○</td><td>○|○</td><td>○|○</td><td>○|○</td><td>○|○</td></tr>
<tr><td>SIAT</td><td>○|●</td><td>○|●,2</td><td>○|○</td><td>○|●,2</td><td>●|●</td><td>●,1|●,1</td><td>●|●</td><td>○|○</td><td>−|○</td><td>−|○</td><td>○|○</td><td>○|○</td></tr>
<tr><td>SIAT-NExt</td><td>○|●</td><td>○|●,2</td><td>○|●</td><td>○|●,2</td><td>●|●</td><td>●,2|●,2</td><td>●|●</td><td>○|−</td><td>○|○</td><td>○|○</td><td>○|○</td><td>○|○</td></tr>
</table>

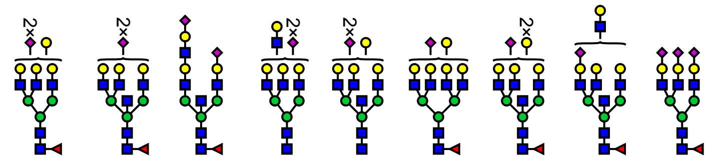

<table>
<tr><th></th><th>3619</th><th>3660</th><th>3660</th><th>3690</th><th>3690</th><th>3707</th><th>3707</th><th>3748</th><th>3776</th></tr>
<tr><td>hCK</td><td>○|○</td><td>○|●</td><td>○|●</td><td>○|○</td><td>○|○</td><td>○|○</td><td>○|○</td><td>○|○</td><td>○|○</td></tr>
<tr><td>hCK-NExt</td><td>○|○</td><td>○|●</td><td>○|●,2</td><td>○|●,2</td><td>○|●,2</td><td>○|○</td><td>○|○</td><td>○|○</td><td>○|○</td></tr>
<tr><td>SIAT</td><td>○|○</td><td>○|●</td><td>○|●,2</td><td>○|○</td><td>○|○</td><td>○|○</td><td>○|○</td><td>○|●,1</td><td>○|○</td></tr>
<tr><td>SIAT-NExt</td><td>○|○</td><td>○|●</td><td>○|●,2</td><td>○|○</td><td>○|○</td><td>−|○</td><td>−|○</td><td>○|○</td><td>○|○</td></tr>
</table>

Number (*n*)=◆[○■]$_n$ Normal | Sial-S; ○ = Detected (not MS/MS); ● = MS/MS; − = not detected

## Panel 3

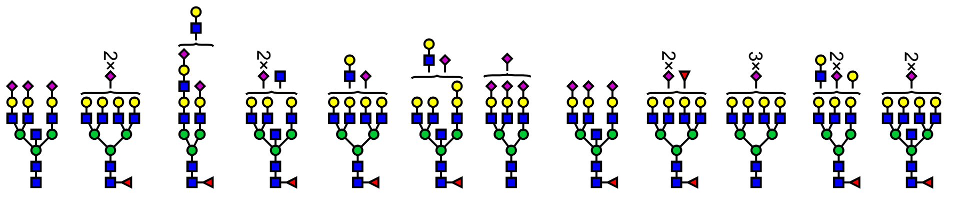

<table>
<tr><th></th><th>3847</th><th>3864</th><th>3864</th><th>3905</th><th>3952</th><th>3952</th><th>3963</th><th>4021</th><th>4038</th><th>4051</th><th>4068</th><th>4109</th></tr>
<tr><td>hCK</td><td>○|○</td><td>●|●</td><td>●,2|●</td><td>○|○</td><td>○|○</td><td>○|○</td><td>−|−</td><td>●|●</td><td>○|○</td><td>○|○</td><td>○|●,2</td><td>●|●</td></tr>
<tr><td>hCK-NExt</td><td>○|○</td><td>●|●</td><td>●,3|●,3</td><td>○|○</td><td>○|○</td><td>○|○</td><td>−|−</td><td>●|●</td><td>○|○</td><td>○|○</td><td>○|○</td><td>○|○</td></tr>
<tr><td>SIAT</td><td>○|−</td><td>●|○</td><td>●,3|○</td><td>−|−</td><td>−|●,2</td><td>−|●,2</td><td>○|−</td><td>○|●</td><td>○|○</td><td>○|−</td><td>○|○</td><td>●|●</td></tr>
<tr><td>SIAT-NExt</td><td>○|○</td><td>●|●</td><td>●,3|●,3</td><td>−|−</td><td>−|○</td><td>−|○</td><td>○|−</td><td>○|●</td><td>○|○</td><td>○|○</td><td>○|○</td><td>●|●</td></tr>
</table>

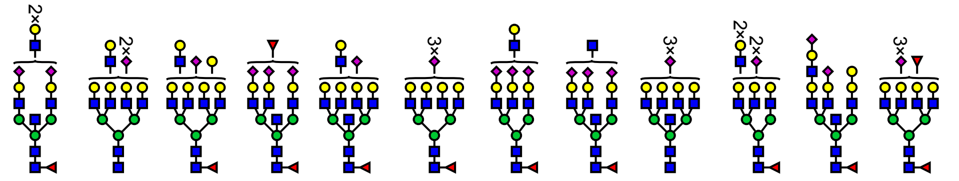

<table>
<tr><th></th><th>4109</th><th>4139</th><th>4156</th><th>4196</th><th>4198</th><th>4226</th><th>4226</th><th>4267</th><th>4297</th><th>4314</th><th>4314</th><th>4400</th></tr>
<tr><td>hCK</td><td>●,2|●,3</td><td>●,2|○</td><td>●,1|−</td><td>−|−</td><td>−|−</td><td>○|●</td><td>○|●,2</td><td>○|−</td><td>−|−</td><td>●,3|●,2</td><td>●|●</td><td>○|●</td></tr>
<tr><td>hCK-NExt</td><td>○|○</td><td>●,2|○</td><td>●,1|○</td><td>−|−</td><td>−|−</td><td>○|●</td><td>○|●,2</td><td>○|−</td><td>−|−</td><td>●,4|●,3</td><td>●|●</td><td>○|○</td></tr>
<tr><td>SIAT</td><td>●,3|●,2</td><td>○|○</td><td>○|○</td><td>○|−</td><td>−|○</td><td>○|○</td><td>○|○</td><td>○|−</td><td>○|−</td><td>●,2|○</td><td>●|○</td><td>○|●</td></tr>
<tr><td>SIAT-NExt</td><td>●,3|●,3</td><td>○|○</td><td>○|○</td><td>○|○</td><td>−|○</td><td>●|●</td><td>●,2|●,2</td><td>○|○</td><td>○|○</td><td>●,4|●,3</td><td>●|●</td><td>○|○</td></tr>
</table>

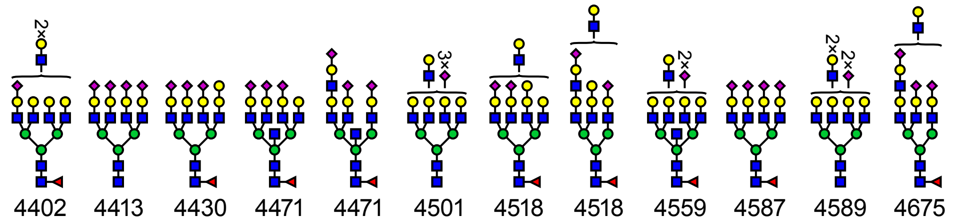

<table>
<tr><th></th><th>4402</th><th>4413</th><th>4430</th><th>4471</th><th>4471</th><th>4501</th><th>4518</th><th>4518</th><th>4559</th><th>4587</th><th>4589</th><th>4675</th></tr>
<tr><td>hCK</td><td>−|−</td><td>○|●</td><td>●,1|●</td><td>○|○</td><td>○|○</td><td>○|○</td><td>○|○</td><td>○|○</td><td>○|○</td><td>○|○</td><td>−|−</td><td>●,3|●,3</td></tr>
<tr><td>hCK-NExt</td><td>−|−</td><td>○|○</td><td>○|●</td><td>●|●</td><td>●,2|●,2</td><td>○|○</td><td>○|●</td><td>○|●,3</td><td>○|○</td><td>○|○</td><td>−|−</td><td>●,3|●,3</td></tr>
<tr><td>SIAT</td><td>−|●,3</td><td>○|−</td><td>○|○</td><td>○|○</td><td>○|○</td><td>○|○</td><td>○|○</td><td>○|○</td><td>○|○</td><td>○|−</td><td>−|●,2</td><td>●,3|●,2</td></tr>
<tr><td>SIAT-NExt</td><td>○|○</td><td>○|−</td><td>○|○</td><td>●|●</td><td>●,2|●,2</td><td>○|○</td><td>○|○</td><td>○|○</td><td>○|○</td><td>○|○</td><td>−|○</td><td>●,3|●,3</td></tr>
</table>

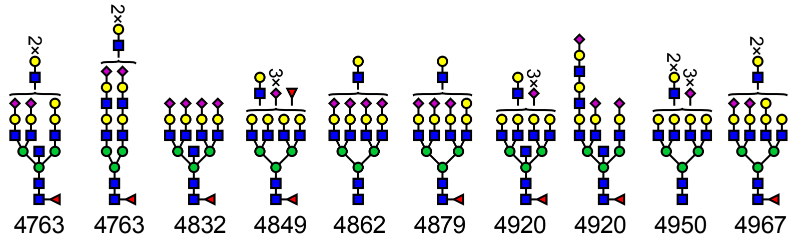

<table>
<tr><th></th><th>4763</th><th>4763</th><th>4832</th><th>4849</th><th>4862</th><th>4879</th><th>4920</th><th>4920</th><th>4950</th><th>4967</th></tr>
<tr><td>hCK</td><td>●,3|○</td><td>●,4|○</td><td>○|−</td><td>○|○</td><td>−|−</td><td>○|○</td><td>●,2|●,2</td><td>●,3|●,3</td><td>○|○</td><td>−|−</td></tr>
<tr><td>hCK-NExt</td><td>●,3|●,3</td><td>●,4|●,3</td><td>○|−</td><td>○|○</td><td>−|−</td><td>○|○</td><td>●,2|●,2</td><td>●,3|●,3</td><td>○|○</td><td>○|○</td></tr>
<tr><td>SIAT</td><td>●,3|●</td><td>●,4|●,4</td><td>○|−</td><td>○|○</td><td>○|−</td><td>○|○</td><td>●,2|○</td><td>●,3|○</td><td>○|○</td><td>○|○</td></tr>
<tr><td>SIAT-NExt</td><td>●|●</td><td>●,4|●,4</td><td>○|●</td><td>○|○</td><td>○|−</td><td>○|−</td><td>●,2|●,2</td><td>●,3|●,3</td><td>○|○</td><td>○|○</td></tr>
</table>

Number (*n*)=◆[○-■]$_n$ Normal | Sial-S; ○ = Detected (not MS/MS); ● = MS/MS; − = not detected

## Panel 4

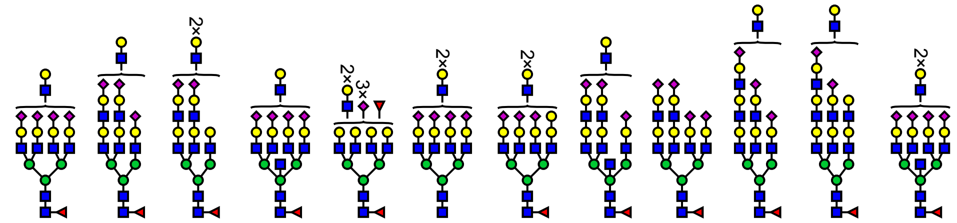

<table>
<tr><th></th><th>5036</th><th>5124</th><th>5212</th><th>5281</th><th>5298</th><th>5311</th><th>5328</th><th>5369</th><th>5485</th><th>5573</th><th>5661</th><th>5730</th></tr>
<tr><td>hCK</td><td>○|○</td><td>●,3|●,3</td><td>●,4|○</td><td>−|−</td><td>○|○</td><td>○|○</td><td>○|○</td><td>○|●,3</td><td>●,3|●,2</td><td>●,4|●,3</td><td>−|−</td><td>○|○</td></tr>
<tr><td>hCK-NExt</td><td>●,2|○</td><td>●,3|●,3</td><td>●,4|○</td><td>○|−</td><td>○|○</td><td>○|○</td><td>○|○</td><td>○|○</td><td>●,3|●,2</td><td>●,4|●,4</td><td>−|−</td><td>○|○</td></tr>
<tr><td>SIAT</td><td>○|○</td><td>●,3|●,3</td><td>●,4|●,3</td><td>●,2|−</td><td>○|○</td><td>○|−</td><td>○|●</td><td>○|○</td><td>●,3|●,2</td><td>●,4|●,3</td><td>○|○</td><td>○|−</td></tr>
<tr><td>SIAT-NExt</td><td>○|○</td><td>●,4|●,3</td><td>●,4|●,4</td><td>●,2|○</td><td>○|○</td><td>○|○</td><td>○|●</td><td>○|●,3</td><td>●,3|●,2</td><td>●,4|●,4</td><td>○|●,4</td><td>○|○</td></tr>
</table>

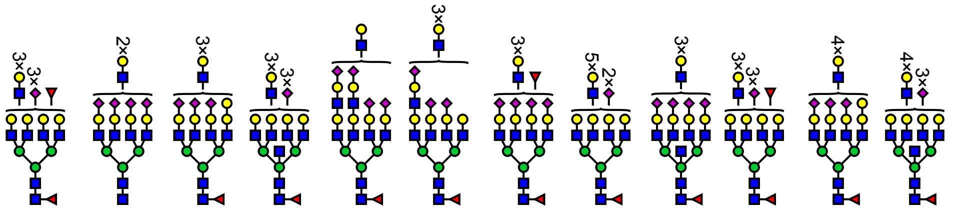

<table>
<tr><th></th><th>5747</th><th>5760</th><th>5777</th><th>5818</th><th>5934</th><th>6022</th><th>6109</th><th>6111</th><th>6179</th><th>6196</th><th>6226</th><th>6267</th></tr>
<tr><td>hCK</td><td>○|○</td><td>○|○</td><td>○|○</td><td>○|○</td><td>●,3|●,3</td><td>●,4|●,4</td><td>○|○</td><td>−|−</td><td>○|○</td><td>○|○</td><td>○|○</td><td>○|○</td></tr>
<tr><td>hCK-NExt</td><td>○|○</td><td>○|○</td><td>○|○</td><td>−|−</td><td>●,3|●,3</td><td>○|○</td><td>○|○</td><td>−|−</td><td>○|○</td><td>○|○</td><td>○|○</td><td>−|−</td></tr>
<tr><td>SIAT</td><td>○|○</td><td>○|−</td><td>○|○</td><td>−|−</td><td>●,3|●,2</td><td>●,4|●,4</td><td>○|−</td><td>−|●,4</td><td>○|−</td><td>○|−</td><td>○|−</td><td>−|−</td></tr>
<tr><td>SIAT-NExt</td><td>○|○</td><td>○|○</td><td>○|○</td><td>○|○</td><td>●,3|●,3</td><td>●,4|●,4</td><td>○|−</td><td>−|●,5</td><td>○|−</td><td>○|○</td><td>○|○</td><td>−|−</td></tr>
</table>

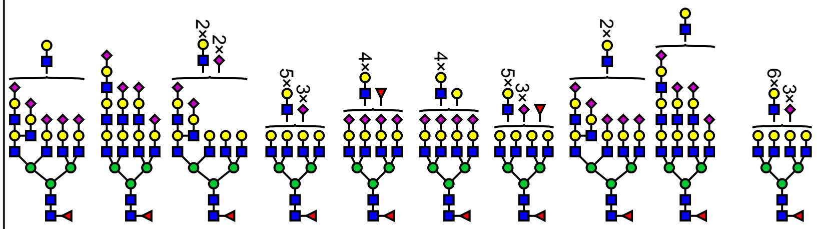

<table>
<tr><th></th><th>6296</th><th>6384</th><th>6384</th><th>6472</th><th>6558</th><th>6588</th><th>6645</th><th>6745</th><th>6833</th><th>6921</th></tr>
<tr><td>hCK</td><td>○|−</td><td>●,4|●,3</td><td>●|−</td><td>○|●,5</td><td>○|○</td><td>−|−</td><td>−|−</td><td>○|−</td><td>●,4|●,4</td><td>○|●,5</td></tr>
<tr><td>hCK-NExt</td><td>○|−</td><td>●,4|●,3</td><td>●|−</td><td>○|●,5</td><td>○|○</td><td>○|○</td><td>−|−</td><td>○|−</td><td>●,4|●,4</td><td>○|○</td></tr>
<tr><td>SIAT</td><td>○|−</td><td>●,4|○</td><td>−|−</td><td>○|●,4</td><td>−|−</td><td>−|−</td><td>−|−</td><td>○|−</td><td>●,4|−</td><td>○|−</td></tr>
<tr><td>SIAT-NExt</td><td>−|−</td><td>●,4|●,3</td><td>−|−</td><td>●,5|●,5</td><td>○|○</td><td>−|−</td><td>○|○</td><td>−|−</td><td>●,5|●,3</td><td>○|●,5</td></tr>
</table>

Number ($n$)=◆[○-■]$_n$ Normal | Sial-S; ○ = Detected (not MS/MS); ● = MS/MS; − = not detected

## Panel 5

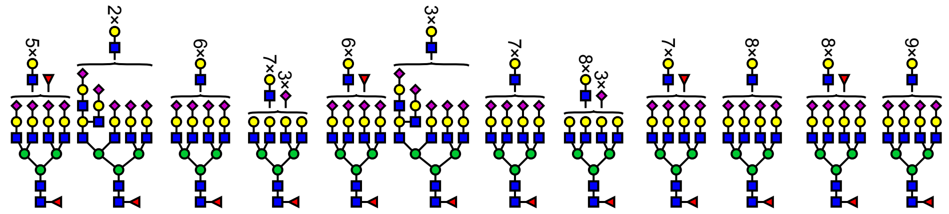

<table>
<tr><th></th><th>7007</th><th>7194</th><th>7282</th><th>7370</th><th>7456</th><th>7643</th><th>7731</th><th>7819</th><th>7905</th><th>8181</th><th>8355</th><th>8630</th></tr>
<tr><td>hCK</td><td>○|○</td><td>○|−</td><td>●,4|●,4</td><td>○|−</td><td>○|−</td><td>○|−</td><td>●,5|○</td><td>−|−</td><td>−|−</td><td>−|−</td><td>−|−</td><td>−|−</td></tr>
<tr><td>hCK-NExt</td><td>○|○</td><td>○|−</td><td>●,5|●,3</td><td>○|−</td><td>○|−</td><td>○|−</td><td>●,5|○</td><td>○|−</td><td>−|−</td><td>○|−</td><td>−|−</td><td>−|−</td></tr>
<tr><td>SIAT</td><td>○|−</td><td>−|−</td><td>○|−</td><td>−|−</td><td>−|−</td><td>−|−</td><td>−|−</td><td>−|−</td><td>−|−</td><td>−|−</td><td>−|−</td><td>−|−</td></tr>
<tr><td>SIAT-NExt</td><td>○|○</td><td>−|−</td><td>●,5|●,3</td><td>○|○</td><td>○|○</td><td>−|−</td><td>●,6|○</td><td>○|○</td><td>○|−</td><td>●,5|●,5</td><td>○|−</td><td>●,6|−</td></tr>
</table>

Number ($n$)=◆[○-■]$_n$ Normal | Sial-S; ○ = Detected (not MS/MS); ● = MS/MS; − = not detected

**Supplementary Figure 8. Structures of N-glycans found on parental-and NExt-cells from hCK and SIAT cells, before or after the Sial-S digestion.** Putative structures are based on composition, biosynthetic knowledge and tandem mass spectrometry where available. Cartoon structures were drawn according to http://www.functionalglycomics.org guidelines. Structures are organized into panels according to the presentation of the mass spectra found in the corresponding Supplementary Figures. Value under each putative structure corresponds to the molecular ion [M+Na]+ m/z value detected in the corresponding **Supplementary Fig. 7** for SIATs (**a**, SIAT; **b**, SIAT-NExt; **c**, SIAT + Sial-S treatment; **d**, SIAT-NExt + Sial-S treatment) or **Supplementary Fig. 10** for hCKs (**a**, hCK; **b**, hCK-NExt; **c**, hCK + Sial-S treatment; **d**, hCK-NExt + Sial-S treatment). Dash (" ‾ ") corresponds to non-detected structure, open circle ("○") corresponds to detected structure, but not been subjected to MALDI-TOF/TOF MS/MS analysis, solid circle ("●") corresponds to a detected structure subjected to MALDI-TOF/TOF MS/MS analysis. The symbols before the vertical line, indicate the detection and/or MS/MS status for the parental- or NExt-cells-derived N-glycans, while the symbols after the vertical line, indicate the detected and/or MS/MS status for the parental + Sial-S or NExt + Sial-S N-glycans. Number after the solid circle corresponds to the maximum LacNAc repeats with a terminal NeuAc residue. When this number is found on Sial-S digested samples, this number corresponds to α2-6-linked NeuAc residues. Structures above the bracket have not unequivocally been defined. MALDI TOF/TOF MS/MS analysis on selected molecular ions before or after Sial-S digestion can be found on the **Supplementary Fig. 6** and **9** respectively (hCKs and SIATs panels). Structures above the bracket have not unequivocally been defined. Panel 1 range, m/z 1500 to 2666; Panel 2 range, m/z 2666 to 3834; Panel 3 range, m/z 3834 to 5000; Panel 4 range, m/z 5000 to 7000; and panel 5 range, m/z 7000 to 9000.

# Supplementary Figure 9

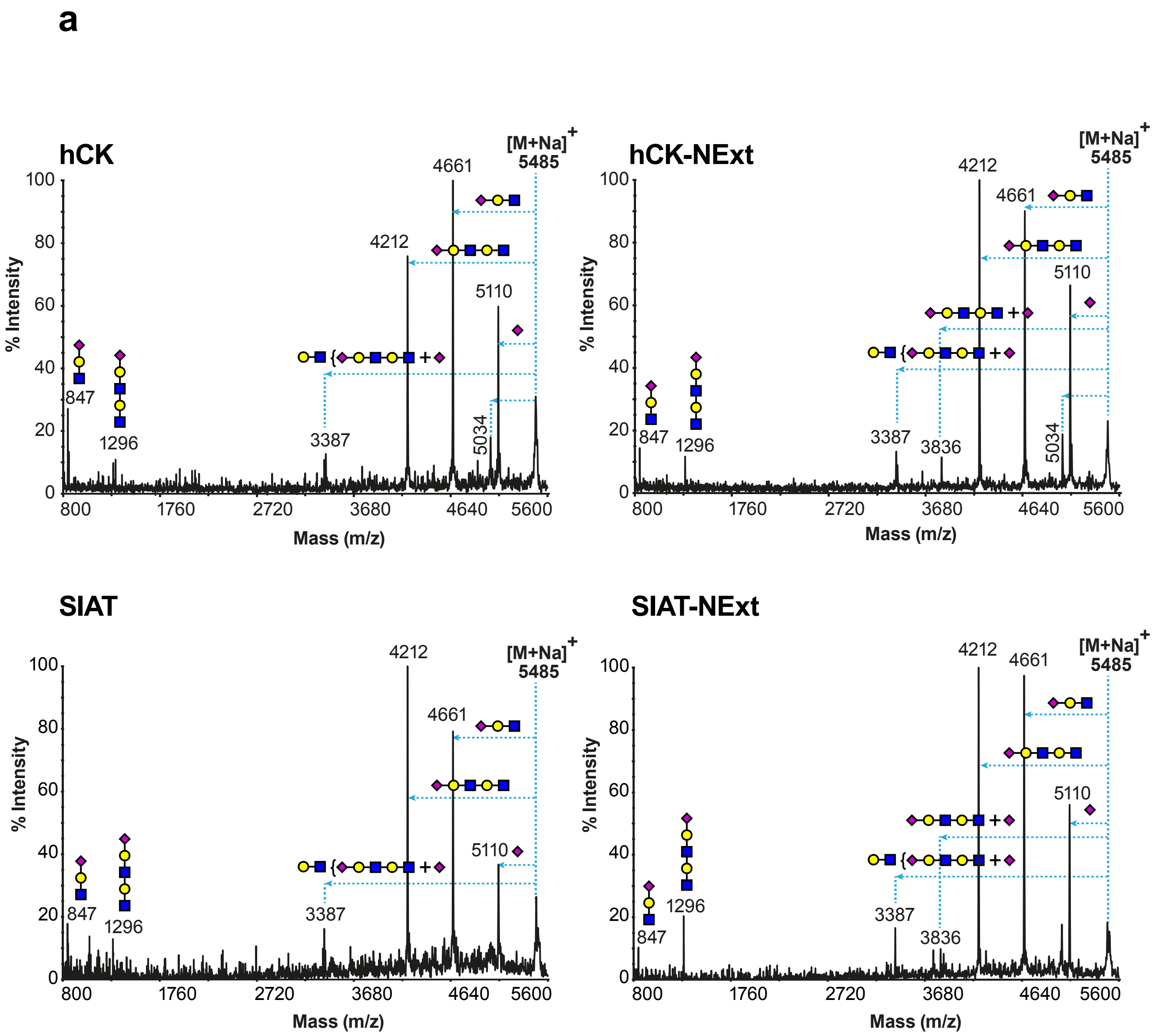

**b**

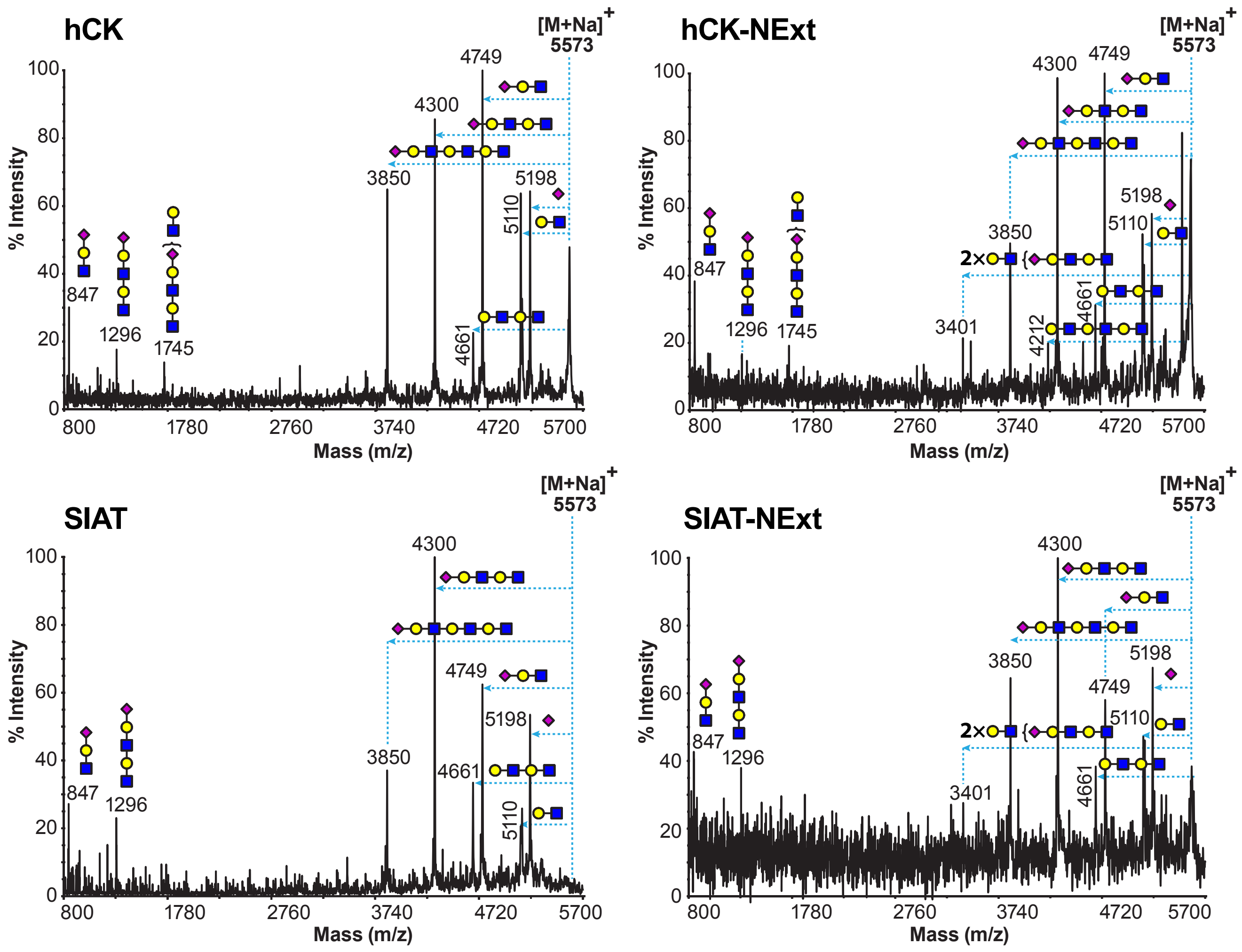

hCK
[M+Na]+
5573
4749
4300
3850
5198
5110
4661
847
1296
1745
% Intensity
Mass (m/z)
hCK-NExt
[M+Na]+
5573
4300
4749
3850
5198
5110
4661
4212
3401
2×
SIAT
[M+Na]+
5573
4300
4749
5198
3850
4661
5110
SIAT-NExt
[M+Na]+
5573
4300
3850
5198
4749
5110
4661
3401

c

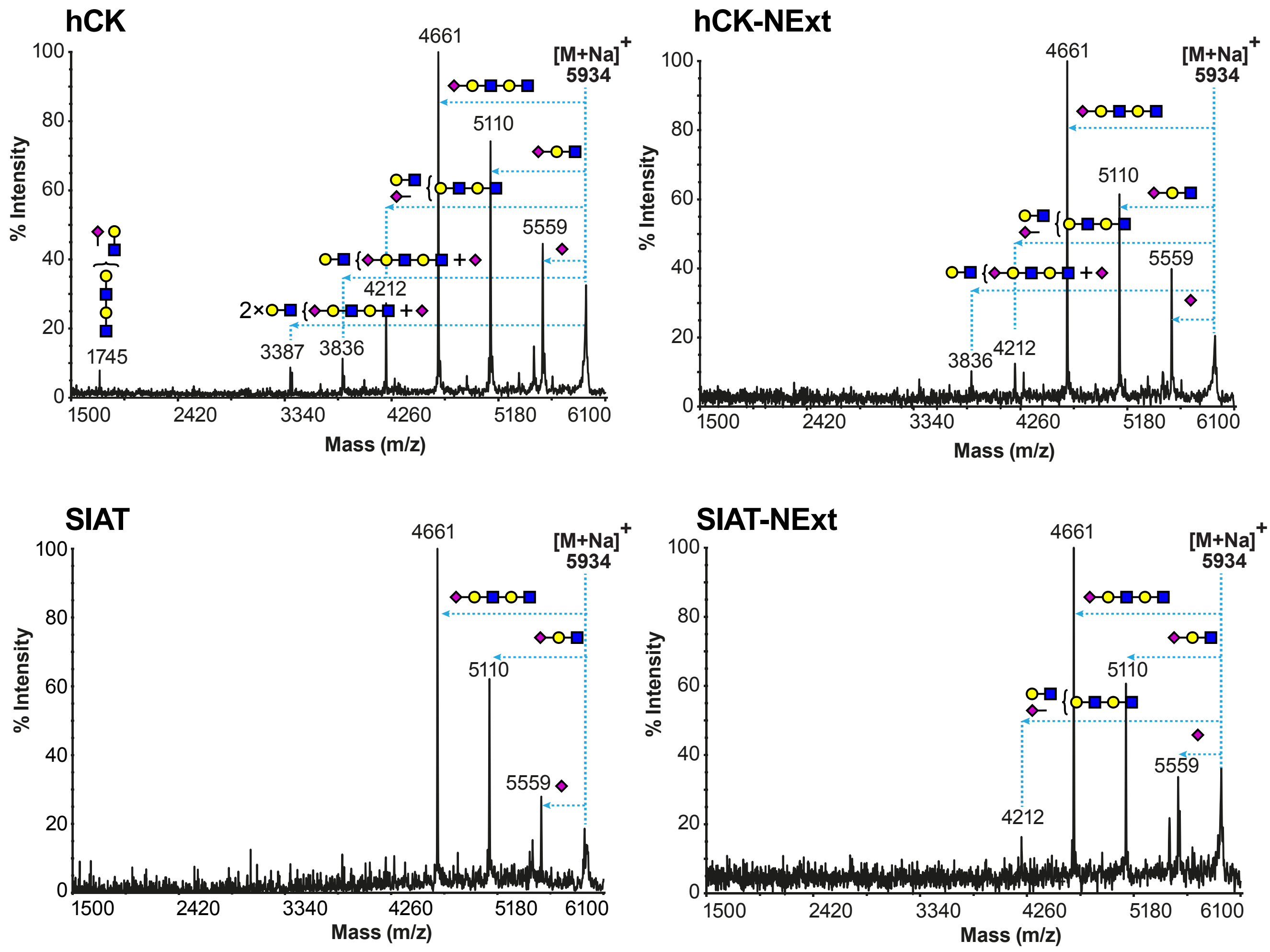
hCK
hCK-NExt
SIAT
SIAT-NExt
% Intensity
Mass (m/z)
[M+Na]+
5934
4661
5110
5559
4212
3836
3387
1745
2×
1500
2420
3340
4260
5180
6100
0
20
40
60
80
100

d

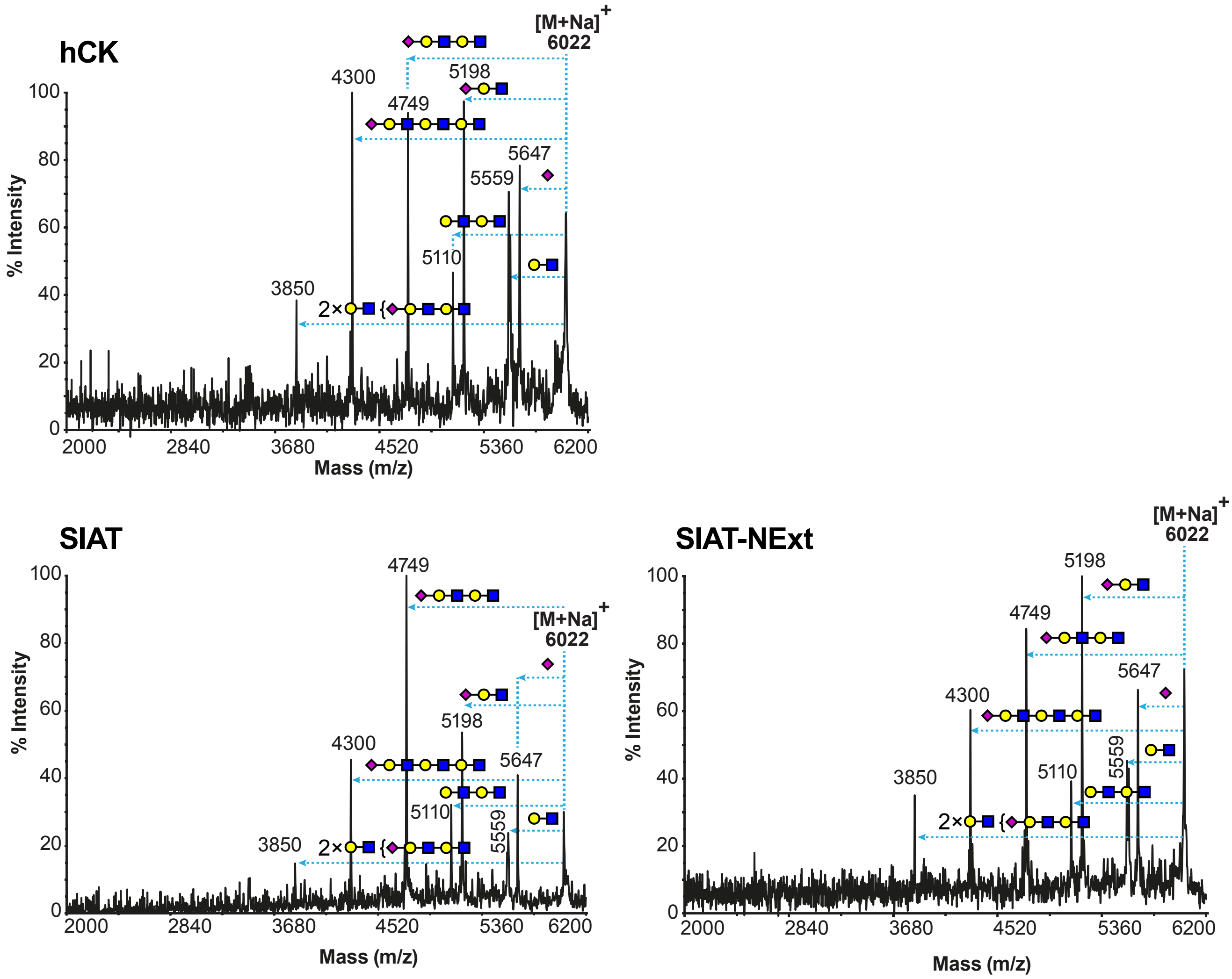
hCK
[M+Na]+
6022
100
80
60
40
20
0
% Intensity
4300
5198
4749
5647
5559
5110
3850
2×
2000
2840
3680
4520
5360
6200
Mass (m/z)
SIAT
4749
[M+Na]+
6022
5198
4300
5647
5110
5559
3850
2×
% Intensity
Mass (m/z)
SIAT-NExt
[M+Na]+
6022
5198
4749
5647
4300
5559
5110
3850
2×
% Intensity
Mass (m/z)

e

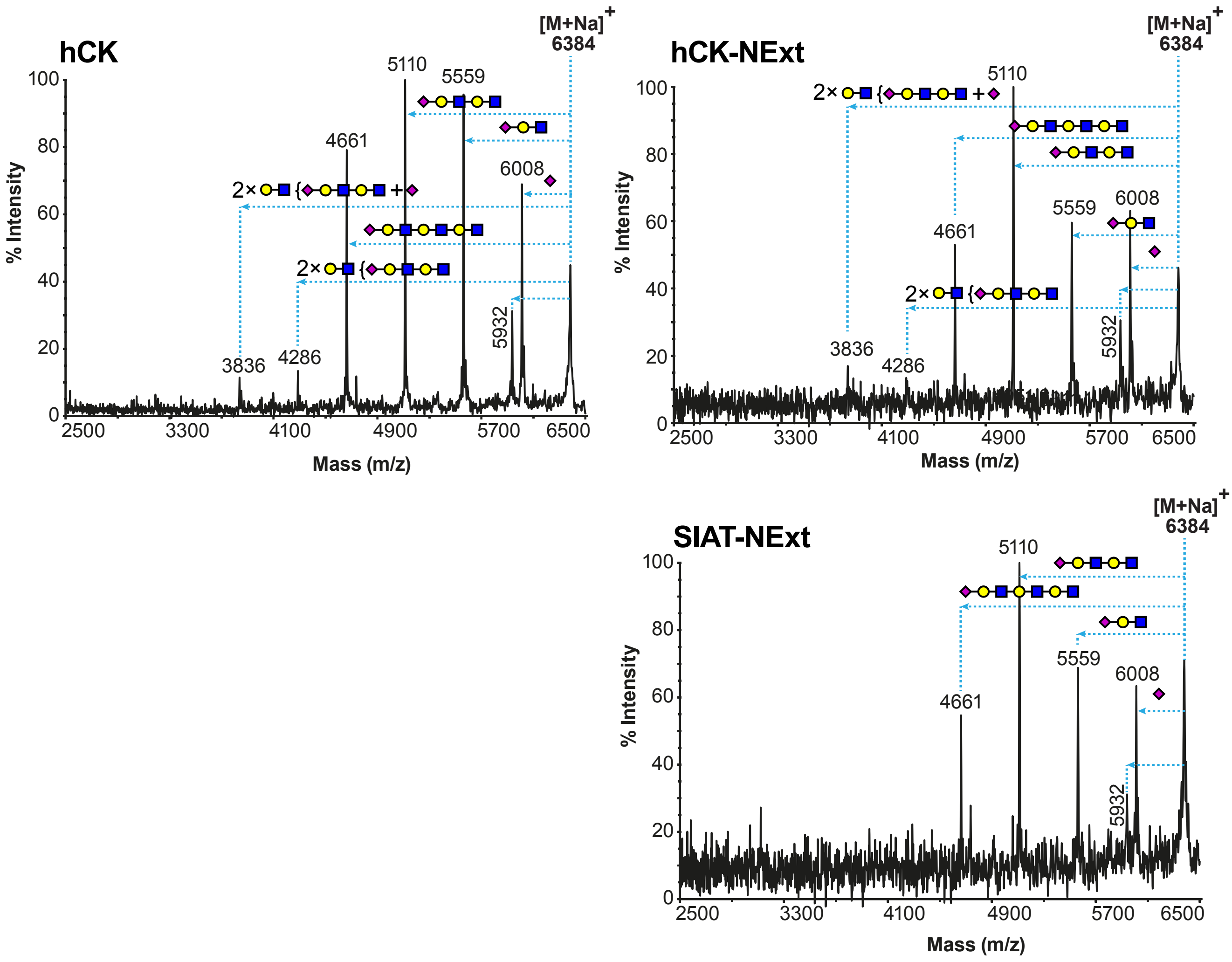

hCK
[M+Na]+
6384
5110
5559
4661
6008
2×
3836
4286
5932
% Intensity
Mass (m/z)
hCK-NExt
SIAT-NExt

f

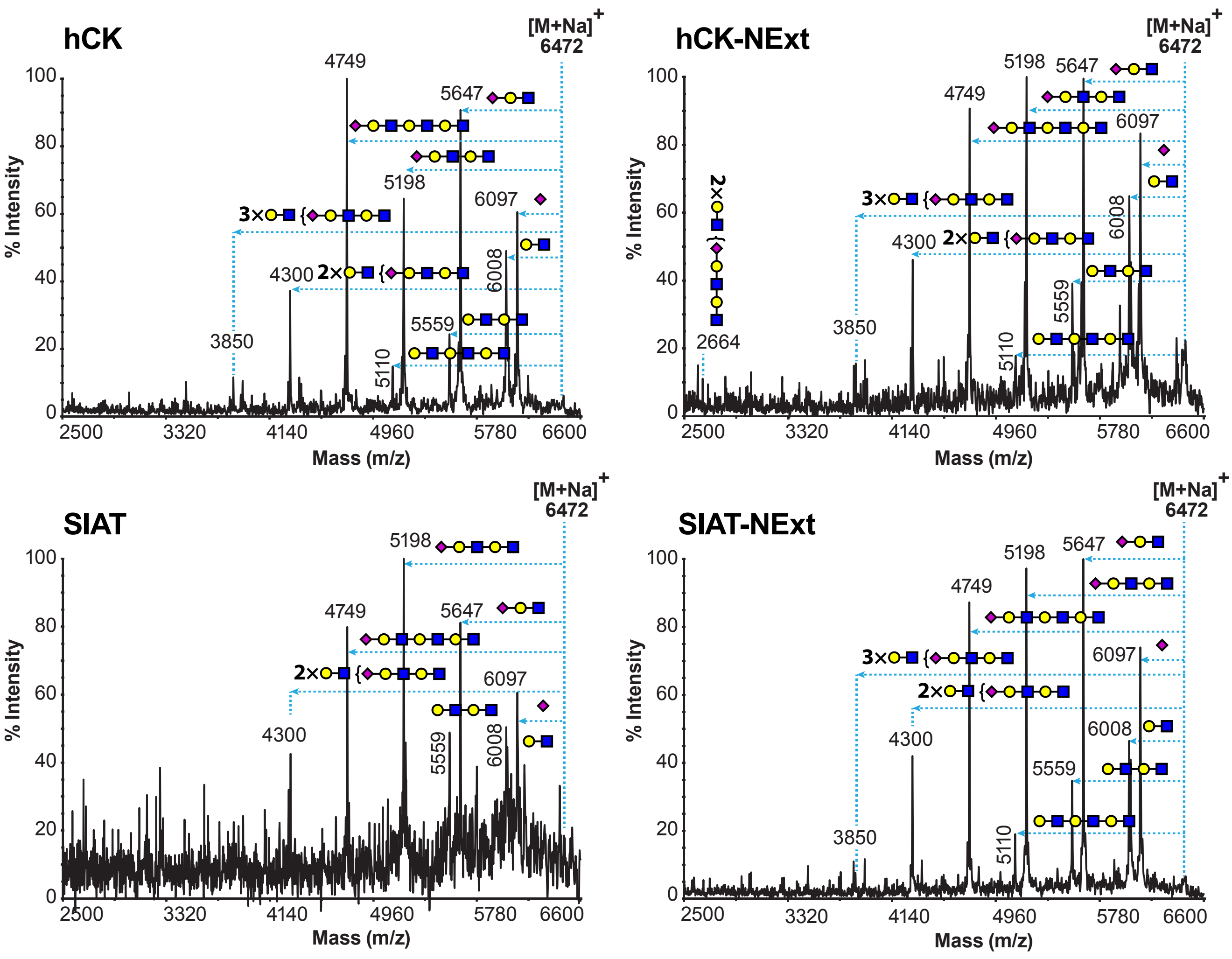
hCK
[M+Na]+
6472
% Intensity
Mass (m/z)
4749
5647
5198
6097
4300
3850
5559
5110
6008
3×
2×
hCK-NExt
[M+Na]+
6472
5198
5647
4749
6097
6008
4300
3850
2664
5559
5110
2×
3×
2×
SIAT
[M+Na]+
6472
5198
4749
5647
6097
4300
5559
6008
2×
SIAT-NExt
[M+Na]+
6472
5198
5647
4749
6097
6008
4300
5559
3850
5110
3×
2×

g

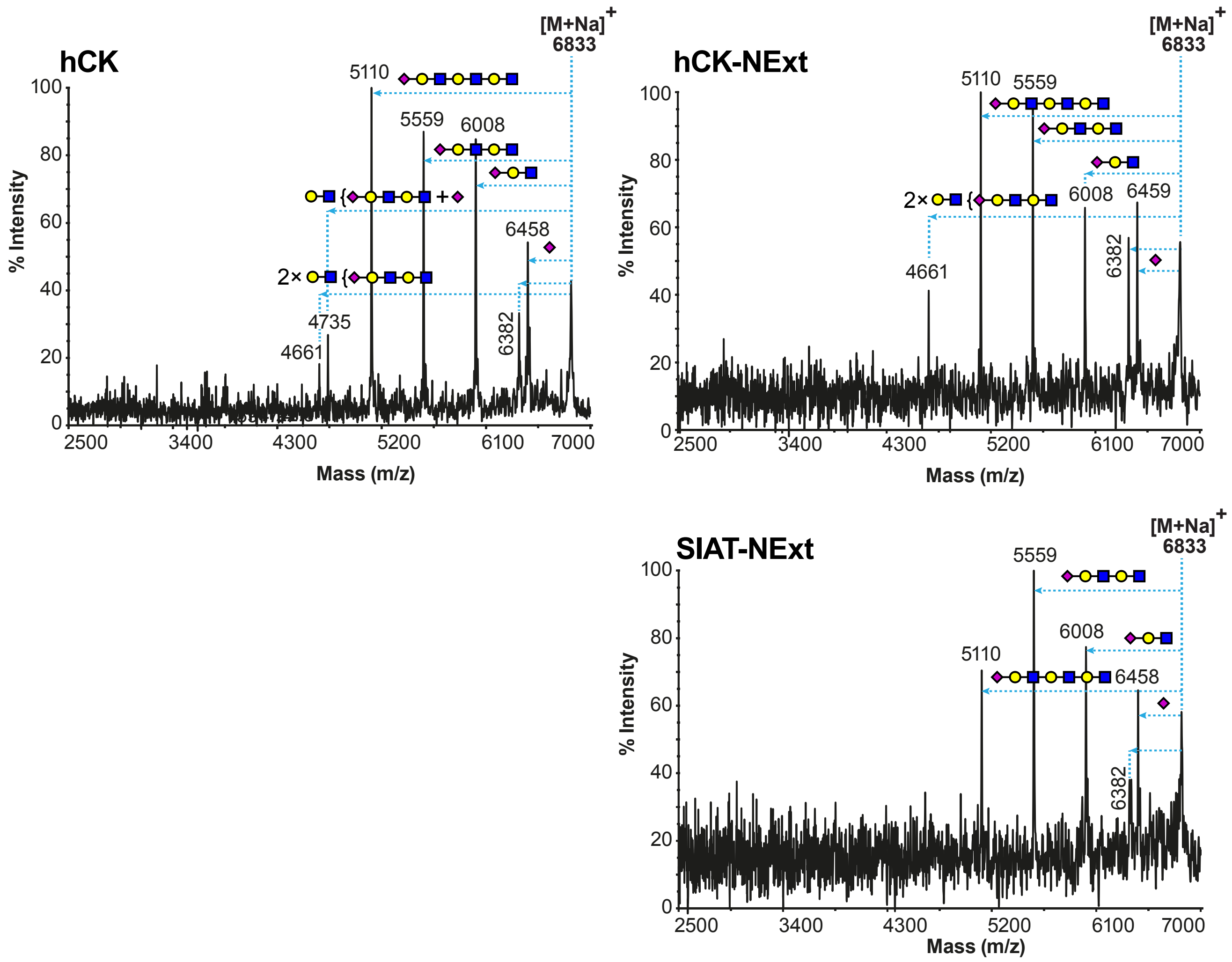
hCK
[M+Na]+
6833
5110
5559
6008
6458
6382
4735
4661
2×
% Intensity
Mass (m/z)
hCK-NExt
[M+Na]+
6833
5110
5559
6008
6459
6382
4661
2×
% Intensity
Mass (m/z)
SIAT-NExt
[M+Na]+
6833
5559
6008
5110
6458
6382
% Intensity
Mass (m/z)

**h**

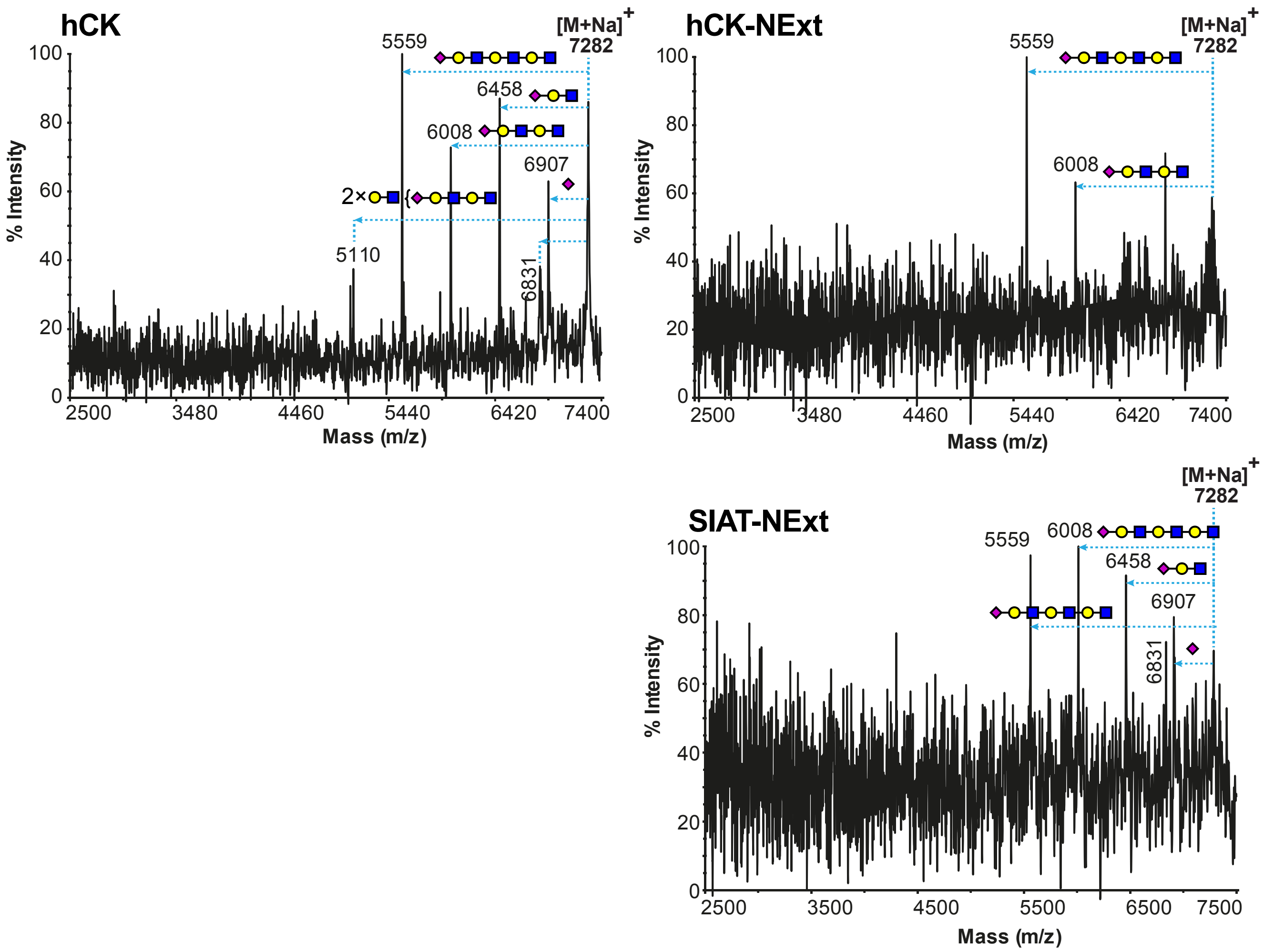
hCK
[M+Na]+
7282
5559
6458
6008
6907
2×
5110
6831
% Intensity
Mass (m/z)
hCK-NExt
SIAT-NExt

**Supplementary Figure 9. MALDI TOF/TOF MS/MS analysis of parental + Sial-S and NExt + Sial-S N-glycans isolated from hCK and SIAT cell lines.** Molecular ions $[M+Na]^+$ present in **Supplementary Fig. 7c** and **7d** (SIAT + Sial-S and SIAT-NExt + Sial-S respectively) and **Supplementary Fig. 10c** and **10d** (hCK + Sial-S and hCK-NExt. + Sial-S respectively) were selected for MALDI-TOF/TOF MS/MS analysis. (**a**) m/z 5485, (**b**) m/z 5573, (**c**) m/z 5934, (**d**) m/z 6022, (**e**) m/z 6384, (**f**) m/z 6472, (**g**) m/z 6833 and (**h**) m/z 7282. Horizontal dashed lines correspond to the indicated fragment loss from the corresponding molecular ion $[M+Na]^+$. Vertical dashed lines indicate the m/z fragment ion value that corresponds to the fragment loss as shown from the corresponding horizontal dashed line. Structures outside the bracket have not unequivocally been defined. For clarity, not all fragments are annotated.

# Supplementary Figure 10a

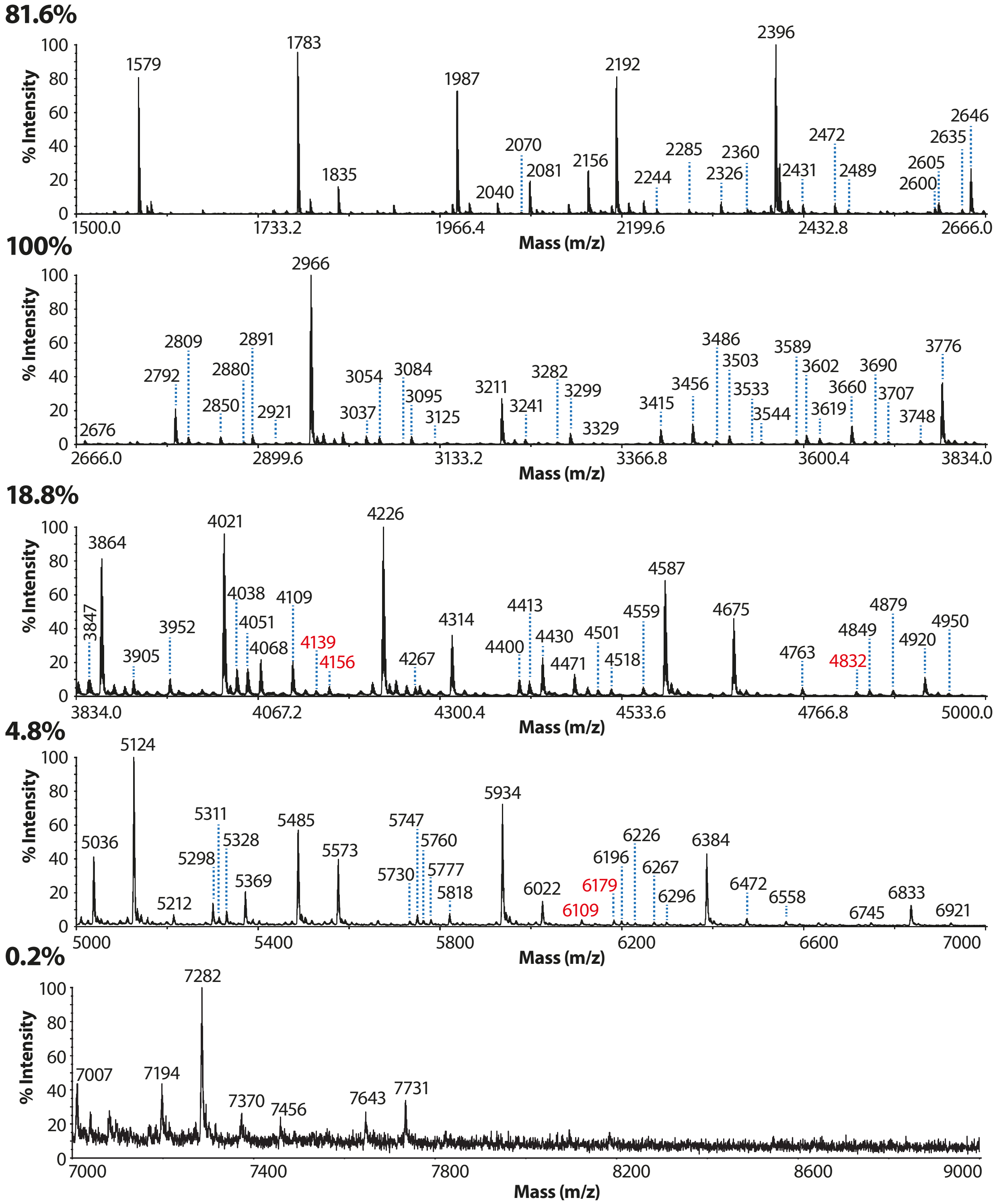

# Supplementary Figure 10b

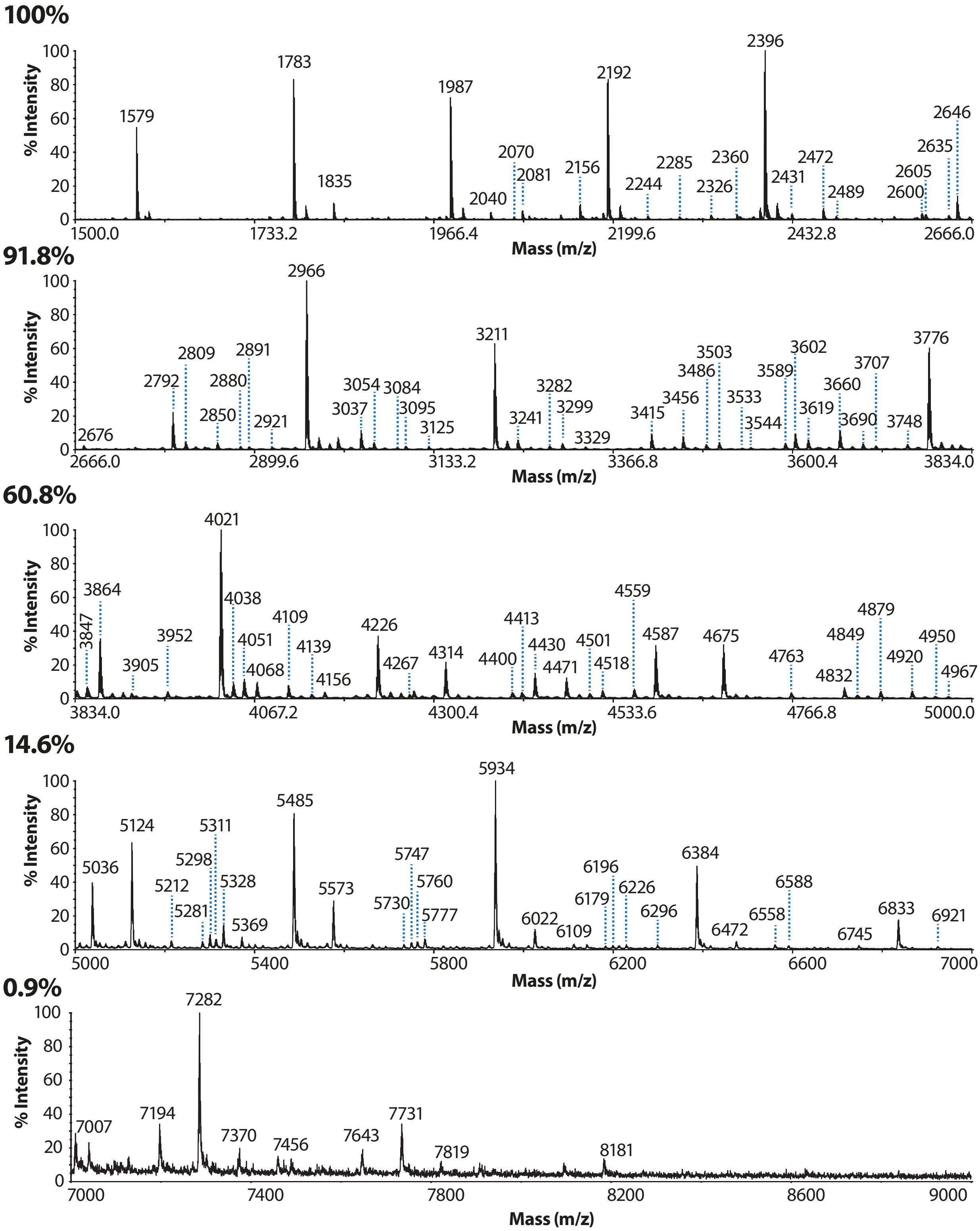

# Supplementary Figure 10c

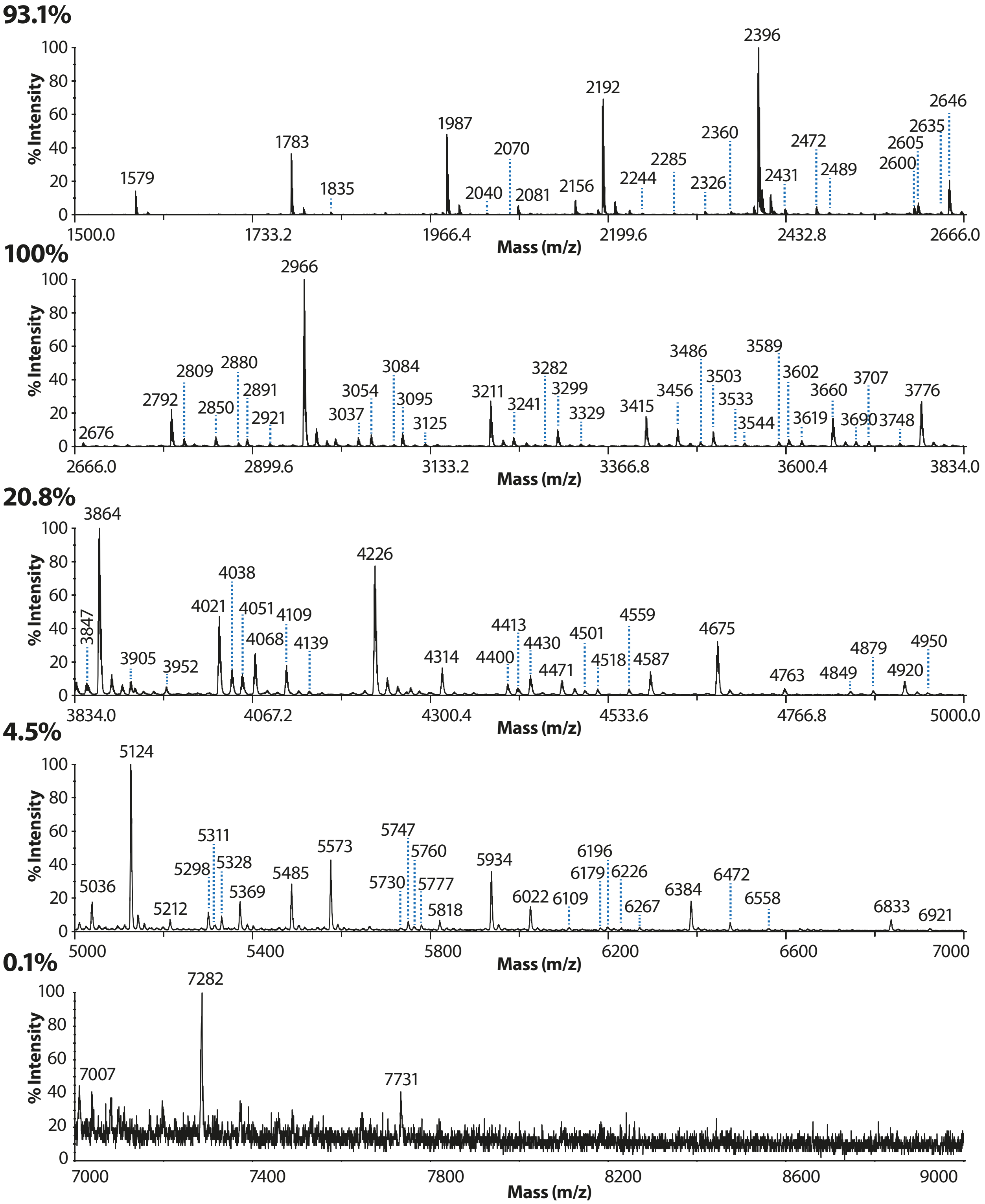

# Supplementary Figure 10d

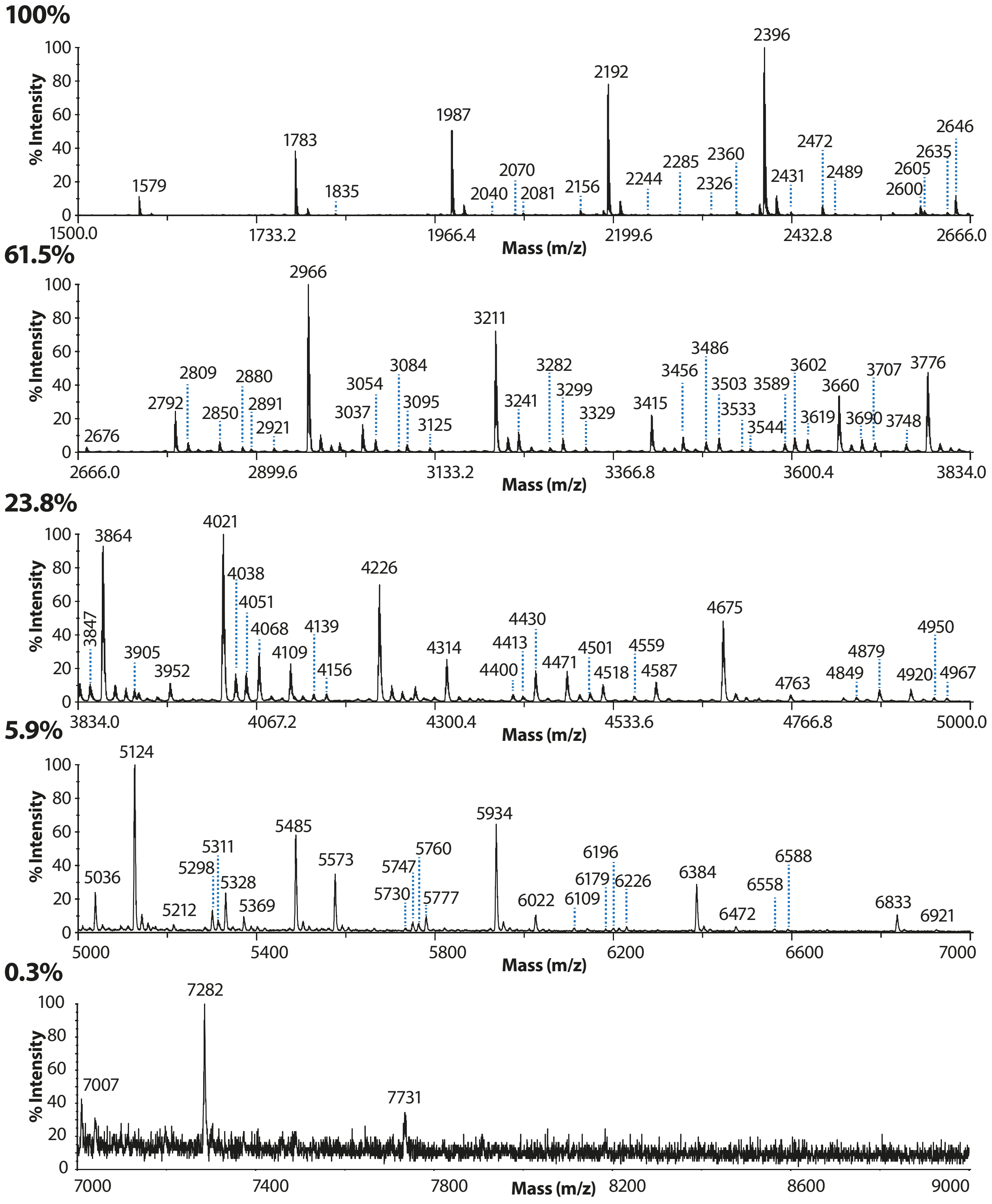

**Supplementary Figure 10. MALDI-TOF MS analysis of permethylated N-glycans of hCK and hCK-NExt cells, before or after the Sial-S digestion.** (**a**) hCK; (**b**) hCK-NExt; (**c**) hCK + Sial-S treatment; (**d**) hCK-NExt + Sial treatment. All molecular ions are $[M+Na]^+$. Putative structures are based on composition, tandem mass spectrometry, and biosynthetic knowledge. Percentages on top left of each panel correspond to the relative percentage of the maximum peak of the corresponding panel compared to the relative intensity of the maximum peak of the top panel. In (**a**) red values correspond to m/z values of the molecular ions that their relative abundance decreased after the Sial-S digestion, indication of α2-3-linked NeuAc residues. In (**c**) green peaks correspond to peaks with increased relative abundance after the Sial-S digestion. Note that the number of the peaks here are less than the number of peaks found in the MDCKs (**Supplementary Fig. 3a**) indication that the SIAT contain N-glycans with less α2-3-linked NeuAc residues. Full structure annotations can be found in **Supplementary Fig. 8**. MS spectra exported in ASCII format are provided as Source Data file.

# Supplementary Figure 11

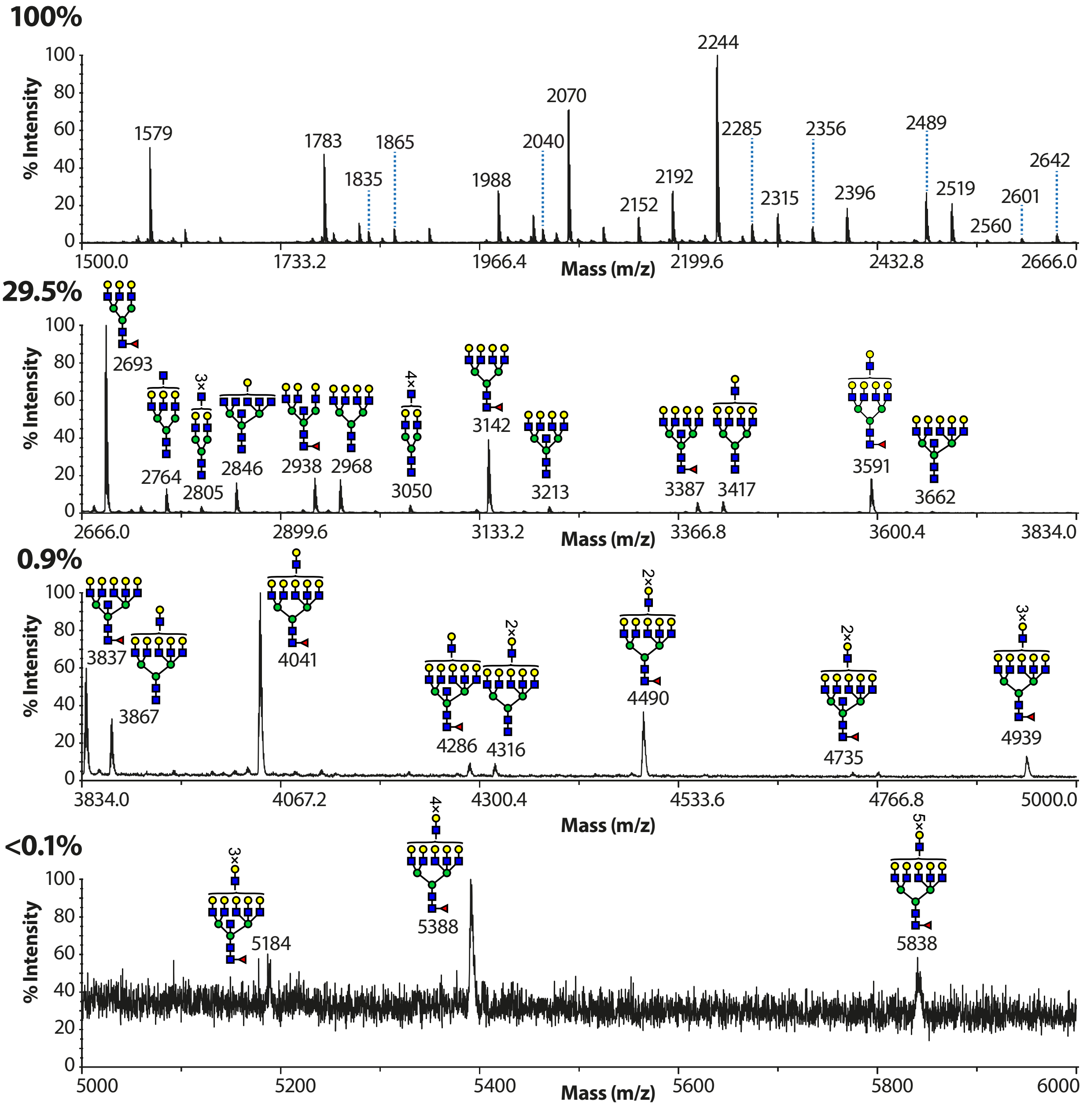

**Supplementary Figure 11. MALDI-TOF MS analysis of permethylated N-glycans from allantoic after sialidase A (Sial-A) treatment.** All molecular ions are $[M+Na]^+$. Putative structures are based on composition, tandem mass spectrometry, and biosynthetic knowledge. Percentages on top left of each panel correspond to the relative percentage of the maximum peak of the corresponding panel compared to the relative intensity of the maximum peak of the top panel. For comparison with the native glycome, compare with **Fig. 4a**. For decrease of the relative abundance of the α2-6-linked NeuAc residues, compare with **Fig. 4b**. MS spectra exported in ASCII format are provided as Source Data file.

# Supplementary Figure 12a

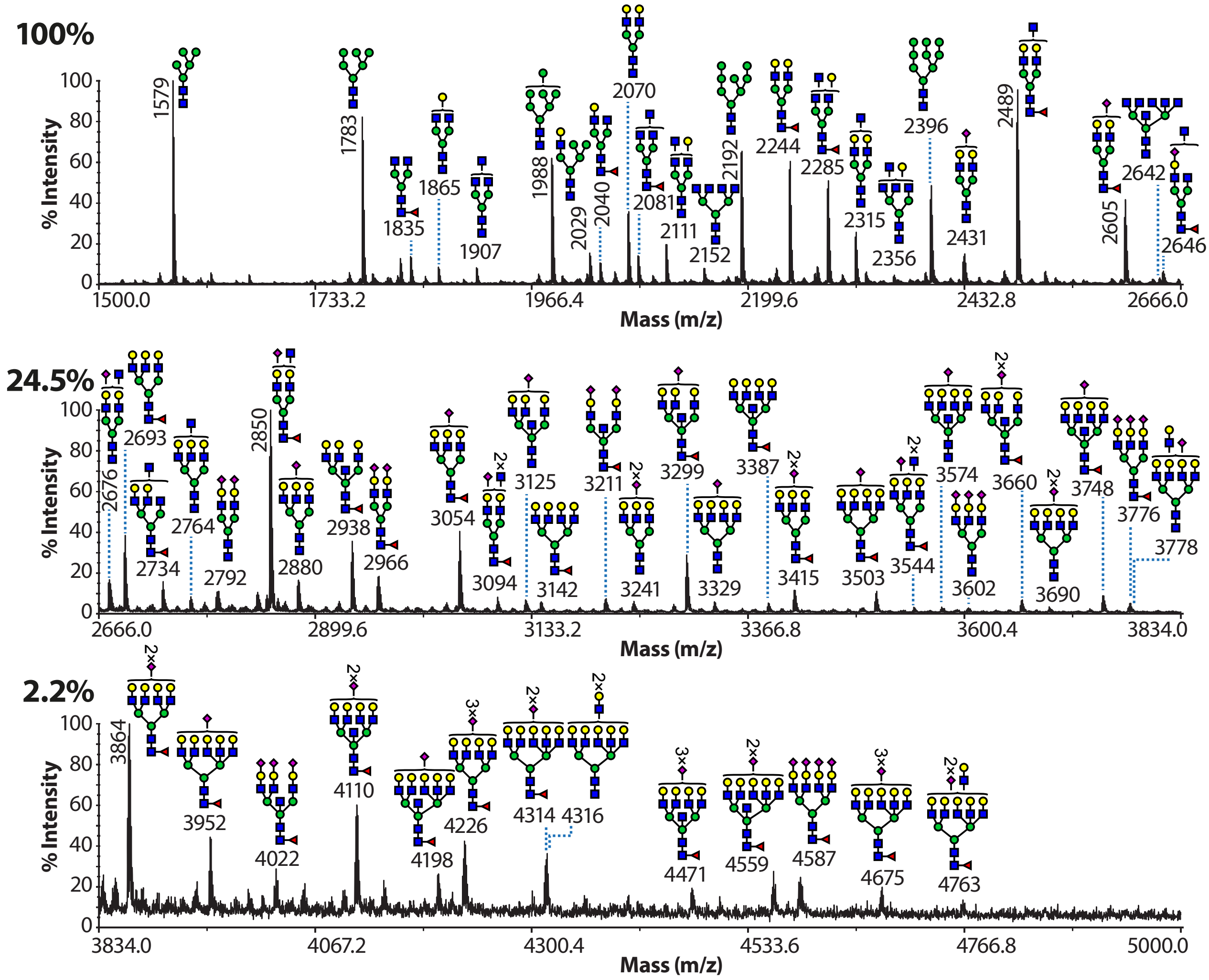

# Supplementary Figure 12b

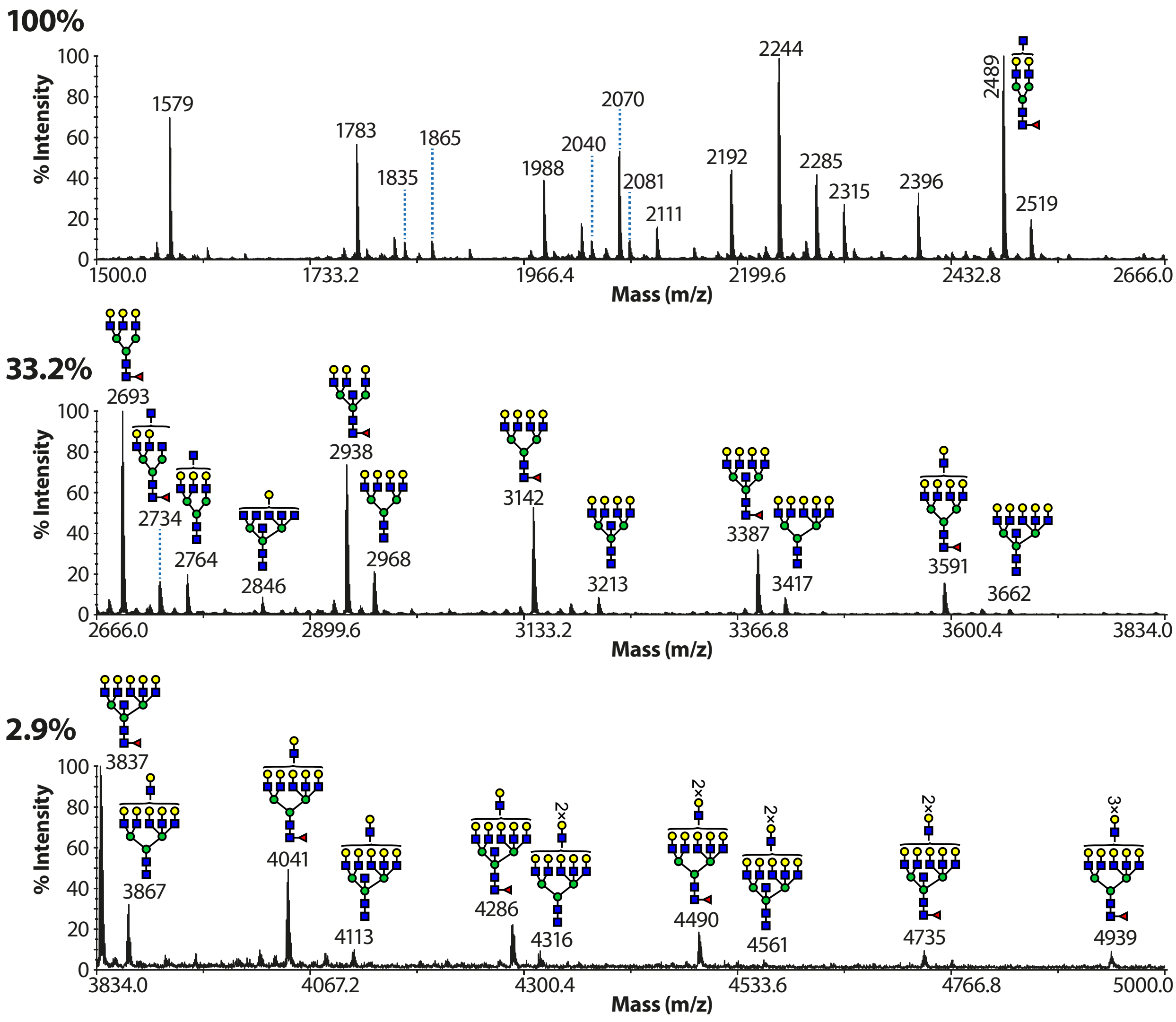

# Supplementary Figure 12c

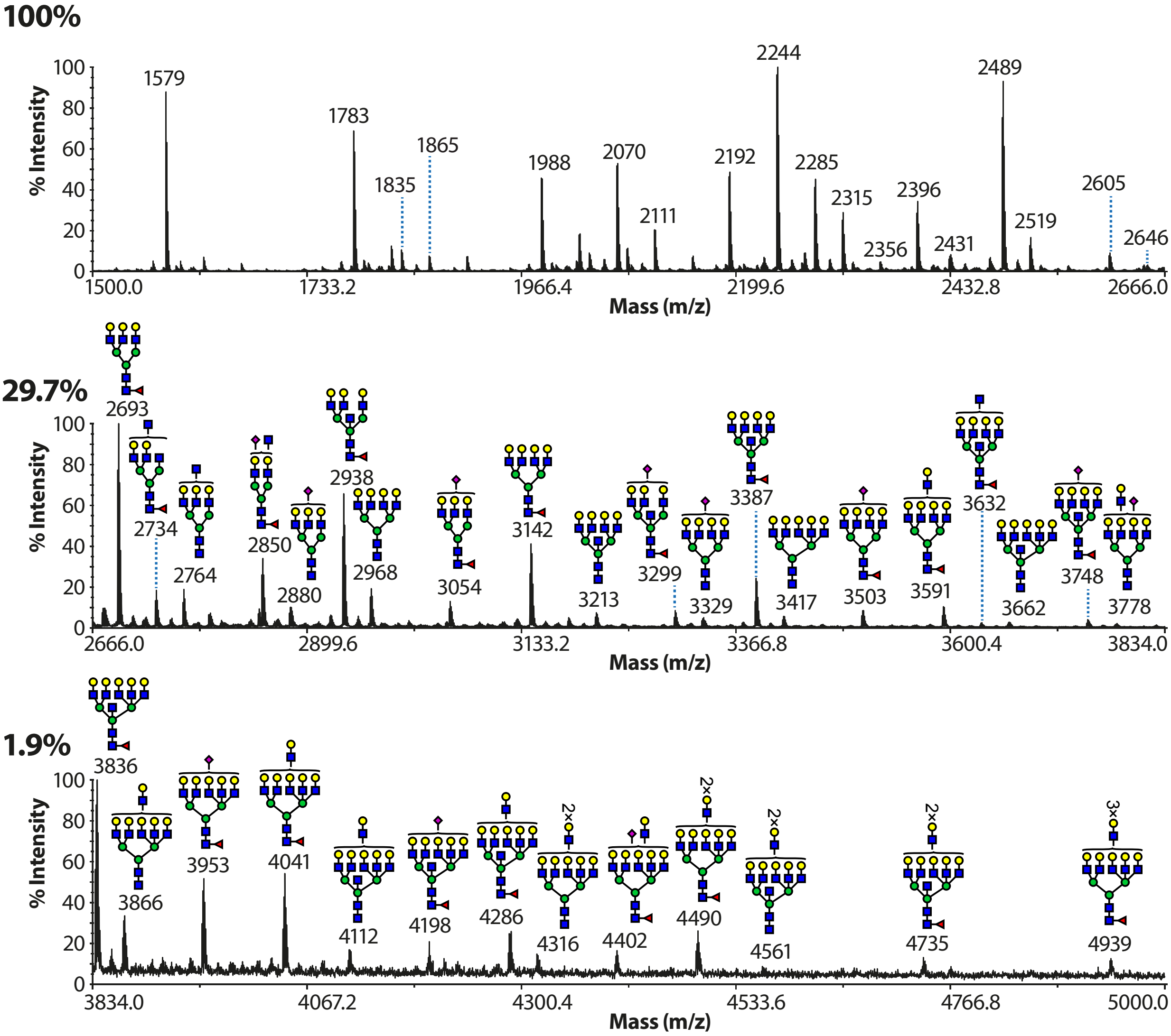

**Supplementary Figure 12. MALDI-TOF MS analysis of permethylated N-glycans from amniotic membrane, before or after sialidase treatment.** (**a**) amniotic membrane; (**b**) Sial-A treatment; (**c**) Sial-S treatment. All molecular ions are $[M+Na]^{+}$. Putative structures are based on composition, tandem mass spectrometry, and biosynthetic knowledge. Percentages on top left of each panel correspond to the relative percentage of the maximum peak of the corresponding panel compared to the relative intensity of the maximum peak of the top panel. Core-fucosylated structures had higher relative abundance when compared to their non-core-fucosylated counterparts (compare in **a**, m/z 2850 vs 2676, 3054 vs 2880, 3299 vs 3125, 3503 vs 3329). For the decrease of the relative abundance of the α2-3-linked NeuAc residues, compare in amniotic (**c** vs **a**), the decrease of the relative abundance of the molecular ions which correspond to tri- antennary N-glycans with various levels of NeuAc residues (m/z 3054, 3415 and 3776) compared to the base peak tri-antennary N-glycan without any NeuAc residue (m/z 2693). Similarly, compare the relative abundance of the tetra-antannary N-glycans with various levels of NeuAc residues (m/z 3503, 3864, 4314 and 4587) compared to the base peak tetra-antennary N-glycan without any NeuAc residue (m/z 3142). MS spectra exported in ASCII format  are provided as Source Data file.

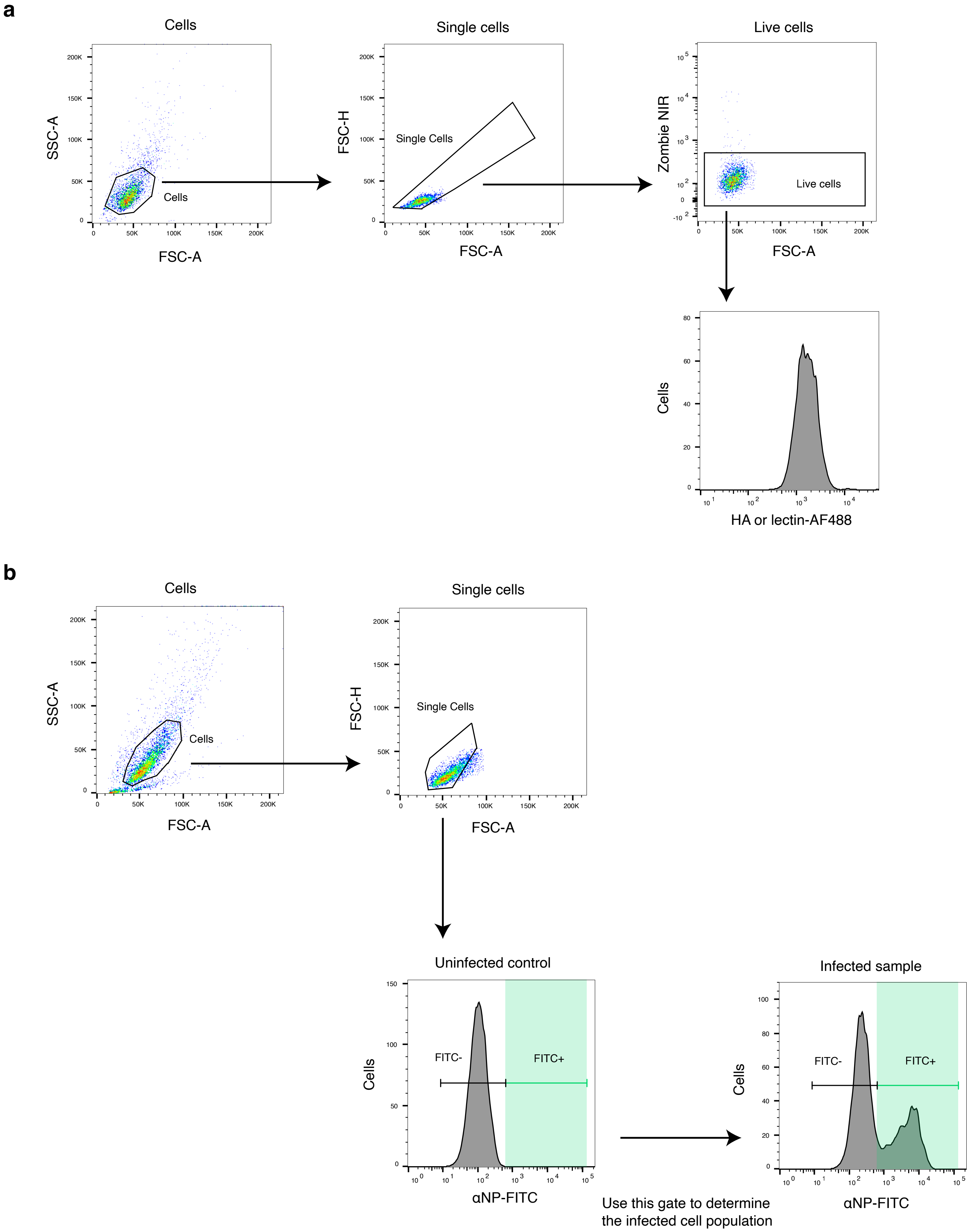
a
Cells
SSC-A
FSC-A
Cells
Single cells
FSC-H
Single Cells
FSC-A
Live cells
Zombie NIR
Live cells
FSC-A
Cells
HA or lectin-AF488
b
Cells
SSC-A
Cells
FSC-A
Single cells
FSC-H
Single Cells
FSC-A
Uninfected control
Cells
FITC-
FITC+
αNP-FITC
Use this gate to determine
the infected cell population
Infected sample
Cells
FITC-
FITC+
αNP-FITC

**Supplementary Figure 13. Gating strategies for flow cytometric analyses.** (**a**) Gating strategy for the HA and lectin binding assays (**Fig. 1b, Fig. 2 a-c, Supplementary Fig. 1, 2**). (**b**) Gating stragegy for the single-round infectivity assays (**Fig. 5a-b**). The FITC positive/negative gate was set by uninfected control and used for identifying the infected cells.
