# Supplementary material for: Glyco-engineered MDCK cells display preferred receptors of H3N2 influenza absent in eggs used for vaccines
Source: Nat Commun. 2023 Oct 4;14:6178. doi: 10.1038/s41467-023-41908-0 (PMC10551000; doi:10.1038/s41467-023-41908-0)
Supplement: Supplementary file 3 — Reporting Summary [file 41467_2023_41908_MOESM3_ESM.pdf]

nature portfolio

Corresponding author(s): Stuart M. Haslam, James C. Paulson

Last updated by author(s): Aug 7, 2023

Reporting Summary

Nature Portfolio wishes to improve the reproducibility of the work that we publish. This form provides structure for consistency and transparency in reporting. For further information on Nature Portfolio policies, see our Editorial Policies and the Editorial Policy Checklist.

Statistics

For all statistical analyses, confirm that the following items are present in the figure legend, table legend, main text, or Methods section.

n/a	Confirmed	
☐	☒	The exact sample size (*n*) for each experimental group/condition, given as a discrete number and unit of measurement
☐	☒	A statement on whether measurements were taken from distinct samples or whether the same sample was measured repeatedly
☐	☒	The statistical test(s) used AND whether they are one- or two-sided<br>*Only common tests should be described solely by name; describe more complex techniques in the Methods section.*
☒	☐	A description of all covariates tested
☐	☒	A description of any assumptions or corrections, such as tests of normality and adjustment for multiple comparisons
☐	☒	A full description of the statistical parameters including central tendency (e.g. means) or other basic estimates (e.g. regression coefficient) AND variation (e.g. standard deviation) or associated estimates of uncertainty (e.g. confidence intervals)
☐	☒	For null hypothesis testing, the test statistic (e.g. *F*, *t*, *r*) with confidence intervals, effect sizes, degrees of freedom and *P* value noted<br>*Give P values as exact values whenever suitable.*
☒	☐	For Bayesian analysis, information on the choice of priors and Markov chain Monte Carlo settings
☒	☐	For hierarchical and complex designs, identification of the appropriate level for tests and full reporting of outcomes
☒	☐	Estimates of effect sizes (e.g. Cohen's *d*, Pearson's *r*), indicating how they were calculated

Our web collection on statistics for biologists contains articles on many of the points above.

Software and code

Policy information about availability of computer code

Data collection	Flow cytometry: ZE5 analyzer, Everest<br>Plate reader: BioTek Synergy H1, Gen5 v3.08
Data analysis	Flowjo v10.8.1<br>Fiji (ImageJ2) v2.9.0<br>ilastik v1.4.0<br>GraphPad Prism 9 v9.5.0<br>Microsoft Excel v16.69.1<br>OriginLab 2022<br>Figure layout: Adobe Illustrator 2021, 2022, 2023<br>Mass spectrometry: 4000 Series Explorer (ver. 3.5.3, build 1017), Data Explorer (ver. 4.9, build 115), Glycoworkbench (ver. 2.1, build 146)

For manuscripts utilizing custom algorithms or software that are central to the research but not yet described in published literature, software must be made available to editors and reviewers. We strongly encourage code deposition in a community repository (e.g. GitHub). See the Nature Portfolio guidelines for submitting code & software for further information.

Data

Policy information about availability of data

All manuscripts must include a data availability statement. This statement should provide the following information, where applicable:

- Accession codes, unique identifiers, or web links for publicly available datasets
- A description of any restrictions on data availability
- For clinical datasets or third party data, please ensure that the statement adheres to our policy

All data are available in the article and Supplementary files or Source Data. Materials produced in this research can also be accessed by reaching out to the corresponding authors.

Human research participants

Policy information about studies involving human research participants and Sex and Gender in Research.

Reporting on sex and gender	N/A
Population characteristics	N/A
Recruitment	N/A
Ethics oversight	N/A

Note that full information on the approval of the study protocol must also be provided in the manuscript.

Field-specific reporting

Please select the one below that is the best fit for your research. If you are not sure, read the appropriate sections before making your selection.

☒ Life sciences ☐ Behavioural & social sciences ☐ Ecological, evolutionary & environmental sciences

For a reference copy of the document with all sections, see nature.com/documents/nr-reporting-summary-flat.pdf

Life sciences study design

All studies must disclose on these points even when the disclosure is negative.

Sample size	Sample/group sizes ≥ 3 were chosen to support statistical analysis and variation expected based on similar published experiments
Data exclusions	In quantitation of plaque size, small plaques that could not be distinguished from background stain were not used in the analysis.
Replication	All flow cytometry, ELISA, virus growth data had minimum of triplicate technical replicates and entire experiments were minimally reproduced twice. The number of repeats and sample sizes are provided in each figure legend.
Randomization	The randomization of samples were not done since experiments were conducted using different cell lines and/or viruses. All experiments were done with appropriate controls that were relied upon as reference points to quantitatively assess differences between groups.
Blinding	Investigators were not blinded during experimental setup, data collection, and analyses, experiments always included relevant controls to assess statistical differences between groups.

Reporting for specific materials, systems and methods

We require information from authors about some types of materials, experimental systems and methods used in many studies. Here, indicate whether each material, system or method listed is relevant to your study. If you are not sure if a list item applies to your research, read the appropriate section before selecting a response.

Materials & experimental systems

n/a	Involved in the study
☐	☒ Antibodies
☐	☒ Eukaryotic cell lines
☒	☐ Palaeontology and archaeology
☒	☐ Animals and other organisms
☒	☐ Clinical data
☒	☐ Dual use research of concern

Methods

n/a	Involved in the study
☒	☐ ChIP-seq
☐	☒ Flow cytometry
☒	☐ MRI-based neuroimaging

Antibodies

Antibodies used

Fc Receptor Binding Inhibitor Polyclonal Antibody, eBioscience (Thermo Fisher Scientific, Cat# 14-9162-42, RRID:AB_2572935)
Ultra-LEAF™ Purified anti-His Tag Antibody (BioLegend, J095G46, Cat# 362616, RRID:AB_2814311)
Alexa Fluor® 488 anti-mouse IgG2a Antibody (BioLegend , RMG2a-62, Cat# 407122, RRID:AB_2721368)
Goat anti-Mouse IgG (H+L) Cross-Adsorbed Secondary Antibody, HRP (Thermo Fisher Scientific, Cat# G-21040, RRID:AB_2536527)
InVivoMAb anti-Influenza A virus NP (BioXCell, H16-L10-4R5 (HB-65), Cat# BE0159, RRID:AB_10949071)
Goat Anti-Mouse IgG Fc-HRP (SouthernBiotech, Cat# 1033-05, RRID:AB_2737432)
Influenza A NP Monoclonal Antibody (D67J), FITC (Thermo Fisher Scientific, Cat# MA1-7322, RRID:AB_1017747)

Validation

All antibodies are validated by the manufacturers and previous studies of others.
Ultra-LEAF™ Purified anti-His Tag Antibody (BioLegend, J095G46, Cat# 362616, RRID:AB_2814311) - Each lot of this antibody is quality control tested by Western blotting. Verified for flow cytometry.
InVivoMAb anti-Influenza A virus NP (BioXCell, H16-L10-4R5 (HB-65), Cat# BE0159, RRID:AB_10949071) - Reported application including: Immunohistochemistry (paraffin), Western blot, Flow cytometry. Used for plaque assays for H3N2 viruses in literature.
Influenza A NP Monoclonal Antibody (D67J), FITC (Thermo Fisher Scientific, Cat# MA1-7322, RRID:AB_1017747) - The MA1-7322 antibody reacts with Influenza A virus NP in viral and infected tissue samples. MA1-7322 has been successfully used in immunofluorescence, FACS, and ELISA applications.

For each experiments, 'secondary only' control was performed to examine potential background staining.

Eukaryotic cell lines

Policy information about cell lines and Sex and Gender in Research

Cell line source(s)

Madin-Darby canine kidney (MDCK) cells (ATCC,Cat# CCL-34) was isolated from a female dog. All other cell lines are derivative.
MDCK-SIAT1 cells (Sigma-Aldrich, Cat# 05071502-1VL)
hCK cells (Takada, K., Kawakami, C., Fan, S., Chiba, S., Zhong, G., Gu, C., ... & Kawaoka, Y. (2019). A humanized MDCK cell line for the efficient isolation and propagation of human influenza viruses. Nature microbiology, 4(8), 1268-1273.) Obtained from Kawaoka Lab.
FreeStyle 293F cells Thermo Scientific Cat# R79007

Authentication

Cell lines were not authenticated.

Mycoplasma contamination

Cell lines were not tested for mycoplasma contamination.

Commonly misidentified lines
(See ICLAC register)

No commonly misidentified lines were used in this study.

Flow Cytometry

Plots

Confirm that:

☒ The axis labels state the marker and fluorochrome used (e.g. CD4-FITC).

☒ The axis scales are clearly visible. Include numbers along axes only for bottom left plot of group (a 'group' is an analysis of identical markers).

☒ All plots are contour plots with outliers or pseudocolor plots.

☒ A numerical value for number of cells or percentage (with statistics) is provided.

Methodology

Sample preparation

For SNA/HA binding study: A monolayer of cultured cells were detached and digested to single cells by TrypLE Express Enzyme (Gibco). Cells were washed with PBS and stained with Zombie NIR dye (BioLegend) for Live/Dead staining. After washing with PBS three times, cells were blocked with canine Fc block (Invitrogen) on ice for 10 min. The cells were subsequently incubated with respective staining reagents on ice for 45 min: biotinylated SNA precomplexed with Avidin-

	Alexa fluor 488; recombinant 50 µg/mL HAs precomplexed with anti-His mouse IgG2a and Alexa fluor 488-conjugated anti-mouse IgG2a (4:2:1 w/w/w). The cells were washed twice with PBS before resuspended in PBS containing 1% BSA for flow cytometry.<br><br>For single-round infectivity study: Cells were collected with trypsin, fixed and permeabilized with the FOXP3 Fix/Perm Buffer Set (BioLegend) following the manufacturer's protocol, and stained with FITC-conjugated anti-NP monoclonal antibody (1:100, ThermoFisher) for 45 minutes on ice. Cells were washed twice with perm buffer before being resuspended in PBS containing 1% BSA for flow cytometry to obtain the ratio of NP-positive cells.
Instrument	ZE5 Cell Analyzer (Bio-Rad)
Software	Analysis was performed in FlowJo
Cell population abundance	At least 3000 events in the final gate were collected.
Gating strategy	For SNA/HA binding study: Single cells (FSC/SSC) -> Live cells<br>For single-round infectivity study: Single cells (FSC/SSC) then negative and positive staining gates were determined by uninfected controls.<br>It is shown in Supplementary Fig. 13.

☒ Tick this box to confirm that a figure exemplifying the gating strategy is provided in the Supplementary Information.
