# Supplementary material for: Glyco-engineered MDCK cells display preferred receptors of H3N2 influenza absent in eggs used for vaccines
Source: Nat Commun. 2023 Oct 4;14:6178. doi: 10.1038/s41467-023-41908-0 (PMC10551000; doi:10.1038/s41467-023-41908-0)
Supplement: Supplementary file 4 — Description of Additional Supplementary Files [file 41467_2023_41908_MOESM4_ESM.pdf]

Description of Additional Supplementary Files

Supplementary Data 1

Description: Molecular ion intensity data is provided for MALDI-TOF MS spectra of glycans before and after sialidase treatment from MDCK and MDCK-NExt cells in Supplementary Fig. 3, SIAT and SIAT-NExt cells in Supplementary Fig. 7, and hCK and hCK-NExt cells in Supplementary Fig. 10. For each analysis, molecular ion intensities are shown for peaks corresponding to complex N-glycans (m/z 2966 and above). In addition, to show the relative abundance of each molecular ion the intensity of each peak is normalized relative to the intensity of m/z 2966, and to the sum of their intensities.
